# Popular interest in vertebrates does not reflect extinction risk and is associated with bias in conservation investment

**DOI:** 10.1371/journal.pone.0203694

**Published:** 2018-09-26

**Authors:** Thomas Davies, Andrew Cowley, Jon Bennie, Catherine Leyshon, Richard Inger, Hazel Carter, Beth Robinson, James Duffy, Stefano Casalegno, Gwladys Lambert, Kevin Gaston

**Affiliations:** 1 School of Ocean Sciences, Bangor University, Menai Bridge, Anglesey, United Kingdom; 2 Centre for Geography Environment and Society, University of Exeter, Penryn, Cornwall, United Kingdom; 3 Environment and Sustainability Institute, University of Exeter, Penryn, Cornwall, United Kingdom; 4 C/O Environment and Sustainability Institute, University of Exeter, Penryn, Cornwall, United Kingdom; 5 Alaska Fisheries Science Center, National Marine Fisheries Service, Seattle, Washington, United States of America; INIBIOMA (Universidad Nacional del Comahue-CONICET), ARGENTINA

## Abstract

The interrelationship between public interest in endangered species and the attention they receive from the conservation community is the ‘flywheel’ driving much effort to abate global extinction rates. Yet big international conservation non-governmental organisations have typically focused on the plight of a handful of appealing endangered species, while the public remains largely unaware of the majority. We quantified the existence of bias in popular interest towards species, by analysing global internet search interest in 36,873 vertebrate taxa. Web search interest was higher for mammals and birds at greater risk of extinction, but this was not so for fish, reptiles and amphibians. Our analysis reveals a global bias in popular interest towards vertebrates that is undermining incentives to invest financial capital in thousands of species threatened with extinction. Raising the popular profile of these lesser known endangered and critically endangered species will generate clearer political and financial incentives for their protection.

## Background

**Fig 1:**
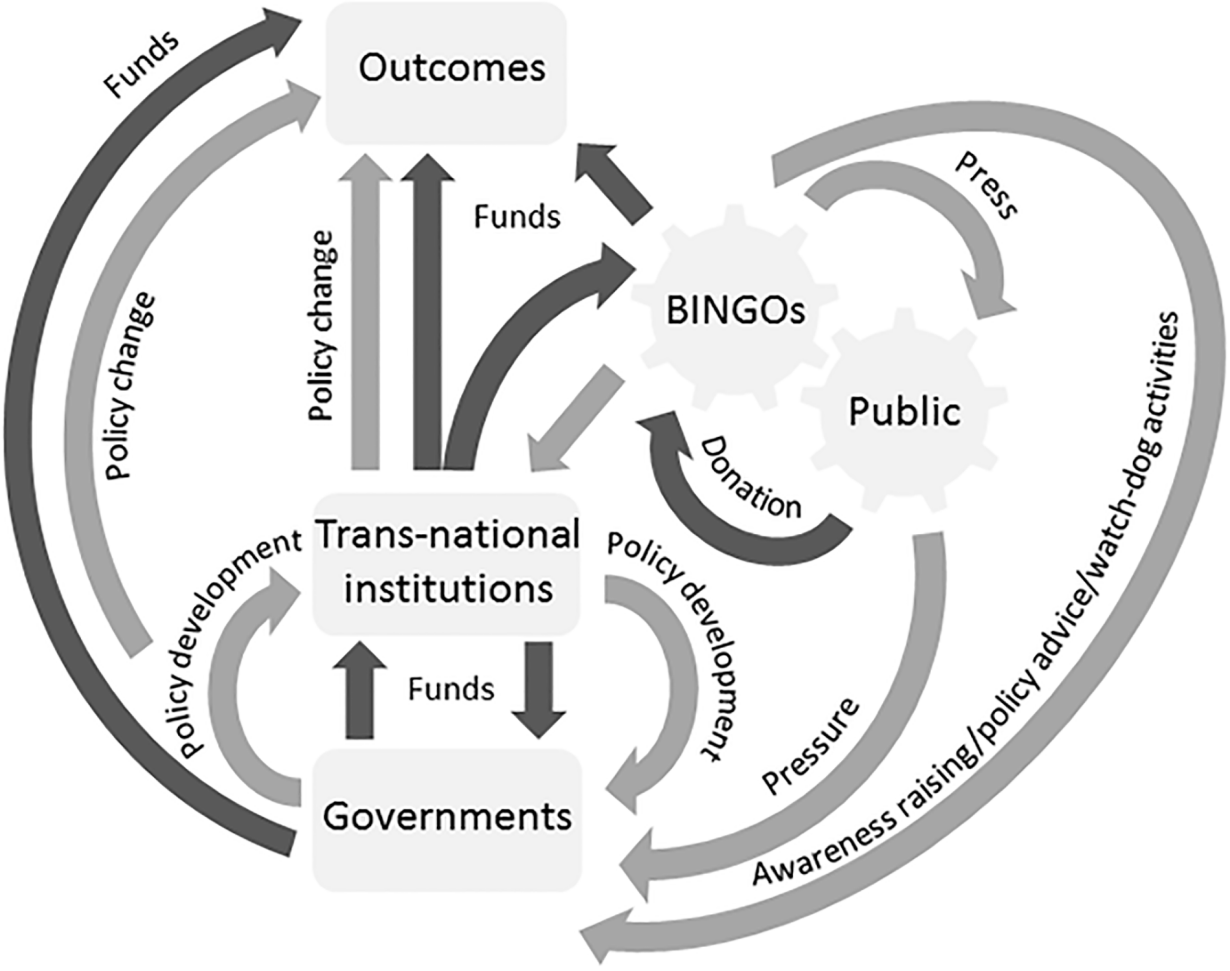
The interrelationship between the publicity that endangered species receive from BINGOs and donations/advocacy from the public is the ‘flywheel’ driving species conservation. A flow diagram representing how the movement of values (light grey arrows) and financial capital (dark grey arrows) between BINGOs, the public and conservation actors translates into outcomes for species. In this model, the association between media profiling by BINGOs and public support for endangered species (interlocking cogs) drives international investment in conservation programmes and the policy development that steers them.

In recent decades, Big International Non-Governmental Organisations (BINGOs) have come to have a powerful influence over the conservation agenda, including the flow of financial capital [1–2] (Fig 1). While BINGOs are not democratically accountable, their power and influence remains legitimised by public donations and popular advocacy for conservation issues [3]. The interrelationship between BINGOs and the public—which we refer to as the ‘flywheel’ of conservation governance—exposes conservation funding and policy decisions to the influence of prevailing cultural norms surrounding environmental issues in the general population, such that conservation priorities risk reflecting bias in popular attitudes, rather than the needs of the environment or society [4–5].

The interrelationship between popular perceptions of endangered species, and the publicity they receive from the conservation community is one prominent example of the ‘flywheel’ of conservation governance in action. BINGOs seek to address the biodiversity crisis [6–7], by fund raising through publicity campaigns, investing directly in conservation programmes, and influencing the conservation agendas of governments and trans-national institutions (Fig 1). Attractive ‘flagship’ species for example, are used to raise funds that support a variety of conservation initiatives, however the effectiveness of this approach to species conservation has been challenged on the grounds that it focuses public attention and conservation efforts on a limited number of taxa [8–9], prioritizes conservation spending according to popularity rather than need [10], and in some cases reduces the popular appeal of taxa that receive less publicity from the conservation community [11].

The ‘flywheel’ allows negative values associated with some species and the low cultural salience of many others, to influence financial investment in protection programmes, the level of publicity they receive, and ultimately their perceived importance by the public, scientists and the conservation community [4–5]. This has arguably led to large numbers of species threatened with extinction remaining largely absent from both popular concern and the conservation agenda [12–13]. Achieving the goals of the International Convention on Biological Diversity may also require raising the profile of lesser known endangered species in order to create clear political incentives and financial capital for protecting them, rather than allowing existing biases in popular advocacy to determine those species that are to be conserved for future generations. In order to do so, we must first identify those species threatened with extinction that have low cultural salience, and target media campaigning towards them [14]. To our knowledge, no empirical assessment of global popular interest in species, or its association with conservation spending, has ever been conducted across the vertebrates.

Conservation culturomics is an emerging field in the digital sciences that analyses the frequency of occurrence of words and phrases associated with conservation on the internet in order to quantify trends in the cultural salience of conservation issues [5, 14–15]. Trends in internet search traffic offer a similarly powerful tool by enabling biologists to ‘poll’ the global population’s interest in species of conservation concern [15]. By the end of 2016, 3.9 billion people (47% of the human population) had access to the internet [16], and in a world where 66% of the global population is predicted to be living in urban areas by 2050 [17], public support for species conservation will increasingly be influenced by online interactions with nature [18]. Data on internet search trends are already used to predict movement in financial markets [19] and disease outbreaks [20], as well as to assess public awareness of broader conservation issues [21–22], projects [23], and the cultural salience of species [24–25].

We quantified global popular interest in the world’s vertebrates using internet search trends on Google. Our analysis of this novel dataset reveals profound differences in popular interest towards threatened species from different vertebrate classes, demonstrates that this is associated with biased investment in the conservation of many endangered species, and identifies those endangered and critically endangered species that receive little popular interest.

## Results

The monthly web search interest in 36,873 species of vertebrate listed on the International Union for the Conservation of Nature’s (IUCN) Red List of threatened species was downloaded from the Google Trends web facility (see Methods for full details) between 14/10/2015 and 16/06/2016. For each species we downloaded trends in the monthly proportion of Google searches categorized by the engine as being associated with wildlife between 01/01/2004 and 31/12/2014. The search queries entered into the engine included the scientific and alternative common names of species given by the IUCN Red List in English, French and Spanish allowing us to extract information that reflected search interest from 37% of the world’s internet users [26]. While this approach may underestimate popular search interest in taxa endemic to regions where these languages are not commonly spoken, BINGO’s and their patrons are largely based in western hemisphere countries where they are. Each query was specified so that the data extracted reflected the total search interest for all alternative names (search terms), and was expressed relative to the maximum monthly web search interest of the African Lion (see S1 Fig), which we had previously identified as having the highest such value. As an example, the query “Anisotremus davidsonii" + "Grunt" + "Sargo" + "Xantic sargo" + "Burro piedrero" + "Sargo rayado", "Panthera leo" + "Lion" + "African Lion" + "Lion d’Afrique" + "León" provides the monthly search interest of all alternative names of the first species relative to the second (separated by a comma). This relative comparison was made necessary by Google’s presentation of web search interest data on a standardised scale between 0 and 100, with 100 being the maximum monthly search interest returned for any combination of search queries provided up to a limit of five queries in total. Species classified on the IUCN red list as ‘extinct’ or ‘extinct in the wild’ were not included in the analysis.

Using our approach we were able to quantify the average monthly web search interest of 1,598 vertebrate species. Google Trends did not provide data for the remaining 35,275 species due to insufficient search volume. The search interest of these species was therefore assumed to be 0, allowing us to conduct a two part analysis in which we first compared the average monthly web search interest of taxa that Google Trends provided data for, and second compared the probability of a species having received sufficiently high search volume for Google Trends to provide data (high versus low web search interest).

**Fig 2:**
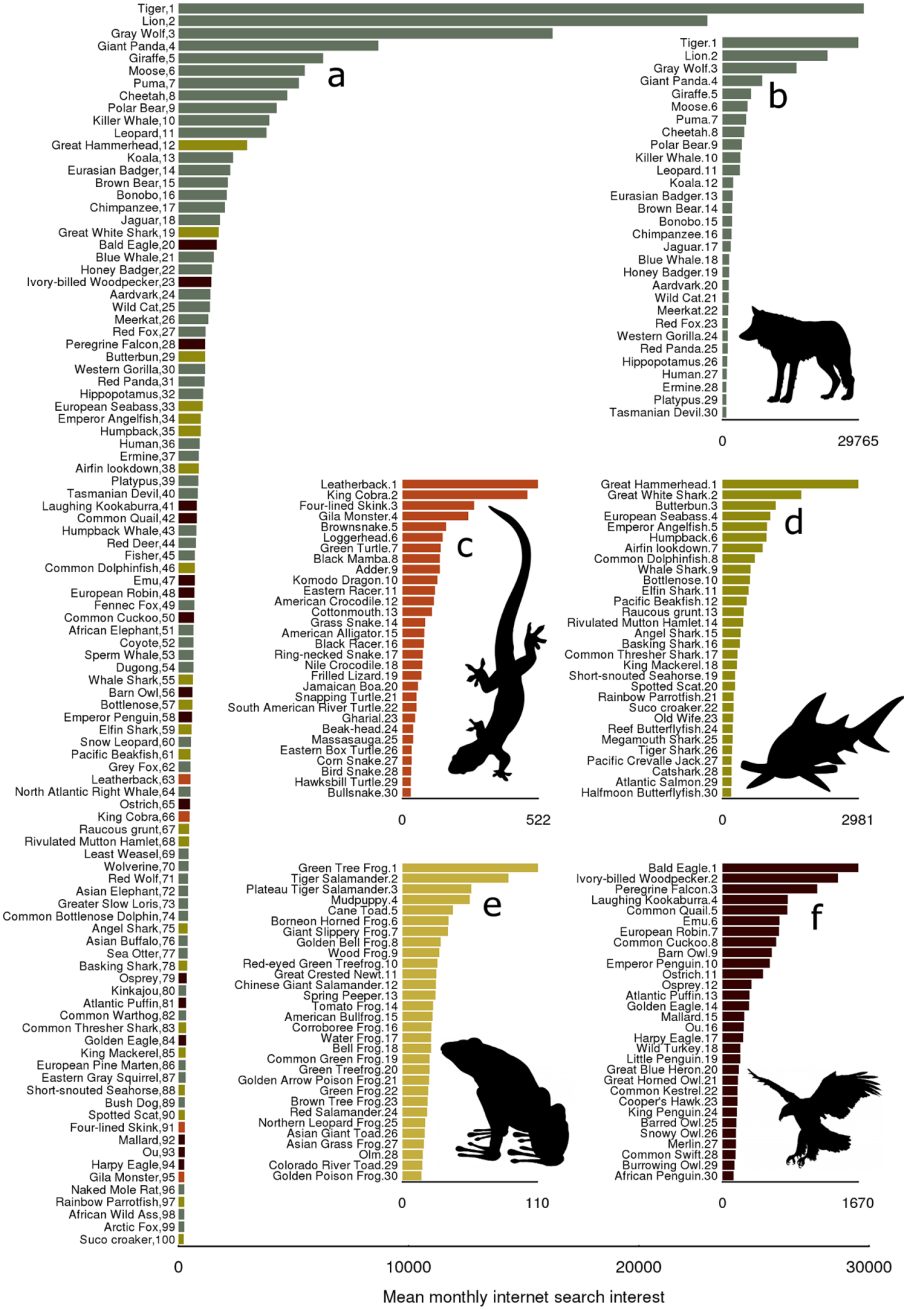
The most popular vertebrates on Google. **a**) Bar lengths are an index representing the average monthly web search interest in species between January 2004 and December 2014 on Google. Numbers represent the rank order of mean monthly web search interest. Web search interest was collated for the scientific, and all alternative English, French and Spanish common names as given by the IUCN red list. Inset, the top 30 most popular Mammals (**b**), Reptiles (**c**), Fish (**d**), Amphibians (**e**) and Birds (**f**).

The 100 most frequently searched for vertebrates on Google between 2004 and 2014 are given in Fig 2, along with the top 30 within each vertebrate class (scientific names and scores are given in S1–S6 Tables). The data revealed profound differences in web search interest between vertebrate classes. Mammals accounted for 57 of the 100 most searched for species, followed by fish (*n* = 22), birds (*n* = 17) and reptiles (*n* = 4), while no amphibian species were ranked in this group. Overwhelmingly, mammal species generally had higher web search interest than other classes of vertebrate with 6.6% ranked within the top 1000 most searched for taxa. 3.2% of fish species were among the top 1000, 2.0% of bird species, 1.6% of reptile species and 0.4% of amphibian species.

While those characteristics that make species more charismatic are increasingly well known [27], a qualitative assessment of the taxa in Fig 2 reveals that charisma is unlikely to be the only reason why people engage with species online. It is equally possible that several taxa score in the top 100, or top 30 within their class because they are common and frequently encountered in the real world (for example the European Robin, Eurasian Badger, Mallard and the Red Fox in Europe), are of commercial or recreational importance (for example the Emperor Angelfish in the reef aquarium trade; and the King Mackerel and European Seabass in sea angling), or are extremely rare and likely extinct (for example the Ou and the Ivory-billed Woodpecker). While the motivations that drive people to search for different species online are likely to be variable, and do not necessarily reflect concern for their welfare in the wild, the frequency of online interaction with a species is nonetheless a measure of its global cultural salience, and as such is representative of either indifference towards its future survival, or ignorance of its current existence.

**Fig 3:**
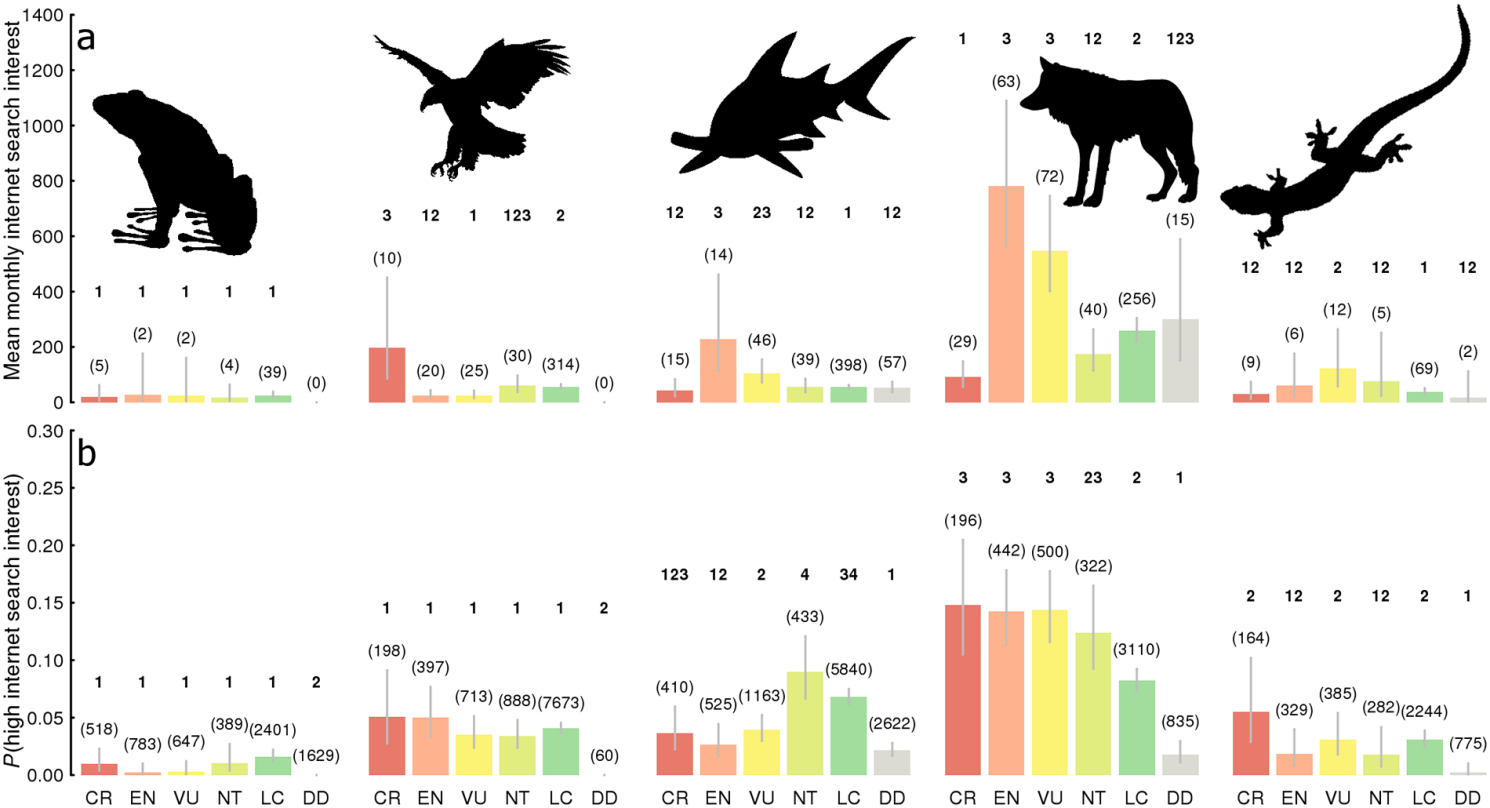
The web search interest of vertebrates compared by Class and Extinction risk (IUCN Red List Status). **a)** The average monthly web search interest for species with sufficiently high search volume for Google Trends to return data. **b**) The probability of a species having a sufficiently high search volume for Google Trends to return data. Bars represent means, error bars represent 95% confidence intervals. Graphs are derived from model predictions made using a negative binomial generalised linear model (**a**) and a binomial generalised linear model (**b**) where response = Class*Red List Status. Numbers in parenthesis represent the number of species in each Class*Red List Status combination. Numbers in bold define groups of Red List Status within each class that are significantly different at the 95% confidence level. Any two Red List Statuses within a Class that share a digit cannot be considered significantly different. Letters on the x axis of **b** denote each Red List Status classification (CR = Critically Endangered, EN = Endangered, VU = Vulnerable, NT = Near Threatened, LC = Least Concern, DD = Data Deficient).

Whether or not web search interest in a species reflected its extinction risk was dependent on the class of vertebrate under consideration. This was true both when comparing the probability of a species having a high web search interest (Binomial Generalised Linear Model, Class*Red List Status: *χ*^*2*^_20, 36843_ = 120.36, *p* < 0.001; Fig 3b), and when comparing the average monthly web search interest of those species that Google Trends provided data for (Negative Binomial GLM, Class*Red List Status: *χ*^*2*^_18, 1570_ = 80.07, *p* < 0.001; Fig 3a). Critically endangered, endangered and vulnerable mammals were significantly more likely to receive high web search interest compared to those of least concern (Fig 3b), and the average monthly web search interest of endangered and vulnerable mammals that Google Trends provided data for was significantly higher than those that were near threatened or of least concern (Fig 3a). Critically endangered bird species that Google Trends provided data for received significantly higher average monthly web search interest than species in other IUCN Red List categories (Fig 3a). Endangered species of fish also received significantly higher average monthly web search interest compared to other Red List categories (Fig 3a), however species that were near threatened or of least concern were significantly more likely to receive high web search interest in general (Fig 3b). Only vulnerable reptile species had a significantly higher monthly web search interest compared to those classified in other IUCN Red List categories (Fig 3a), while extinction risk had no impact on the probability of reptile species receiving high monthly search interest (Fig 3b). Web search interest in amphibians was unaffected by extinction risk (Fig 3). We conclude that popular interest in vertebrates is only higher for species threatened with extinction if they are a mammal or a bird, implying that a powerful class bias exists among the general population.

**Fig 4:**
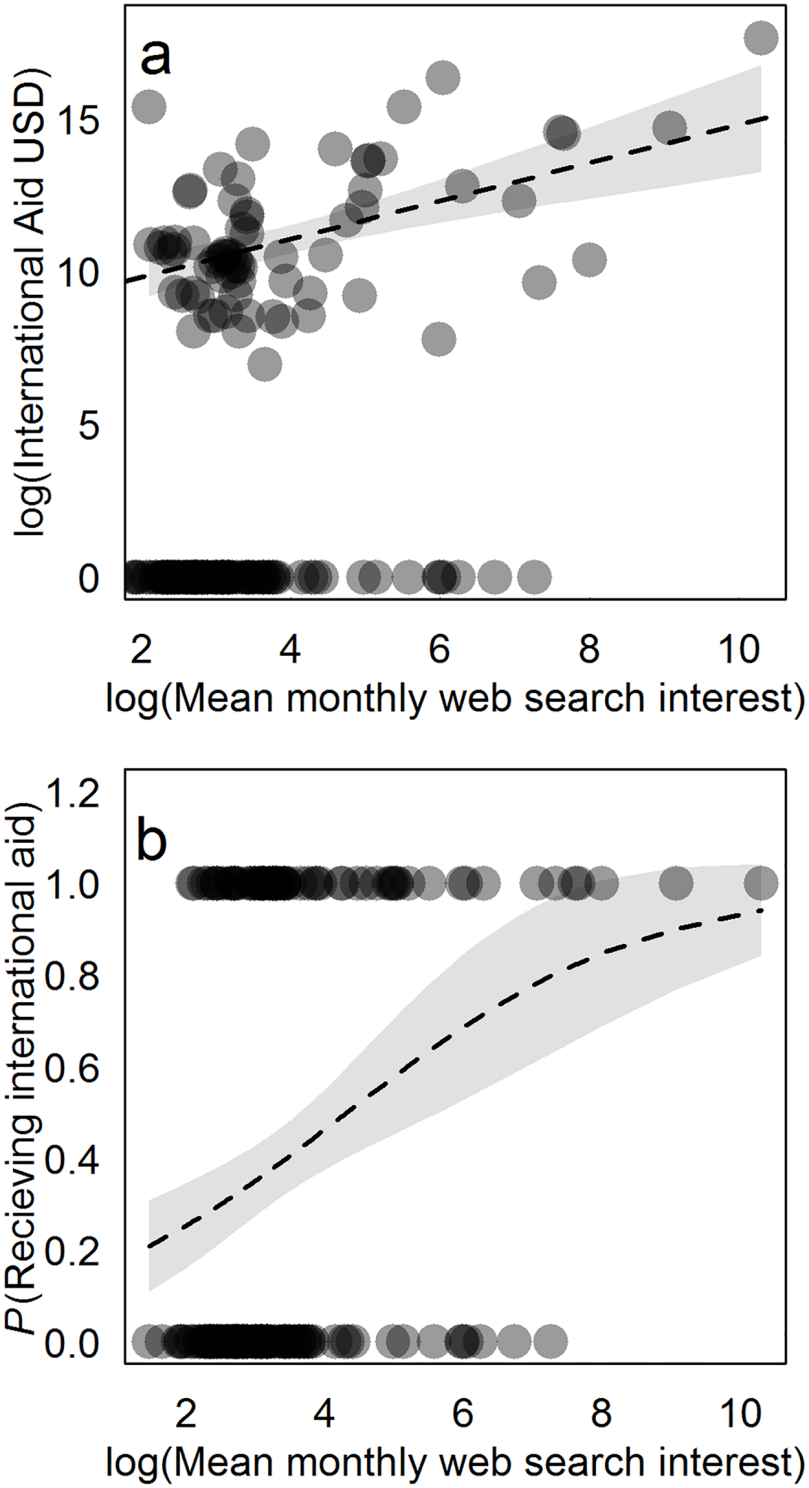
Higher online web search interest in endangered and critically endangered species of vertebrate is associated with greater financial investment in their conservation. **a)** The relationship between the average monthly web search interest in species between 2004 and 2014, and the amount of international aid invested in their protection (in US dollars) was quantified using a Gaussian GLM performed on log transformed (to reduce leverage) response and predictor variables. Species for which no financial investment was recorded (0 on the y axis) were not included in this analysis but were included in b. **b)** The relationship between the average monthly web search interest in species between 2004 and 2014, and the probability that they received international aid over the same time period, was quantified using a Binomial GLM performed on a log transformed (to reduce leverage) predictor variable. Values of 1 on the y axis indicate species that received aid, while values of 0 indicate those for which no aid was documented. The grey regions in both panels represent the 95% confidence intervals of the predicted relationships (broken lines). Data are presented for 173 species of critically endangered and endangered vertebrate that Google Trends returned web search interest data for. Aid data were downloaded from the Aiddata database (see Methods for details) and are given in S9 Table.

We were able to demonstrate that endangered and critically endangered species with low popular interest receive less financial capital invested in their conservation. To do so, we downloaded data on the total amount of international aid given to conservation programmes between 2004 and 2014 that was associated with the preservation of 173 endangered and critically endangered vertebrates that Google Trends returned data for (data extracted from the Aid data database on 08/09/2016). Where species were found to receive investment, higher mean monthly web search interest was significantly associated with increased financial capital invested in their conservation through international aid (Fig 4a, Gaussian GLM, *F*_(1,68)_ = 20.91, *p*<0.001). Species receiving high mean monthly web search interest were also significantly more likely to receive international aid (Fig 4b, Binomial GLM, *χ*^*2*^_(1,171)_ = 15.40, *p*<0.001). We are therefore confident that greater popular interest in vertebrate species as measured using web search frequency is associated with increased investment of financial capital in their conservation generally, although the variability around this relationship suggests that species with low web search interest can still receive substantial financial investment in some cases (for example see Corriea *et al*. [28]). While the counter argument that the popular profile of endangered species is elevated by high levels of conservation funding is equally valid, we would assert that BINGOs should show leadership in trying to raise the popular profile of lesser known threatened species and thereby generate incentives for investing in their protection, rather than allowing existing cultural biases among the human population to determine the prospects of these species surviving the 21^st^ century.

Our analysis identified 3,789 species of critically endangered and endangered vertebrate with low internet search interest (insufficient search volume for Google Trends to provide data), including 546 species of mammal, 565 species of bird, 906 species of fish, 478 species of reptile and 1294 species of amphibian (S7 Table). These species are at high risk of extinction yet receive low popular interest. Our results suggest that incentives for expending resources on raising their popular profile, lobbying for their protection, and investing directly in their conservation, are also low. Indeed, the populations of 2,671 of these species (71%) are considered to be declining by the IUCN, comparable with the 126 out of 173 critically endangered and endangered vertebrates that we identified as receiving high internet search interest (73%).

## Discussion

Our results empirically demonstrate that popular interest is associated with financial investments in the conservation of vertebrate species facing extinction around the world. Incentives to invest financial capital in the conservation of the majority of critically endangered and endangered vertebrate species are likely reduced by their low popularity, while a minority of popular species are more likely to receive financial investment. Previous studies based on smaller sample sizes have also shown that the frequency of species reintroduction programmes is taxonomically bias [4], and the frequency of scientific articles investigating species reflects their web presence [5], this latter finding demonstrating that popular interest influences the priorities of scientists in the same way that we show for conservation actors here.

We propose that more can be done to champion the cause of species with low popular interest by BINGOs and the global media, helping to raise their popular appeal and thereby create clear political and financial incentives to safeguard their existence. Commercial marketing approaches have recently been proposed as a novel tool for raising the popular profile of lesser known endangered species [29–31]. While this approach can improve awareness of and hence investment in appealing taxa that have been overlooked, it remains equally vulnerable to the cultural biases highlighted by our analysis. This is because marketing is invariably focused on appealing to existing consumer values, rather than on cultivating different cultural norms. The challenge for species conservation lies in identifying how to engender in people positive values associated with lesser known endangered species that have not been the traditional focus of BINGOs precisely because they are perceived to have low consumer appeal [32–34]. Successfully doing so will improve the prospects of such species receiving conservation investment, but requires a merging of the fields of conservation marketing, psychology, biology and practice [35]. Without ignoring the plight of other phyla, BINGOs should urgently seek to raise the popular profile of lesser known endangered and critically endangered species to provide incentives for their conservation.

## Materials and methods

### Data acquisition

The Google Trends search engine allows internet users to extract trends in internet searches entered into Google. The data returned by Google Trends are unaffected by personalisation algorithms, since they represent the frequency of internet searches for terms of interest through the Google search engine, rather than the salience of these terms on the digital corpus. The data can be filtered by time interval, country, and category (e.g. wildlife). The category filter enabled us to ensure that all data collected during our study reflected internet searches associated only with wildlife, eliminating sources of bias due to homonyms that exist in the rest of human culture (so that the term ‘Jaguar’, for example, was not conflated with the car manufacturer) [15]. Data can be requested for up to five search queries per request, and at the time that data collection was conducted, Google Trends limited each query to 30 search terms. The data returned represents the monthly search volume for the given query as the proportion of total search traffic through Google. For any combination of up to five search queries, the data is standardised on a scale of between 0 and 100, where 100 represents the maximum search interest received for any of the five queries in any month of the specified time period. For further information on how Google Trends data are standardised see the Google Trends help centre at https://support.google.com/trends/.

We downloaded IUCN Red List data for all vertebrate species (*n* = 37,355) on 28/02/2014 including classifications of Class, Biome, IUCN Red List Status, Population Trend, Scientific names and all provided alternative common names given in English, French and Spanish. Our goal was to quantify global web search interest in these species relative to one another, which given Google Trends’ standardisation of the data, required that the search interest of each was expressed relative to the species with the highest monthly search interest over the specified time period. We began by identifying those taxa that received sufficiently high search volume for Google Trends to return data. Where the search volume in a given query was not sufficiently high for Google Trends to return data, its monthly search interest was assumed to be 0. The search query for each taxon consisted of the scientific and common names of that species given in English, Spanish and French on the IUCN red list so that the query "Panthera leo" + "Lion" + "African Lion" + "Lion d’Afrique" + "León" returned the sum of the search interest per time interval for the exact phrase “Panthera leo” OR the exact phrase “Lion” and so on.

In cases where the number of common names given for a species by the IUCN exceeded Google Trends limit of 30 search terms, we removed common names from each of the alternative languages in turn so that the search query complied with this limit. Names were removed from each language sequentially (one Spanish, one French, one English) from the last given name in each language.

Google Trends did not recognise the language of some of the alternative common names provided by the IUCN for 20 species. These unrecognised names were removed from the search queries provided for each species in order that internet search trends could be extracted. No more than three common names were removed in each individual case, and there was only one case in which removing these common names resulted in the search query for a species containing only its scientific name.

The Google Trends algorithm only had sufficient data to return results for 1749 species (5% of 37,555). We quantified the search interest of these 1,749 species relative to one another, and scaled them relative to the highest monthly search interest for the Lion, which we had previously identified as having the highest such value (described in S1 Fig). Species that were classified by the IUCN Red List as ‘Extinct’ or ‘Extinct in the Wild’ were omitted from the analysis (*n* = 341 taxa). This left 1,739 taxa that Google returned data for and 35,275 taxa with insufficient search volume for Google Trends to return data.

There were 141 cases of taxa with identical common names receiving similar internet search interest (identified as those with shared common names whose monthly web search interest was highly correlated [Pearson’s correlation>0.90], see S8 Table). While it is likely that the search interest for at least some of these taxa is inflated by one or two species that were intentionally searched for, there is no reliable method of discriminating target species from incidental species. All such species were therefore removed from the analysis to avoid inflating the leverage of popular species that share common names with other species. The analysis presented was therefore performed on 36, 873 taxa, 1,598 of which Google Trends returned data for, and 35,275 with insufficient search volume for Google Trends to return data.

International aid data (in US dollars) was downloaded from the Aid data database (www.aiddata.org) on 08/09/2016 for each of 173 critically endangered and endangered vertebrate species from both developed and developing countries that Google Trends returned data for (S9 Table). Keyword searches were performed using the first given vernacular name of each species on the IUCN red list, or scientific name where none was available. The data output was filtered by time (January 2004 to December 2014), sector (410: General Environmental Protection), activity (41030.03: Species Protection) and the default source setting (‘AidData’ including ‘Donor provided’ and aid reported via the ‘OECD Common Reporting Standard’). Data were not disaggregated for NGO spending since it was our thesis that the interrelationship between NGOs and the public influences the flow of both ideas and capital through the global conservation system (Fig 1), hence it affects decisions about the spending priorities of transnational institutions and governments as well as those of the NGOs themselves.

## Analysis

Four hypotheses were tested during the analysis. In the first we tested whether the relationship between mean monthly web search interest and the extinction risk (IUCN Red List status) of vertebrates was dependent on the taxonomic class to which they belonged (mean monthly web search interest ~ Extinction Risk*Class) using Negative Binomial generalised linear regression (Fig 3a). In the second we tested whether the relationship between the probability of receiving high internet search interest (those that Google Trends returned data for as opposed to having insufficient search volume) and the extinction risk (IUCN red list status) of vertebrates was dependent on the taxonomic class to which they belonged using a Binomial generalised linear model to account for the probabilistic nature of the response distribution (Fig 3b). The interaction between Class and Extinction Risk was significant in both analyses, therefore the IUCN Red List categories were grouped according to those that could be considered significantly different at the 95% confidence level within each vertebrate class using pairwise comparisons performed in the CRAN package lsmeans [36].

In the third and fourth analyses we tested whether popular interest in species as measured using internet search trends was associated with international aid given to their conservation. While we acknowledge that in reality the relationship between these two variables is reciprocal in nature, our assertion is that if a relationship can be statistically demonstrated, then it is reasonable to suggest that raising the popular interest shown towards species at risk of extinction could provide greater incentives to invest in their conservation. Similar to the previous analysis we conducted two tests, one in which we asked whether species in receipt of more popular interest also received more international aid for their protection, and a second in which we asked whether species in receipt of more popular interest had a greater probability of receiving international aid. The first relationship was tested using a Gaussian generalised linear model performed on log transformed response and predictor variables. While the relationship between the non-transformed variables was linear, the log transformation of both was necessary to reduce leverage caused by logarithmic distributions. The second relationship was tested using a Binomial generalised linear model to account for the probabilistic nature of the response distribution. The predictor variable was again log transformed in order to reduce leverage.

## Supporting information

S1 Fig Google Trends data acquisition and standardisation. (PDF)

S1 Table The top 100 most Googled vertebrates in the world. Data corresponding to Fig 2a.
(PDF)

S2 Table The top 100 most Googled mammals in the world. Data corresponding to Fig 2b.
(PDF)

S3 Table The top 100 most Googled reptiles in the world. Data corresponding to Fig 2c.
(PDF)

S4 Table The top 100 most Google fish in the world. Data corresponding to Fig 2d.
(PDF)

S5 Table The top 52 most Googled amphibians in the world. Data corresponding to Fig 2e.
(PDF)

S6 Table The top 100 most Googled birds in the world. Data corresponding to Fig 2f.
(PDF)

S7 Table Species of endangered and critically endangered vertebrate that receive low web search interest. (PDF)

S8 Table Species that were omitted from the analysis due to containing one or more identical common names and having highly correlated monthly web search interest (Pearson’s correlation >0.90). (PDF)

S9 Table Raw data used in the international aid data analysis (Fig 4). (PDF)
