# Correction: Popular interest in vertebrates does not reflect extinction risk and is associated with bias in conservation investment

**DOI:** 10.1371/journal.pone.0212101

**Published:** 2019-02-06

**Authors:** Thomas Davies, Andrew Cowley, Jon Bennie, Catherine Leyshon, Richard Inger, Hazel Carter, Beth Robinson, James P. Duffy, Stefano Casalegno, Gwladys Lambert, Kevin Gaston

The middle initial of the eighth author is incorrectly omitted. The eighth author’s correct name is: James P. Duffy.
