# Supplementary material for: Popular interest in vertebrates does not reflect extinction risk and is associated with bias in conservation investment
Source: PLoS One. 2018 Sep 26;13(9):e0203694. doi: 10.1371/journal.pone.0203694 (PMC6157853; doi:10.1371/journal.pone.0203694)
Supplement: S1 Fig — (PDF) [file pone.0203694.s001.pdf]

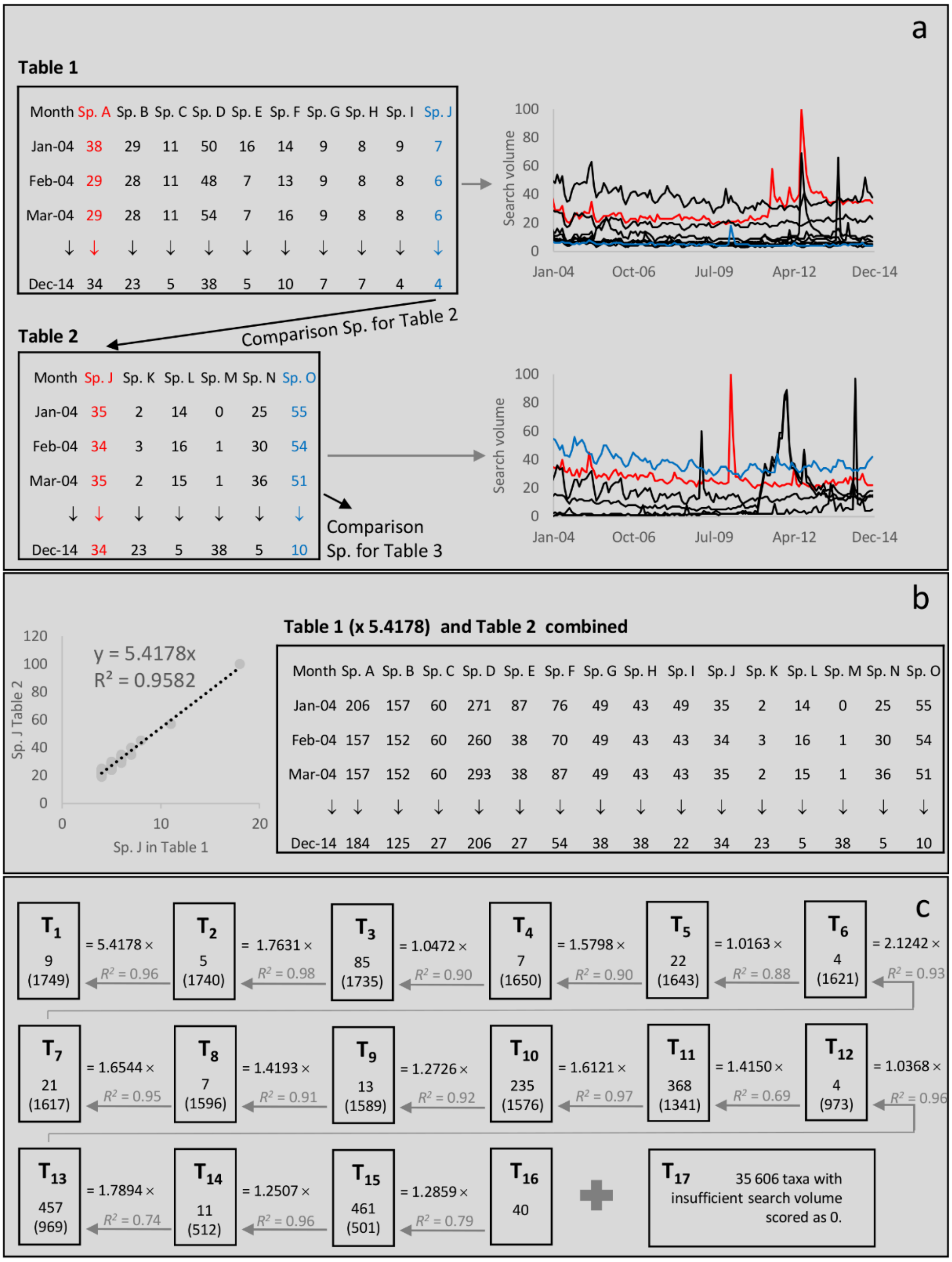

Figure S1. Google Trends data acquisition and standardisation. a) Monthly search interest was scaled for all species relative to a reference species (Sp. A) and extracted for those where mean monthly search interest was >=5 or the maximum monthly search interest was greater than 50 (Table 1). A second species in Table 1 (Sp. J) was then used as the reference species against which to scale the remaining taxa in a new submission of queries to Google Trends. Again those taxa with mean monthly search interest >5 or maximum monthly search interest >50 were extracted. This process was repeated until data had been extracted from all 1749 species that Google Trends returned for. **b)** The coefficient of the 0 intercept relationship between monthly search interest in the reference species that bridged each sequential pair of tables was used as the scaling factor by which to multiply the first (in this example Table 1) to make values relative to the second (in this example Table 2). **c)** The scaling factors (0 intercept coefficients) and associated R^2 values used to scale pairs of Tables sequentially. Numbers in boxes represent the number of taxa extracted in each Table, numbers in parenthesis represent the cumulative total number of taxa extracted.
