# Supplementary material for: Popular interest in vertebrates does not reflect extinction risk and is associated with bias in conservation investment
Source: PLoS One. 2018 Sep 26;13(9):e0203694. doi: 10.1371/journal.pone.0203694 (PMC6157853; doi:10.1371/journal.pone.0203694)
Supplement: S1 Table — Data corresponding to Fig 2a. (PDF) [file pone.0203694.s002.pdf]

S1 Table. The top 100 most Googled vertebrates in the world. Data corresponding to Figure 2a. The alternative common names that were the terms of the search query for each species (along with the scientific name), are given in the far right column. These are the alternative common names in English, French and Spanish as given on the IUCN red list of threatened species.

Rank	Scientific name	Common name	Average monthly web search interest	All common names
1	*Panthera tigris*	Tiger	29765.26	Tiger, Tigre
2	*Panthera leo*	Lion	22977.01	Lion, African Lion, Lion d'Afrique, León
3	*Canis lupus*	Gray Wolf	16248.67	Gray Wolf, Tundra Wolf, Arctic Wolf, Grey Wolf, Mexican Wolf, Plains Wolf, Timber Wolf, Common Wolf, Wolf, Loup, Loup Gris, Loup Vulgaire, Lobo, Lobo
4	*Ailuropoda melanoleuca*	Giant Panda	8681.54	Giant Panda, Panda, Panda géant, Panda Gigante
5	*Giraffa camelopardalis*	Giraffe	6278.98	Giraffe
6	*Alces alces*	Moose	5489.53	Moose, Eurasian Elk, Elk, European Elk, Eurasian Moose, Siberian Elk, Élan, Alce
7	*Puma concolor*	Puma	5236.48	Puma, Mountain Lion, Cougar, Red Tiger, Deer Tiger, León Americano, León Bayo, León Colorado, León De Montaña, Mitzli, Onza Bermeja
8	*Acinonyx jubatus*	Cheetah	4727.21	Cheetah, Hunting Leopard, Guépard, Chita, Guepardo
9	*Ursus maritimus*	Polar Bear	4259.88	Polar Bear, Ours polaire, Ours blanc, Oso Polar
10	*Orcinus orca*	Killer Whale	3951.28	Killer Whale, Orca, Orque, Epaulard, Espadarte, Espadarte, Orca
11	*Panthera pardus*	Leopard	3818.59	Leopard, Panthère, Léopard, Leopardo, Pantera
12	*Sphyrna mokarran*	Great Hammerhead	2981.86	Great Hammerhead, Hammerhead Shark, Squat-headed Hammerhead Shark, Grand Requin-marteau, Marieau Millet, Poisson Pantouflier, Sorosena, Cornuda, El Tiburon, Guardia Civil, Pez Martillo, Tiburon
13	*Phascolarctos cinereus*	Koala	2377.63	Koala
14	*Meles meles*	Eurasian Badger	2249.35	Eurasian Badger, Badger, BLAIREAU EUROPÉEN, Blaireau Européen, Tejón, TEJÓN
15	*Ursus arctos*	Brown Bear	2145.13	Brown Bear, Mexican Grizzly Bear, Grizzly Bear, Ours brun, Oso Pardo
16	*Pan paniscus*	Bonobo	2105.62	Bonobo, Pygmy Chimpanzee, Gracile Chimpanzee, Dwarf Chimpanzee, Chimpanzé Nain, Chimpanzé pygmée, Chimpancé Pigmeo
17	*Pan troglodytes*	Chimpanzee	2027.21	Chimpanzee, Robust Chimpanzee, Common Chimpanzee, Chimpanzé, Chimpancé
18	*Panthera onca*	Jaguar	1817.09	Jaguar, Tigre Real, Tigre Americano, Otorongo, Yaguar, Yaguarete
19	*Carcharodon carcharias*	Great White Shark	1736.18	Great White Shark
20	*Haliaeetus leucocephalus*	Bald Eagle	1670.95	Bald Eagle
21	*Balaenoptera musculus*	Blue Whale	1526.06	Blue Whale, Sibbold's Rorqual, Sulphur-bottom Whale, Pygmy Blue Whale, Baleinoptère bleue, Baleine bleue, Baleine d'ostende, Rorqual à ventre cannelé, Rorqual Bleu, Rorqual de Sibbold, Ballena Azul, Rorcual Azul
22	*Mellivora capensis*	Honey Badger	1443.16	Honey Badger
23	*Campephilus principalis*	Ivory-billed Woodpecker	1421.69	Ivory-billed Woodpecker
24	*Orycteropus afer*	Aardvark	1394.34	Aardvark, Antbear, Oryctérope

S1 Table continued

Rank	Scientific name	Common name	Average monthly web search interest	All common names
25	*Felis silvestris*	Wild Cat	1376.77	Wild Cat, Wildcat, Chat Sauvage, Chat Orné, Gato Montés, Gato Silvestre
26	*Suricata suricatta*	Meerkat	1307.15	Meerkat, Slender-tailed Meerkat, Suricate
27	*Vulpes vulpes*	Red Fox	1174.18	Red Fox, Silver Fox, Cross Fox, RENARD, Renard Roux, ZORRO, Zorro Rojo
28	*Falco peregrinus*	Peregrine Falcon	1166.65	Peregrine Falcon, Peregrine, Faucon pèlerin
29	*Chaetodon capistratus*	Butterbun	1166.02	Butterbun, Butterfly, Four-eye Butterflyfish, Foureye Butterflyfish, Foureye Butterflyfish, Four-eyed butteflyfish, Kete, School Mistress, Marguerite, Parché, Isabelita, Isabelita blanca, Kete, Mariposa, Mariposa ocelada, Parche, Parche ocelado
30	*Gorilla gorilla*	Western Gorilla	1165.4	Western Gorilla, Lowland Gorilla, Gorille, Gorila
31	*Ailurus fulgens*	Red Panda	1139.68	Red Panda, Lesser Panda, Red Cat-bear, Panda Éclatant, Petit Panda, Panda Chico, Panda Rojo
32	*Hippopotamus amphibius*	Hippopotamus	1069.43	Hippopotamus, Large Hippo, Common Hippopotamus, Hippopotame, Hipopótamo Anfibio
33	*Dicentrarchus labrax*	European Seabass	1055	European Seabass, Common Bass, European Bass, Bass, King Of The Mullets, Sea Dace, Sea Perch, White Mullet, White Salmon, Capemouth, Loubas Negre, Bar, Bar Commun, Bar Européen, Bar Franc, Bog, Brigue, Drelique, Gutgareo, Loubine, Loup, Loupassou, Loup de Mer, Lubin, Luvassu, Pigne, Baieta, Baila, Cherne, Llop, Llubina, Lubaro, Lubina, Mero, Pintat, Robaliza, Robalo
34	*Pomacanthus imperator*	Emperor Angelfish	977.23	Emperor Angelfish, Imperial Angelfish, Angelfish, Emperor, Ange de mer impérial, Holacanthe empereur, Poisson ange impérial
35	*Chanodichthys dabryi*	Humpback	963.43	Humpback
36	*Homo sapiens*	Human	922.66	Human
37	*Mustela erminea*	Ermine	892.55	Ermine, Stoat, Short-tailed Weasel, Hermine, Armiño
38	*Selene brevoortii*	Airfin lookdown	882.52	Airfin lookdown, Hairfin lookdown, Mexican lookdown, Mexican lookdown (FB), Musso corcovade, Antena, Cara caballa, Carita, Carita jorobada, Espejuelo, Jorobado, Jorobado antena, Jorobado mexicano, Radio
39	*Ornithorhynchus anatinus*	Platypus	859.31	Platypus, Duck-billed Platypus, Ornithorynque
40	*Sarcophilus harrisii*	Tasmanian Devil	837.98	Tasmanian Devil, Diable De Tasmanie
41	*Dacelo novaeguineae*	Laughing Kookaburra	802.86	Laughing Kookaburra, Kookaburra
42	*Coturnix coturnix*	Common Quail	798.47	Common Quail, Quail, Caille des blés
43	*Megaptera novaeangliae*	Humpback Whale	768.36	Humpback Whale, Hump Whale, Hunchbacked Whale, Bunch, Mégaptère, Baleine à bosse, Baleine à taquet, Jubarte, Rorqual à bosse, Rorqual du Cap, Ballena Jorobada, Gubarte, Jorobada, Rorcual Jorobado
44	*Cervus elaphus*	Red Deer	760.21	Red Deer, Bactrian Wapiti, Bokharan Deer, Bukhara Red Deer, Elk, Bactrian Deer, Wapiti, Bactrian Red Deer, Cerf De Bactriane, Cerf Du Turkestan, Cerf Élaphe, Cerf Élaphe Du Turkestan, Cerf Rouge Du Turkestan, Ciervo, Ciervo Bactriano, Ciervo Rojo
45	*Martes pennanti*	Fisher	720.69	Fisher
46	*Coryphaena hippurus*	Common Dolphinfish	707.52	Common Dolphinfish, Dolphinfish, Dolphin Fish, Dorado, Green Dolphin, Mahimahi, Mahi-mahi, Mahi Mahi, Common Dolphin Fish, Clic, Coryphène Commune, Dauphin, Dorade, Dorade Coryphène, Dorado Común, Dorado Delfin, Lampuga, Llampuga

S1 Table continued

Rank	Scientific name	Common name	Average monthly web search interest	All common names
47	*Dromaius novaehollandiae*	Emu	704.38	Emu
48	*Erithacus rubecula*	European Robin	696.85	European Robin, Robin, Rougegorge
49	*Vulpes zerda*	Fennec Fox	694.97	Fennec Fox, Fennec
50	*Cuculus canorus*	Common Cuckoo	662.36	Common Cuckoo, European Cuckoo, Cuckoo, Coucou gris
51	*Loxodonta africana*	African Elephant	655.46	African Elephant, Éléphant d'Afrique, Éléphant Africain, Elefante Africano
52	*Canis latrans*	Coyote	653.58	Coyote, American Jackal, Brush Wolf, Prairie Wolf
53	*Physeter macrocephalus*	Sperm Whale	651.07	Sperm Whale, Spermacet Whale, Cachelot, Pot Whale, Cachalot, Ballena Esperma, Ballena Esperma, Cachalote, Cachalote
54	*Dugong dugon*	Dugong	649.19	Dugong, Sea Cow, Dugon
55	*Rhincodon typus*	Whale Shark	620.33	Whale Shark, Requin Baleine, Tiburón Ballena
56	*Tyto alba*	Barn Owl	606.53	Barn Owl, Chouette effraie
57	*Mormyrus kannume*	Bottlenose	603.4	Bottlenose
58	*Aptenodytes forsteri*	Emperor Penguin	585.83	Emperor Penguin
59	*Mitsukurina owstoni*	Elfin Shark	569.52	Elfin Shark, Goblin Shark, Requin Lutin, Tiburones Duende
60	*Panthera uncia*	Snow Leopard	541.3	Snow Leopard, Ounce, Once, Irbis, Léopard des neiges, Panthère des neiges, Leopardo Nival, Pantera de la Nieves
61	*Oplegnathus insignis*	Pacific Beakfish	531.89	Pacific Beakfish, Loreta, Loro, San Pedro, Tigris
62	*Urocyon cinereoargenteus*	Grey Fox	523.11	Grey Fox, Tree Fox, Gray Fox, Gato De Monte, Gato Cervan, Zorro, Zorro Gris, Zorro Plateado
63	*Dermochelys coriacea*	Leatherback	522.48	Leatherback, Leatherback Sea Turtle, Leathery Turtle, Luth, Trunkback Turtle, Trunk Turtle, Coffin-back, Tortue luth, Cardon, Baula, Canal, Dorso de Cuero, Galapagos, Siete Lomos, Siete Quillas, Tinglada, Tinglar, Tora, Tortuga Caná, Tortuga Laud
64	*Eubalaena glacialis*	North Atlantic Right Whale	516.21	North Atlantic Right Whale, Northern Right Whale, Right Whale, Black Right Whale, Baleine De Biscaye, Baleine Des Basques, Ballena, Ballena Franca Del Norte, Ballenga
65	*Struthio camelus*	Ostrich	501.79	Ostrich, Common Ostrich, Autruche
66	*Ophiophagus hannah*	King Cobra	481.09	King Cobra, Hamadryad
67	*Haemulopsis leuciscus*	Raucous grunt	463.52	Raucous grunt, White grunt, Boquimorado chato, Negro, Ronco roncacho, Ronco ruco
68	*Alphestes multiguttatus*	Rivulated Mutton Hamlet	459.76	Rivulated Mutton Hamlet, Rock Bass, Sea Bass, Pacific Guaseta, Vareche Veine, Varech Veine, Cherne, Cabrilla, Colorado, Companero De Mero, Guaseta Rayada, Guaseta Rayado, Guato, Mero
69	*Mustela nivalis*	Least Weasel	451.61	Least Weasel, Weasel, Belette d'Europe, Comadreja
70	*Gulo gulo*	Wolverine	436.55	Wolverine, GLOUTON, Glouton, Glotón, GLOTÓN
71	*Canis rufus*	Red Wolf	423.38	Red Wolf
72	*Elephas maximus*	Asian Elephant	417.48	Asian Elephant, Indian Elephant, Eléphant D'Asie, Eléphant D'Inde, Elefante Asiático

S1Table continued

Rank	Scientific name	Common name	Average monthly web search interest	All common names
73	*Nycticebus coucang*	Greater Slow Loris	416.88	Greater Slow Loris, Sunda Slow Loris, Slow Loris, Loris Lent, Loris Lento
74	*Tursiops truncatus*	Common Bottlenose Dolphin	411.49	Common Bottlenose Dolphin, Bottlenose Dolphin, Bottle-nosed Dolphin, Bottlenosed Dolphin, Grand Dauphin, dauphin souffleur, grand dauphin, Souffleur, Tursiops, Delfín Mular, Pez Mular, Tursión, Tursión
75	*Squatina squatina*	Angel Shark	400.17	Angel Shark, L'ange, Ange De Mer, Angel, Antjou, Bourgeois, Bourget, L'anelot, Ange, Martrame, Mordacle, Squatine Occelee, Angelote, Mermejuela, Pardon, Pez Angel
76	*Bubalus arnee*	Asian Buffalo	397.11	Asian Buffalo, Asiatic Buffalo, Indian Buffalo, Indian Water Buffalo, Water Buffalo, Wild Asian Buffalo, Wild Water Buffalo, Buffle D'Eau, Buffle De L'Inde, Bufalo Arni
77	*Enhydra lutris*	Sea Otter	395.91	Sea Otter, Loutre De Mer, Nutria Del Kamtchatka, Nutria Marina
78	*Cetorhinus maximus*	Basking Shark	387.86	Basking Shark, Pelerin, Peregrino
79	*Pandion haliaetus*	Osprey	355.33	Osprey, Balbuzard pêcheur
80	*Potos flavus*	Kinkajou	334.02	Kinkajou, Mico De Noche, Cusu, Martilla, Chosna, Mico León, Mono Michi, Perro De Monte
81	*Fratercula arctica*	Atlantic Puffin	333.66	Atlantic Puffin, Puffin
82	*Phacochoerus africanus*	Common Warthog	332.95	Common Warthog, Warthog, Eritrean Warthog, Phacochère Commun
83	*Alopias vulpinus*	Common Thresher Shark	330.83	Common Thresher Shark, Renard, Zorro
84	*Aquila chrysaetos*	Golden Eagle	326.06	Golden Eagle, Aigle royal
85	*Scomberomorus cavalla*	King Mackerel	321.51	King Mackerel, Kingfish, Maquereau, Thazard Barré, Carite Sierra, Carite, Carite Lucio, Carito, Carito Lucio, Peto, Rey, Serrucho, Sierra
86	*Martes martes*	European Pine Marten	314.3	European Pine Marten, Pine Marten, Pine Martin, European Pine Martin, Martre Des Pins, MARTRE DES PINS, Marta, MARTA
87	*Sciurus carolinensis*	Eastern Gray Squirrel	305.58	Eastern Gray Squirrel, Gray Squirrel, Grey Squirrel
88	*Hippocampus hippocampus*	Short-snouted Seahorse	281.28	Short-snouted Seahorse, Short Snouted Seahorse, Sea Horse, Hippocampe, Cheval de Mer, Hippocampe à Museau Court, Caballito de Mar, Caballo Marino, Cabalo de Mar
89	*Speothos venaticus*	Bush Dog	280.81	Bush Dog, Vinegar Dog, Savannah Dog, Chien Des Buissons, Zorro, Perrito Venadero, Cachorro Vinagre, Guanfando, Pero Selvático, Perrito de Monte , Perro de Agua, Perro de la Selva, Perro De Monte, Perro Grullero , Perro Vinagre , Umba, Zorrito Vinagre, Zorro Pitoco , Zorro Vinagre
90	*Scatophagus argus*	Spotted Scat	277.15	Spotted Scat, Spotted Butt, Butter Fish, Common Scat, Leopard Scat, Scat, Butterfish, Spotted Butterfish, Spotted Butter Fish, Spotted Scad, Argus Fish, Pavillon Tacheté, Pingo Manchado
91	*Plestiodon tetragrammus*	Four-lined Skink	275.45	Four-lined Skink, Escorpion De Cola Azul, Lince
92	*Anas platyrhynchos*	Mallard	268.05	Mallard, Northern Mallard, Common Mallard, Canard colvert
93	*Psittirostra psittacea*	Ou	264.78	Ou, 'O'u
94	*Harpia harpyja*	Harpy Eagle	254.4	Harpy Eagle, American Harpy Eagle, Aguila Harpía, Aguila Arpía, Arpía, Arpía Mayor, Harpía
95	*Heloderma suspectum*	Gila Monster	253.26	Gila Monster, Lézard perlé, Monstre de gila, Monstruo de Gila
96	*Heterocephalus glaber*	Naked Mole Rat	250.54	Naked Mole Rat

S1 Table continued

Rank	Scientific name	Common name	Average monthly web search interest	All common names
97	*Scarus guacamaia*	Rainbow Parrotfish	249.15	Rainbow Parrotfish, Blue Rainbow, Rainbow, Perroquet arc-en-ciel, Guacamaia, Guacamaya, Loro Guacamayo
98	*Equus africanus*	African Wild Ass	248.02	African Wild Ass, Ass, African Ass, Ane sauvage d'Afrique, Âne Sauvage D'Afrique, Asno Salvaje de Africa
99	*Alopex lagopus*	Arctic Fox	242.86	Arctic Fox, Polar Fox, Renard Polaire, Isatis, Reynard Polaire, Zorro Ártico
100	*Paralonchurus dumerilii*	Suco croaker	239.51	Suco croaker, Bourrugue suco, Barcelona, Lambe suco, Suco rayado
