# Supplementary material for: Popular interest in vertebrates does not reflect extinction risk and is associated with bias in conservation investment
Source: PLoS One. 2018 Sep 26;13(9):e0203694. doi: 10.1371/journal.pone.0203694 (PMC6157853; doi:10.1371/journal.pone.0203694)
Supplement: S2 Table — Data corresponding to Fig 2b. (PDF) [file pone.0203694.s003.pdf]

S2 Table. The top 100 most Googled mammals in the world. Data corresponding to Figure 2b.

Rank	Species	Common names	Average monthly web search interest	All common names
1	*Panthera tigris*	Tiger	29765.26	Tiger, Tigre
2	*Panthera leo*	Lion	22977.01	Lion, African Lion, Lion d'Afrique, León
3	*Canis lupus*	Gray Wolf	16248.67	Gray Wolf, Tundra Wolf, Arctic Wolf, Grey Wolf, Mexican Wolf, Plains Wolf, Timber Wolf, Common Wolf, Wolf, Loup, Loup Gris, Loup Vulgaire, Lobo, Lobo
4	*Ailuropoda melanoleuca*	Giant Panda	8681.54	Giant Panda, Panda, Panda géant, Panda Gigante
5	*Giraffa camelopardalis*	Giraffe	6278.98	Giraffe
6	*Alces alces*	Moose	5489.53	Moose, Eurasian Elk, Elk, European Elk, Eurasian Moose, Siberian Elk, Élan, Alce
7	*Puma concolor*	Puma	5236.48	Puma, Mountain Lion, Cougar, Red Tiger, Deer Tiger, León Americano, León Bayo, León Colorado, León De Montaña, Mitzli, Onza Bermeja
8	*Acinonyx jubatus*	Cheetah	4727.21	Cheetah, Hunting Leopard, Guépard, Chita, Guepardo
9	*Ursus maritimus*	Polar Bear	4259.88	Polar Bear, Ours polaire, Ours blanc, Oso Polar
10	*Orcinus orca*	Killer Whale	3951.28	Killer Whale, Orca, Orque, Epaulard, Espadarte, Espadarte, Orca
11	*Panthera pardus*	Leopard	3818.59	Leopard, Panthère, Léopard, Leopardo, Pantera
12	*Phascolarctos cinereus*	Koala	2377.63	Koala
13	*Meles meles*	Eurasian Badger	2249.35	Eurasian Badger, Badger, BLAIREAU EUROPÉEN, Blaireau Européen, Tejón, TEJÓN
14	*Ursus arctos*	Brown Bear	2145.13	Brown Bear, Mexican Grizzly Bear, Grizzly Bear, Ours brun, Oso Pardo
15	*Pan paniscus*	Bonobo	2105.62	Bonobo, Pygmy Chimpanzee, Gracile Chimpanzee, Dwarf Chimpanzee, Chimpanzé Nain, Chimpanzé pygmée, Chimpancé Pigmeo
16	*Pan troglodytes*	Chimpanzee	2027.21	Chimpanzee, Robust Chimpanzee, Common Chimpanzee, Chimpanzé, Chimpancé
17	*Panthera onca*	Jaguar	1817.09	Jaguar, Tigre Real, Tigre Americano, Otorongo, Yaguar, Yaguarete
18	*Balaenoptera musculus*	Blue Whale	1526.06	Blue Whale, Sibbold's Rorqual, Sulphur-bottom Whale, Pygmy Blue Whale, Baleinoptère bleue, Baleine bleue, Baleine d'ostende, Rorqual à ventre cannelé, Rorqual Bleu, Rorqual de Sibbold, Ballena Azul, Rorcual Azul
19	*Mellivora capensis*	Honey Badger	1443.16	Honey Badger
20	*Orycteropus afer*	Aardvark	1394.34	Aardvark, Antbear, Oryctérope
21	*Felis silvestris*	Wild Cat	1376.77	Wild Cat, Wildcat, Chat Sauvage, Chat Orné, Gato Montés, Gato Silvestre
22	*Suricata suricatta*	Meerkat	1307.15	Meerkat, Slender-tailed Meerkat, Suricate
23	*Vulpes vulpes*	Red Fox	1174.18	Red Fox, Silver Fox, Cross Fox, RENARD, Renard Roux, ZORRO, Zorro Rojo
24	*Gorilla gorilla*	Western Gorilla	1165.4	Western Gorilla, Lowland Gorilla, Gorille, Gorila
25	*Ailurus fulgens*	Red Panda	1139.68	Red Panda, Lesser Panda, Red Cat-bear, Panda Éclatant, Petit Panda, Panda Chico, Panda Rojo

S2 Table continued

Rank	*Species*	Common names	Average monthly web search interest	All common names
26	*Hippopotamus amphibius*	Hippopotamus	1069.43	Hippopotamus, Large Hippo, Common Hippopotamus, Hippopotame, Hipopótamo Anfibio
27	*Homo sapiens*	Human	922.66	Human
28	*Mustela erminea*	Ermine	892.55	Ermine, Stoat, Short-tailed Weasel, Hermine, Armiño
29	*Ornithorhynchus anatinus*	Platypus	859.31	Platypus, Duck-billed Platypus, Ornithorynque
30	*Sarcophilus harrisii*	Tasmanian Devil	837.98	Tasmanian Devil, Diable De Tasmanie
31	*Megaptera novaeangliae*	Humpback Whale	768.36	Humpback Whale, Hump Whale, Hunchbacked Whale, Bunch, Mégaptère, Baleine à bosse, Baleine à taquet, Jubarte, Rorqual à bosse, Rorqual du Cap, Ballena Jorobada, Gubarte, Jorobada, Rorcual Jorobado
32	*Cervus elaphus*	Red Deer	760.21	Red Deer, Bactrian Wapiti, Bokharan Deer, Bukhara Red Deer, Elk, Bactrian Deer, Wapiti, Bactrian Red Deer, Cerf De Bactriane, Cerf Du Turkestan, Cerf Élaphe, Cerf Élaphe Du Turkestan, Cerf Rouge Du Turkestan, Ciervo, Ciervo Bactriano, Ciervo Rojo
33	*Martes pennanti*	Fisher	720.69	Fisher
34	*Vulpes zerda*	Fennec Fox	694.97	Fennec Fox, Fennec
35	*Loxodonta africana*	African Elephant	655.46	African Elephant, Éléphant d'Afrique, Éléphant Africain, Elefante Africano
36	*Canis latrans*	Coyote	653.58	Coyote, American Jackal, Brush Wolf, Prairie Wolf
37	*Physeter macrocephalus*	Sperm Whale	651.07	Sperm Whale, Spermacet Whale, Cachelot, Pot Whale, Cachalot, Ballena Esperma, Ballena Esperma, Cachalote, Cachalote
38	*Dugong dugon*	Dugong	649.19	Dugong, Sea Cow, Dugon
39	*Panthera uncia*	Snow Leopard	541.3	Snow Leopard, Ounce, Once, Irbis, Léopard des neiges, Panthère des neiges, Leopardo Nival, Pantera de la Nieves
40	*Urocyon cinereoargenteus*	Grey Fox	523.11	Grey Fox, Tree Fox, Gray Fox, Gato De Monte, Gato Cervan, Zorro, Zorro Gris, Zorro Plateado
41	*Eubalaena glacialis*	North Atlantic Right Whale	516.21	North Atlantic Right Whale, Northern Right Whale, Right Whale, Black Right Whale, Baleine De Biscaye, Baleine Des Basques, Ballena, Ballena Franca Del Norte, Ballenga
42	*Mustela nivalis*	Least Weasel	451.61	Least Weasel, Weasel, Belette d'Europe, Comadreja
43	*Gulo gulo*	Wolverine	436.55	Wolverine, GLOUTON, Glouton, Glotón, GLOTÓN
44	*Canis rufus*	Red Wolf	423.38	Red Wolf
45	*Elephas maximus*	Asian Elephant	417.48	Asian Elephant, Indian Elephant, Eléphant D'Asie, Eléphant D'Inde, Elefante Asiático
46	*Nycticebus coucang*	Greater Slow Loris	416.88	Greater Slow Loris, Sunda Slow Loris, Slow Loris, Loris Lent, Loris Lento
47	*Tursiops truncatus*	Common Bottlenose Dolphin	411.49	Common Bottlenose Dolphin, Bottlenose Dolphin, Bottle-nosed Dolphin, Bottlenosed Dolphin, Grand Dauphin, dauphin souffleur, grand dauphin, Souffleur, Tursiops, Delfín Mular, Pez Mular, Tursión, Tursión
48	*Bubalus arnee*	Asian Buffalo	397.11	Asian Buffalo, Asiatic Buffalo, Indian Buffalo, Indian Water Buffalo, Water Buffalo, Wild Asian Buffalo, Wild Water Buffalo, Buffle D'Eau, Buffle De L'Inde, Bufalo Arni
49	*Enhydra lutris*	Sea Otter	395.91	Sea Otter, Loutre De Mer, Nutria Del Kamtchatka, Nutria Marina

S2 Table continued

Rank	*Species*	Common names	Average monthly web search interest	All common names
50	*Potos flavus*	Kinkajou	334.02	Kinkajou, Mico De Noche, Cusu, Martilla, Chosna, Mico León, Mono Michi, Perro De Monte
51	*Phacochoerus africanus*	Common Warthog	332.95	Common Warthog, Warthog, Eritrean Warthog, Phacochère Commun
52	*Martes martes*	European Pine Marten	314.3	European Pine Marten, Pine Marten, Pine Martin, European Pine Martin, Martre Des Pins, MARTRE DES PINS, Marta, MARTA
53	*Sciurus carolinensis*	Eastern Gray Squirrel	305.58	Eastern Gray Squirrel, Gray Squirrel, Grey Squirrel
54	*Speothos venaticus*	Bush Dog	280.81	Bush Dog, Vinegar Dog, Savannah Dog, Chien Des Buissons, Zorro, Perrito Venadero, Cachorro Vinagre, Guanfando, Pero Selvático, Perrito de Monte , Perro de Agua, Perro de la Selva, Perro De Monte, Perro Grullero , Perro Vinagre , Umba, Zorrito Vinagre, Zorro Pitoco , Zorro Vinagre
55	*Heterocephalus glaber*	Naked Mole Rat	250.54	Naked Mole Rat
56	*Equus africanus*	African Wild Ass	248.02	African Wild Ass, Ass, African Ass, Ane sauvage d'Afrique, Âne Sauvage D'Afrique, Asno Salvaje de Africa
57	*Alopex lagopus*	Arctic Fox	242.86	Arctic Fox, Polar Fox, Renard Polaire, Isatis, Reynard Polaire, Zorro Ártico
58	*Odocoileus hemionus*	Mule Deer	239.31	Mule Deer, Black-tailed Deer, Cedros Island Mule Deer, Cedros Island Black-tailed Deer, Bura, Venado Mulo(a)
59	*Rangifer tarandus*	Reindeer	236.05	Reindeer, Caribou, Peary Caribou, Renne, RENNE, Reno, RENO
60	*Antilocapra americana*	Pronghorn	227.7	Pronghorn, Mexican Pronghorn, Antilocapre, Antilope Américaine, Berrendo
61	*Capreolus capreolus*	European Roe Deer	218.06	European Roe Deer, Western Roe Deer, Roe Deer, Chevreuil Européen, Chevreuil, CHEVREUIL EUROPÉEN, Corzo, CORZO
62	*Alouatta seniculus*	Colombian Red Howler Monkey	214.6	Colombian Red Howler Monkey, Colombian Red Howling Monkey, Mono Araguato, Coto Mono, Mono, Mono Colorado, Mono Cotudo, Roncador
63	*Eschrichtius robustus*	Gray Whale	202.5	Gray Whale, Grey Whale
64	*Neofelis nebulosa*	Clouded Leopard	195.11	Clouded Leopard, Panthère Longibande, Panthère Nébuleuse, Pantera Del Himalaya, Pantera Longibanda, Pantera Nebulosa
65	*Mandrillus sphinx*	Mandrill	194.65	Mandrill, Mandril
66	*Desmodus rotundus*	Common Vampire Bat	193.06	Common Vampire Bat, Vampire Bat
67	*Gorilla beringei*	Eastern Gorilla	181.76	Eastern Gorilla, Mountain Gorilla
68	*Myrmecobius fasciatus*	Numbat	170	Numbat, Banded Anteater, Fourmilier Marsupial Rayé, Hormiguero Marsupial
69	*Dama dama*	Fallow Deer	161.13	Fallow Deer, Mesopotamian Fallow Deer, Persian Fallow Deer, FALLOW DEER, Daim Européen, DAIM EUROPÉEN, Gamo, GAMO
70	*Hydrurga leptonyx*	Leopard Seal	156.7	Leopard Seal
71	*Vulpes macrotis*	Kit Fox	153.45	Kit Fox, Desert Fox, Zorra Del Desierto, Zorra Norteña
72	*Okapia johnstoni*	Okapi	152.27	Okapi
73	*Sus scrofa*	Wild Boar	149.61	Wild Boar, Eurasian Wild Pig, Ryukyu Islands Wild Pig, Sanglier, Sanglier D'Eurasie, Jabalí
74	*Cebuella pygmaea*	Pygmy Marmoset	149.41	Pygmy Marmoset, Chambira, Chichico, Leoncillo, Leonzito, Micoleãozinho, Mono De Bolsillo, Titi, Titi-pielroja

S2 Table continued

Rank	*Species*	Common names	Average monthly web search interest	All common names
75	*Macropus robustus*	Common Wallaroo	148.35	Common Wallaroo, Euro, Barrow Island Euro, Hill Wallaroo
76	*Pudu puda*	Southern Pudu	147.16	Southern Pudu, Chilean Pudu, Poudou Du Sud, Pudu Du Sud, Ciervo Enano, Pudu, Pudu Meridional, Venadito, Venado
77	*Macrotis lagotis*	Bilby	146.85	Bilby, Greater Rabbit-eared Bandicoot, Greater Bilby, Dalgyte, Grand Bandicoot-lapin, Grand Péramèle-lapin, Cangurito Narigudo Grande
78	*Myrmecophaga tridactyla*	Giant Anteater	145.72	Giant Anteater, Tamanoir, Grand Fourmilier, Hormiguero Gigante, Oso Caballo, Oso Hormiguero, Oso Palmero
79	*Balaenoptera physalus*	Fin Whale	145.1	Fin Whale, Fin-backed Whale, Finner, Common Rorqual, Herring Whale, Razorback, Finback, Baleine à nageoires, Baleine fin, Baleinoptère commune, Rorqual commun, Rorqual Commun, Ballena Aleta, Ballena Boba, Rorcual Común
80	*Martes foina*	Stone Marten	143.96	Stone Marten, Beech Marten, Fouine, Garduña
81	*Lycaon pictus*	African Wild Dog	142.26	African Wild Dog, Painted Hunting Dog, Cape Hunting Dog, Cynhyene, Loup-peint, Lycaon, Licaon
82	*Odocoileus virginianus*	White-tailed Deer	141.44	White-tailed Deer, Key Deer, Key Deer Toy Deer, Cariacú, Venado Cola Blanca
83	*Aepyceros melampus*	Impala	135.77	Impala, Black-faced Impala
84	*Odobenus rosmarus*	Walrus	135.61	Walrus, Morse, MORSA
85	*Sciurus niger*	Bryant's Fox Squirrel	128.44	Bryant's Fox Squirrel, Delmarva Fox Squirrel, Eastern Fox Squirrel, Fox Squirrel
86	*Melursus ursinus*	Sloth Bear	128.13	Sloth Bear, Ours prochile lippu, Ours lippu de l'Inde, Oso Perezoso
87	*Inia geoffrensis*	Boto	128.08	Boto, Pink River Dolphin, Boutu, Amazon River Dolphin, Dauphin De L'Amazone, nia, Bufeo
88	*Macropus rufus*	Red Kangaroo	127.93	Red Kangaroo
89	*Arctictis binturong*	Binturong	120.81	Binturong, Bearcat, Palawan Binturong
90	*Chrysocyon brachyurus*	Maned Wolf	114.37	Maned Wolf, Loup À Crinière, Aguara Guazu, Borochi, Lobo De Crin
91	*Leopardus pardalis*	Ocelot	113.54	Ocelot, Manigordo, Gato Onza, Ocelote, Tigrillo
92	*Rattus rattus*	House Rat	111.17	House Rat, Ship Rat, Roof Rat, Black Rat, Rat Noir, Rata Negra
93	*Tremarctos ornatus*	Spectacled Bear	109.98	Spectacled Bear, Andean Bear, Ours Andin, Ours à lunettes, Oso de Anteojos, Oso Frontino, Oso Real
94	*Ondatra zibethicus*	Muskrat	108.54	Muskrat
95	*Balaenoptera acutorostrata*	Common Minke Whale	106.22	Common Minke Whale, Minke Whale, Lesser Rorqual, Little Piked Whale, Dwarf Minke Whale, Baleinoptère à museau pointu, Petit rorqual, Ballena Minke, Rorcual Menor
96	*Helarctos malayanus*	Sun Bear	104.62	Sun Bear, Malayan Sun Bear, Ours Malais, Ours des cocotiers, Oso de Sol, Oso Malayo
97	*Tamandua mexicana*	Northern Tamandua	102.92	Northern Tamandua, Oso Melero, Oso Hormiguero, Oso Mielero, Tamandua
98	*Pagophilus groenlandicus*	Harp Seal	102.66	Harp Seal, Greenland Seal, FOCA PÍA
99	*Diceros bicornis*	Black Rhinoceros	99.38	Black Rhinoceros, Hook-lipped Rhinoceros, Rhinocéros noir, Rinoceronte Negro
100	*Cervus nippon*	Sika Deer	97.87	Sika Deer, Sika, Shansi Sika
