# Supplementary material for: Popular interest in vertebrates does not reflect extinction risk and is associated with bias in conservation investment
Source: PLoS One. 2018 Sep 26;13(9):e0203694. doi: 10.1371/journal.pone.0203694 (PMC6157853; doi:10.1371/journal.pone.0203694)
Supplement: S3 Table — Data corresponding to Fig 2c. (PDF) [file pone.0203694.s004.pdf]

S3 Table. The top 100 most Googled reptiles in the world. Data corresponding to Figure 2c.

Rank	Species	Common names	Average monthly web search interest	All common names
1	*Dermochelys coriacea*	Leatherback	522.48	Leatherback, Leatherback Sea Turtle, Leathery Turtle, Luth, Trunkback Turtle, Trunk Turtle, Coffin-back, Tortue luth, Cardon, Baula, Canal, Dorso de Cuero, Galapagos, Siete Lomos, Siete Quillas, Tinglada, Tinglar, Tora, Tortuga Caná, Tortuga Laud
2	*Ophiophagus hannah*	King Cobra	481.09	King Cobra, Hamadryad
3	*Plestiodon tetragrammus*	Four-lined Skink	275.45	Four-lined Skink, Escorpion De Cola Azul, Lince
4	*Heloderma suspectum*	Gila Monster	253.26	Gila Monster, Lézard perlé, Monstre de gila, Monstruo de Gila
5	*Storeria dekayi*	Brownsnake	167.68	Brownsnake, Brown Snake
6	*Caretta caretta*	Loggerhead	155.36	Loggerhead, Tortue Caouanne, Caouanne, Cayuma, Tortuga Boba
7	*Chelonia mydas*	Green Turtle	147.73	Green Turtle, Tortue verte, Tortue franche, Tortue comestible, Tortuga Blanca, Tortuga Verde
8	*Dendroaspis polylepis*	Black Mamba	143.09	Black Mamba
9	*Vipera berus*	Adder	143.09	Adder
10	*Varanus komodoensis*	Komodo Dragon	135.04	Komodo Dragon, Komodo Monitor, Ora, Dragon Des Komodos, Varan De Komodo, Varano De Komodo
11	*Coluber constrictor*	Eastern Racer	125.92	Eastern Racer, Racer
12	*Crocodylus acutus*	American Crocodile	121.64	American Crocodile, Crocodile d'Amérique, Crocodile Americain, Cocodrilo Americano, Caiman, Caiman de costa, Caiman aguja, Cocodrilo de Río, Lagarto Amarillo, Lagarto Real
13	*Agkistrodon piscivorus*	Cottonmouth	114.62	Cottonmouth
14	*Natrix natrix*	Grass Snake	87.24	Grass Snake, Couleuvre à collier, Culebra de Collar
15	*Alligator mississippiensis*	American Alligator	83.43	American Alligator, Mississippi Alligator
16	*Hypsirhynchus ater*	Black Racer	82.62	Black Racer
17	*Diadophis punctatus*	Ring-necked Snake	79.16	Ring-necked Snake, Ringneck Snake
18	*Crocodylus niloticus*	Nile Crocodile	77.14	Nile Crocodile
19	*Chlamydosaurus kingii*	Frilled Lizard	72.32	Frilled Lizard
20	*Epicrates subflavus*	Jamaican Boa	60.55	Jamaican Boa, Yellow Snake, Boa De La Jamaïque, Boa De Jamaica
21	*Chelydra serpentina*	Snapping Turtle	55.17	Snapping Turtle, North American Snapping Turtle, Common Snapping Turtle
22	*Podocnemis expansa*	South American River Turtle	55.12	South American River Turtle, Arrau, Giant South American Turtle, Tartaruga, Podocnémide Élargie
23	*Gavialis gangeticus*	Gharial	48.01	Gharial, Indian Gharial, Fish-eating Crocodile, Indian Gavial, Gavial, Long-nosed Crocodile, Gavial du Gange, Gavial del Ganges
24	*Sphenodon punctatus*	Beak-head	40.49	Beak-head, Cook Strait Tuatara, Sphenodon, Tuatara, Hatteria Ponctué, Sphénodon Ponctué, Tuátara

S3 Table continued

Rank	Species	Common names	Average monthly web search interest	All common names
25	*Sistrurus catenatus*	Massasauga	40.43	Massasauga
26	*Terrapene carolina*	Eastern Box Turtle	36.43	Eastern Box Turtle, Mexican Box Turtle, American Box Turtle, Common Box Turtle, Yucatan Box Turtle, Tortue-boîte de Caroline
27	*Pantherophis guttatus*	Corn Snake	35.95	Corn Snake, Red Cornsnake
28	*Thelotornis capensis*	Bird Snake	34.1	Bird Snake, Twig Snake, Vine Snake
29	*Eretmochelys imbricata*	Hawksbill Turtle	32.78	Hawksbill Turtle, Tortue à écailles, Tortue à bec faucon, Caret, Tortue Caret, Tortue imbriquée, Tortuga de Carey
30	*Pituophis catenifer*	Bullsnake	32.11	Bullsnake, Gophersnake, Gopher Snake
31	*Morelia spilota*	Carpet Python	32.05	Carpet Python, Diamond Python, Western Australian Carpet Python
32	*Lepidochelys olivacea*	Olive Ridley	30.32	Olive Ridley, Pacific Ridley, Ridley du Pacifique, Tortue Bâtarde, Tortue de Ridley, Tortue Olivâtre, Tortuga Golfina, Tortuga Carpintera, Tortuga Guaraguá, Tortuga Lora, Tortuga Manila, Tortuga Mulato, Tortuga Olivacea, Tortuga Parlama
33	*Trachemys scripta*	Yellow-bellied Slider Turtle	30.15	Yellow-bellied Slider Turtle, Red-eared Slider Turtle, Cumberland Slider Turtle, Slider, Common Slider
34	*Heloderma horridum*	Beaded Lizard	29.52	Beaded Lizard, Héloderme granuleux, Héloderme horrible, Escorpión, Lagarto de Cuentas
35	*Crocodylus porosus*	Salt-water Crocodile	28.97	Salt-water Crocodile, Estuarine Crocodile, Crocodile d'estuaire, Crocodile marin, Cocodrilo Poroso
36	*Pituophis melanoleucus*	Pinesnake	28.66	Pinesnake, Pine Snake
37	*Morelia viridis*	Green Python	27.79	Green Python, Green Tree Python
38	*Drymarchon couperi*	Eastern Indigo Snake	27.4	Eastern Indigo Snake
39	*Malaclemys terrapin*	Diamondback Terrapin	27.09	Diamondback Terrapin, Terrapin
40	*Crotalus adamanteus*	Eastern Diamond-backed Rattlesnake	25.43	Eastern Diamond-backed Rattlesnake, Eastern Diamondback Rattlesnake
41	*Ctenosaura defensor*	Yucatán Spiny-tailed Iguana	25.42	Yucatán Spiny-tailed Iguana, Chop
42	*Opheodrys aestivus*	Greensnake	25.24	Greensnake, Rough Greensnake, Rough Green Snake
43	*Melanosuchus niger*	Black Caiman	24.4	Black Caiman, Caïman noir, Caimán Negro, Lagarto Negro
44	*Regina septemvittata*	Queen Snake	24.24	Queen Snake
45	*Glyptemys insculpta*	Wood Turtle	24.11	Wood Turtle, Clemmyde Sculptée
46	*Thamnophis sirtalis*	San Francisco Garter Snake	24.05	San Francisco Garter Snake
47	*Philothamnus irregularis*	Common Bush Snake	23.92	Common Bush Snake, Green Tree Snake, Irregular Green Snake, Northern Green Bush Snake
48	*Dendrelaphis calligastra*	Coconut Tree Snake	23.86	Coconut Tree Snake, Green Tree Snake, Nothern Tree Snake
50	*Crotalus atrox*	Western Diamond-backed Rattlesnake	23.54	Western Diamond-backed Rattlesnake, Western Diamondback Rattlesnake
49	*Sistrurus miliarius*	Pygmy Rattlesnake	23.76	Pygmy Rattlesnake

S3 Table continued

Rank	Species	Common names	Average monthly web search interest	All common names
51	*Agkistrodon bilineatus*	Cantil	22.5	Cantil, Castellana
52	*Pseudemydura umbrina*	Western Swamp Tortoise	22.49	Western Swamp Tortoise, Western Swamp Turtle, Short-necked Tortoise, Western Short-necked Turtle, Pseudémydure de Perth, Tortue à col court, Tortuga Serpentina Occidental
53	*Lepidochelys kempii*	Kemp's Ridley	21.93	Kemp's Ridley, Atlantic Ridley, Mexican Ridley, Gulf Ridley, Ridley de Kemp, Lépidochelyde de Kemp, Tortue de Kemp, Cotorra, Tortuga Iora, Tortuga Marina Bastarda
54	*Alligator sinensis*	Chinese Alligator	21.85	Chinese Alligator, China Alligator, Alligator de Chine, Aligator de China
55	*Thamnophis sauritus*	Eastern Ribbonsnake	21.24	Eastern Ribbonsnake, Eastern Ribbon Snake
56	*Crotalus horridus*	Timber Rattlesnake	21.24	Timber Rattlesnake
57	*Heterodon platirhinos*	Eastern Hog-nosed Snake	20.34	Eastern Hog-nosed Snake, Eastern Hognose Snake
58	*Farancia abacura*	Mud Snake	19.47	Mud Snake, Red-bellied Mudsnake
59	*Crotalus scutulatus*	Mohave Rattlesnake	19.36	Mohave Rattlesnake, Mojave Rattlesnake
60	*Crotalus cerastes*	Sidewinder	19.12	Sidewinder
61	*Caiman crocodilus*	Common Caiman	19.05	Common Caiman, Spectacled Caiman
62	*Macrochelys temminckii*	Alligator Snapping Turtle	18.88	Alligator Snapping Turtle
63	*Liochlorophis vernalis*	Smooth Greensnake	18.87	Smooth Greensnake, Smooth Green Snake
64	*Hypnale nepa*	Sri Lanka Humpnose Viper	18.62	Sri Lanka Humpnose Viper , Geckos, Gloyd's Hump-nosed Viper, Wall's Humpnose Viper
65	*Phrynosoma cornutum*	Texas Horned Lizard	18.44	Texas Horned Lizard
66	*Cantoria annulata*	Banded Watersnake	18.23	Banded Watersnake, Banded Water Snake
67	*Zootoca vivipara*	Common Lizard	17.03	Common Lizard, Viviparous Lizard, Lagartija de Turbera
68	*Naja kaouthia*	Monocled Cobra	16.73	Monocled Cobra, Monocellate Cibra
69	*Bothropoides insularis*	Golden Lancehead	16.53	Golden Lancehead, Queimada Island Bothrops
70	*Lacerta agilis*	Sand Lizard	16.13	Sand Lizard, Lezard des Souches, Lagarto Ágil
71	*Crocodylus palustris*	Mugger	16	Mugger, Muggar, Broad-snouted Crocodile, Marsh Crocodile, Crocodile des Marais, Crocodile paludéen, Crocodile palustre, Cocodrilo del Marjal, Cocodrilo Marismeño
72	*Nerodia sipedon*	Lake Erie Water Snake	15.92	Lake Erie Water Snake
73	*Gopherus agassizii*	Desert Tortoise	15.44	Desert Tortoise, Tortue D'Agassiz, Gophère D'Agassiz, Tortuga Del Desierto
74	*Crocodylus rhombifer*	Cuban Crocodile	15.41	Cuban Crocodile, Crocodile de Cuba, Cocodrilo de Cuba
75	*Enhydrina schistosa*	Beaked Sea Snake	15.26	Beaked Sea Snake, Enhydrine Ardoisee

S3 Table continued

Rank	Species	Common names	Average monthly web search interest	All common names
76	*Crotalus viridis*	Prairie Rattlesnake	15.2	Prairie Rattlesnake
77	*Natator depressus*	Flatback	14.65	Flatback, Tortue marine à dos plat, Chelonée à dos plat, Tortuga Franca Oriental
78	*Python regius*	Ball Python	14.14	Ball Python, Royal Python, Python Royal
79	*Cemophora coccinea*	Scarletsnake	14	Scarletsnake, Scarlet Snake
80	*Thamnophis marcianus*	Checkered Gartersnake	13.66	Checkered Gartersnake, Checkered Garter Snake
81	*Hemachatus haemachatus*	Ringhals	13.6	Ringhals, Ring-necked Spitting Cobra, Rinkhals, Sépédon Hémachate
82	*Agkistrodon contortrix*	Northern Copperhead	12.91	Northern Copperhead
83	*Chrysemys picta*	Painted Turtle	12.47	Painted Turtle
84	*Bitis rhinoceros*	Rhinoceros Viper	12.01	Rhinoceros Viper, Western Gaboon Adder, West African Gaboon Viper, West African Gaboon Adder, Western Gaboon Viper, Vipère du Gabon de l'Ouest
85	*Nerodia rhombifer*	Diamond-backed Watersnake	11.94	Diamond-backed Watersnake, Diamondback Water Snake, Culebra De Agua De Diamantes
86	*Cyrtodactylus louisiadensis*	Ring-tailed Gecko	11.89	Ring-tailed Gecko, Banded Gecko
87	*Amblyrhynchus cristatus*	Fernandina Marine Iguana	11.77	Fernandina Marine Iguana, Sea Iguana, Galápagos Marine Iguana, Marine Iguana, Galapagos Marine Iguana, Amblyrhynche à crête, Iguane marin, Iguana Marina
88	*Farancia erytrogramma*	Rainbow Snake	11.7	Rainbow Snake
89	*Aspidites ramsayi*	Ramsay's Python	11.65	Ramsay's Python, Woma, Python De Ramsay, Pitón De Ramsay
90	*Lampropeltis getula*	Common Kingsnake	11.59	Common Kingsnake
91	*Ctenosaura flavidorsalis*	Yellow-backed Spiny-tailed Iguana	11.51	Yellow-backed Spiny-tailed Iguana, Rumia
92	*Acrochordus javanicus*	Elephant Trunk Snake	11.12	Elephant Trunk Snake, Javan File Snake, Elephant's Trunk Snake, Javan Wart Snake
93	*Charina bottae*	Rubber Boa	11.06	Rubber Boa, Southern Rubber Boa
94	*Lichanura trivirgata*	Rosy Boa	10.59	Rosy Boa
95	*Crocodylus intermedius*	Orinoco Crocodile	10.1	Orinoco Crocodile, Crocodile de l'Orénoque, Cocodrilo del Orinoco
96	*Storeria occipitomaculata*	Red-bellied Snake	9.97	Red-bellied Snake, Redbelly Snake
97	*Crocodylus johnsoni*	Australian Freshwater Crocodile	8.53	Australian Freshwater Crocodile, Johnson's Crocodile, Freshie, Johnstone's Crocodile, Johnston's Crocodile
98	*Fordonia leucobalia*	Crab-eating Snake	8.11	Crab-eating Snake, Crab-eating Water Snake, Fordon's Water Snake, Mangrove Snake, The Fordonia, The Plain Fordonia, White-bellied Freshwater Snake, White-bellied Mangrove Snake, White-bellied Water Snake
99	*Coelognathus flavolineatus*	Yellow-striped Trinket Snake	7.36	Yellow-striped Trinket Snake, Yellow striped Snake, Black Copper Rat Snake
100	*Bothropoides lutzi*	Cerrado Lancehead	6.64	Cerrado Lancehead, Boca de Sapo
