# Supplementary material for: Popular interest in vertebrates does not reflect extinction risk and is associated with bias in conservation investment
Source: PLoS One. 2018 Sep 26;13(9):e0203694. doi: 10.1371/journal.pone.0203694 (PMC6157853; doi:10.1371/journal.pone.0203694)
Supplement: S4 Table — Data corresponding to Fig 2d. (PDF) [file pone.0203694.s005.pdf]

**S4 Table. The top 100 most Google fish in the world.** Data corresponding to Figure 2d.

| Rank | Species | Common names | Average monthly web search interest | All common names |
|---|---|---|---|---|
| 1 | *Sphyrna mokarran* | Great Hammerhead | 2981.86 | Great Hammerhead, Hammerhead Shark, Squat-headed Hammerhead Shark, Grand Requin-marteau, Marieau Millet, Poisson Pantouflier, Sorosena, Cornuda, El Tiburon, Guardia Civil, Pez Martillo, Tiburon |
| 2 | *Carcharodon carcharias* | Great White Shark | 1736.18 | Great White Shark |
| 3 | *Chaetodon capistratus* | Butterbun | 1166.02 | Butterbun, Butterfly, Four-eye Butterflyfish, Foureye Butterflyfish, Foureye Butterflyfish, Four-eyed butteflyfish, Kete, School Mistress, Marguerite, Parché, Isabelita, Isabelita blanca, Kete, Mariposa, Mariposa ocelada, Parche, Parche ocelado |
| 4 | *Dicentrarchus labrax* | European Seabass | 1055 | European Seabass, Common Bass, European Bass, Bass, King Of The Mullets, Sea Dace, Sea Perch, White Mullet, White Salmon, Capemouth, Loubas Negre, Bar, Bar Commun, Bar Européen, Bar Franc, Bog, Brigue, Drelique, Gutgareo, Loubine, Loup, Loupassou, Loup de Mer, Lubin, Luvassu, Pigne, Baieta, Baila, Cherne, Llop, Llubina, Lubaro, Lubina, Mero, Pintat, Robaliza, Robalo |
| 5 | *Pomacanthus imperator* | Emperor Angelfish | 977.23 | Emperor Angelfish, Imperial Angelfish, Angelfish, Emperor, Ange de mer impérial, Holacanthe empereur, Poisson ange impérial |
| 6 | *Chanodichthys dabryi* | Humpback | 963.43 | Humpback |
| 7 | *Selene brevoortii* | Airfin lookdown | 882.52 | Airfin lookdown, Hairfin lookdown, Mexican lookdown, Mexican lookdown (FB), Musso corcovade, Antena, Cara caballa, Carita, Carita jorobada, Espejuelo, Jorobado, Jorobado antena, Jorobado mexicano, Radio |
| 8 | *Coryphaena hippurus* | Common Dolphinfish | 707.52 | Common Dolphinfish, Dolphinfish, Dolphin Fish, Dorado, Green Dolphin, Mahimahi, Mahi-mahi, Mahi Mahi, Common Dolphin Fish, Clic, Coryphène Commune, Dauphin, Dorade, Dorade Coryphène, Dorado Común, Dorado Delfin, Lampuga, Llampuga |
| 9 | *Rhincodon typus* | Whale Shark | 620.33 | Whale Shark, Requin Baleine, Tiburón Ballena |
| 10 | *Mormyrus kannume* | Bottlenose | 603.4 | Bottlenose |
| 11 | *Mitsukurina owstoni* | Elfin Shark | 569.52 | Elfin Shark, Goblin Shark, Requin Lutin, Tiburones Duende |
| 12 | *Oplegnathus insignis* | Pacific Beakfish | 531.89 | Pacific Beakfish, Loreta, Loro, San Pedro, Tigris |
| 13 | *Haemulopsis leuciscus* | Raucous grunt | 463.52 | Raucous grunt, White grunt, Boquimorado chato, Negro, Ronco roncacho, Ronco ruco |
| 14 | *Alphestes multiguttatus* | Rivulated Mutton Hamlet | 459.76 | Rivulated Mutton Hamlet, Rock Bass, Sea Bass, Pacific Guaseta, Vareche Veine, Varech Veine, Cherne, Cabrilla, Colorado, Companero De Mero, Guaseta Rayada, Guaseta Rayado, Guato, Mero |
| 15 | *Squatina squatina* | Angel Shark | 400.17 | Angel Shark, L'ange, Ange De Mer, Angel, Antjou, Bourgeois, Bourget, L'anelot, Ange, Martrame, Mordacle, Squatine Occelee, Angelote, Mermejuela, Pardon, Pez Angel |
| 16 | *Cetorhinus maximus* | Basking Shark | 387.86 | Basking Shark, Pelerin, Peregrino |
| 17 | *Alopias vulpinus* | Common Thresher Shark | 330.83 | Common Thresher Shark, Renard, Zorro |
| 18 | *Scomberomorus cavalla* | King Mackerel | 321.51 | King Mackerel, Kingfish, Maquereau, Thazard Barré, Carite Sierra, Carite, Carite Lucio, Carito, Carito Lucio, Peto, Rey, Serrucho, Sierra |
| 19 | *Hippocampus hippocampus* | Short-snouted Seahorse | 281.28 | Short-snouted Seahorse, Short Snouted Seahorse, Sea Horse, Hippocampe, Cheval de Mer, Hippocampe à Museau Court, Caballito de Mar, Caballo Marino, Cabalo de Mar |

## S4 Table continued

| Rank | Species | Common names | Average monthly web search interest | All common names |
|---|---|---|---|---|
| 20 | *Scatophagus argus* | Spotted Scat | 277.15 | Spotted Scat, Spotted Butt, Butter Fish, Common Scat, Leopard Scat, Scat, Butterfish, Spotted Butterfish, Spotted Butter Fish, Spotted Scad, Argus Fish, Pavillon Tacheté, Pingo Manchado |
| 21 | *Scarus guacamaia* | Rainbow Parrotfish | 249.15 | Rainbow Parrotfish, Blue Rainbow, Rainbow, Perroquet arc-en-ciel, Guacamaia, Guacamaya, Loro Guacamayo |
| 22 | *Paralonchurus dumerilii* | Suco croaker | 239.51 | Suco croaker, Bourrugue suco, Barcelona, Lambe suco, Suco rayado |
| 23 | *Balistes vetula* | Old Wife | 236.73 | Old Wife, Ol'wife, Queen Triggerfish, Triggerfish, Turbot, Baliste Royal, Bourse, Cach??a, Cach, ö, ´a, Cachúa, Cachuda, Cochino, Oldwife, Pejepuerco, Peje Puerco, Pejepuerco Cachuo, Pez Puerco, Puerco, Sol |
| 24 | *Chaetodon sedentarius* | Reef Butterflyfish | 226.57 | Reef Butterflyfish, School Mistress, Least Butterflyfish, Butterbun, Katy, Isabelita Negra, Mariposa, Mariposa Parche, Parche Mariposa |
| 25 | *Megachasma pelagios* | Megamouth Shark | 220.1 | Megamouth Shark, Megamouth Shark |
| 26 | *Galeocerdo cuvier* | Tiger Shark | 216.87 | Tiger Shark |
| 27 | *Caranx caninus* | Pacific Crevalle Jack | 203.57 | Pacific Crevalle Jack, Carangue Crevalle, Jiguajua, Aurel, Burel, Burro, Canche Jurel, Chumbo, Cocinero, Jiguagua, Jurel, Jurel Caninus, Jurel Común, Jurelito, Jurel Toro, Sargentillo, Sargento, Toro |
| 28 | *Chiloscyllium indicum* | Catshark | 202.28 | Catshark, Frog Shark, Nurse Shark, Ridgeback Catshark, Ridgebacked Bamboo Shark, Ridge Back Shark, Slender Bamboo Shark, Stone Shark, Time Teller Shark, Requin-chabot Élégant, Bamboa Elegante |
| 29 | *Salmo salar* | Atlantic Salmon | 200.32 | Atlantic Salmon, Salmon |
| 30 | *Chaetodon lunula* | Halfmoon Butterflyfish | 195.99 | Halfmoon Butterflyfish, Moon Butterflyfish, Moon Butterfly fish, Raccoon Butterfly, Raccoon Butterflyfish, Raccoon Butterflyfish , Racoon, Racoon Butterflyfish, Racoon Coralfish, Redstriped Butterflyfish, Chétodon à croissant, Chétodon raton-laveur, Mariposa mapache |
| 31 | *Naso unicornis* | Bluespine Unicornfish | 194.96 | Bluespine Unicornfish, Humphead Unicornfish, Longhorn Unicornfish, Longnose Unicornfish, Long-snouted Unicornfish, Unicorn Tang, Brown Unicornfish, Corne, Dawa, Licorne, Nason à Eperons Bleus, Nason Brun, Nason Vert, Barbero de Aguijón Azul, Berbero de Aguijon Azul |
| 32 | *Chlamydoselachus anguineus* | Frilled Shark | 194.06 | Frilled Shark, Lizard Shark, Scaffold Shark |
| 33 | *Bathyuroconger vicinus* | Large-toothed Conger | 189.06 | Large-toothed Conger, Eel, Conger Dente, Congrio Dentudo |
| 34 | *Acanthurus bahianus* | Ocean Surgeon | 186.09 | Ocean Surgeon, Grey Doctorfish, Barber, Ocean Surgeonfish, Ocean Tang, Ringtail, Shitty Trooper, Doctorfish, Chirurgien Marron, Barbero, Cirujano, Cirujano pardo, Doctor, Médico, Navajero, Navajón, Navajón Pardo, Sangrador, Sangrador Lucio |
| 35 | *Ginglymostoma cirratum* | Nurse Shark | 183.92 | Nurse Shark, Gata, Tiburón-gata |
| 36 | *Sphyrna tudes* | Curry Shark | 180.73 | Curry Shark, Golden Hammerhead, Smalleye Hammerhead Shark, Requin-marteau À Petits Yeux, Cornuda Ojichica, Tiburón |
| 37 | *Naso brevirostris* | Palefin Unicornfish | 169.64 | Palefin Unicornfish, Shortsnout Unicornfish, Brown Unicornfish, Shortnosed Kala, Short-nosed Unicornfish, Shortnose Unicornfish, Longnose Unicornfish, Spotted Unicornfish, Corne, Nasique, Nason à Rostre Court, Nason Pointillé |
| 38 | *Achirus mazatlanus* | Mazatlan Sole | 164.85 | Mazatlan Sole, Pacific Lined Sole, Sole de Mazatlan, Lenguado, Guardaboya Mulata, Lenguadito, Lenguado Redondo, Sol, Sol de Mazatlán, Suela Arepita, Tepalcate |

## S4 Table continued

| Rank | Species | Common names | Average monthly web search interest | All common names |
|---|---|---|---|---|
| 39 | *Polyprion americanus* | Wreckfish | 164.23 | Wreckfish, Bass, Bass Groper, Cherna, Sea Bass, Sea Rock Perch, Stone Bass, Wreck Bass, Atalntic Wreckfish, Wreck-fish, Atlantic Wreckfish, Mérot Gris, Cernia, Cernier, Cernier Atlantique, Cernier Commun, Cernio Escourpena, Franfré Rascas, Lucerna, Mérou, Mérou de Bosques, Mérou Fanfré, Péro-mérot, Peskar Goat, Poisson de Bois, Bacalao, Cherna, Cherne, Chernia, Chernoda, Girom, Jorna, Mero, Mero Chernia, Mero de Roca, Pampol |
| 40 | *Chaenomugil proboscideus* | Mullet | 164.02 | Mullet, Snouted mullet, Snouted mullet (FB), Mulet grande gueule, Lisa, Lisa hocicona |
| 41 | *Haemulon steindachneri* | Chere-chere grunt | 158.4 | Chere-chere grunt, Grunt, Latin grunt, Chivilico, Roncador sol, Ronco chere-chere, Sol |
| 42 | *Prionace glauca* | Blue Shark | 157.31 | Blue Shark |
| 43 | *Leporinus striatus* | Characin | 153.71 | Characin, Striped leporinus, Lisa, Sardina, Trompa colorada |
| 44 | *Boraras maculatus* | Dwarf Rasbora | 153.24 | Dwarf Rasbora, Dwarf-spotted Rasbora, Dwarf, Pygmy Rasbora, Spotted Rasbora |
| 45 | *Epinephelus caninus* | Dogtooth Grouper | 152.94 | Dogtooth Grouper, Merou Nior, Merou Gris, Cherne Denton, Cachorro, Cherne Ley, Mero, Mero Dentol, Mero Denton |
| 46 | *Caulolatilus cyanops* | Blackline Tilefish | 150.31 | Blackline Tilefish, Ocean Whitefish, Whitey, Whitney, Tile à Raie Noire, Blanquilla, Blanquillo, Blanquillo Raya Negra, Domingo, Lismonero, Paleta, Paleta Lista Negra, Tumba |
| 47 | *Platichthys flesus* | Baltic Flounder | 148.5 | Baltic Flounder, Butt, European Flounder, Fluke, Mud Flounder, River Flounder, White Fluke, Flet, Flet d'Europe, Platija, Platija Europea, Platixa, Solla |
| 48 | *Batrachoides surinamensis* | Pacuma Toadfish | 144.53 | Pacuma Toadfish, Sapo, Poisson Crapaud, Crapaud Guyanais, Poisson Guyanais, Sapo Guayanes, Sapo Guayanés |
| 49 | *Mugil cephalus* | Flathead Mullet | 143.29 | Flathead Mullet, Bright Mullet, Bully Mullet, Callifaver Mullet, Common Grey Mullet, Common Mullet, Flathead Greymullet, Flathead Grey Mullet, Black Mullet, Grey Mullet, Haarder, Hardgut Mullet, Mangrove Mullet, Mullet, River Mullet, Sea Mullet, Springer, Black True Mullet, Cabot, Carida, Caridou, Cremole, Meuil, Muge, Muge à Groose Tête, Muge Cabot, Muge Céphale, Mugo Fangous, Mujou, Mulet, Mulet à Grosse Tête, Mulet-cabot, Mulet Cabot, Mulet Jaune, Mulet Jeune, Poisson Queue Bleue, Testard, Testu, Lisa, Albur, Bullûa, Cabeçut, Cabezudo, Cachamba, Capitán, Capiton, Cap Pla, Céfalo, Galupe, Iliça de Cap Gros, Iliça Sabada, Ilissa Lobarrera, Lebranche, Lisa Cabezuda, Lisa Común, Lisa Pardete, Lisa Rayada, Lissa Amaria, Liza Cabezona, Lizarra, Lizza, Machu, Machuto, Mugil, Mugil Común, Mujol, Mule, Muxo, Pardete, Pordete |
| 50 | *Liza aurata* | Golden Grey Mullet | 142.16 | Golden Grey Mullet, Long-finned Grey Mullet, Mulet Doré, Aurin, Galtorous, Gaouto-rouno, Jaouna, Meil, Meuille, Muge Doré, Muge Durin, Mujou-taco, Mulet, Mulet à Tête Fine, Alise, Dabeta, Galtiroig, Galupe, Iliça, Ilissa, Lisa, Lisa Dorada, Lissa Negra, Lizarda, Mule, Muxo Salton |
| 51 | *Mugil incilis* | Parassi Mullet | 141.39 | Parassi Mullet, Striped Mullet, Mullet, Common Mullet, Grey Mullet, Mulet Parassi, Mulet Prassi, Lisa, Lisa Rayada |
| 52 | *Euthynnus lineatus* | Black Skipjack | 140.92 | Black Skipjack, Thonine Noire, Bonito Negro, Atún Patiseca, Barrilete Negro, Bonito, Macarela, Negra, Pataseca, Patiseca |
| 53 | *Xiphias gladius* | Swordfish | 139.74 | Swordfish, Broadbill, Broadbill Swordfish, Espadon, Espadron, Poisson Porte-épée, Albacora, Aja Para, Chichi Spada, Emperador, Espada, Espadon, Espardarte, Pez Espada |

**S4 Table continued**

| Rank | Species | Common names | Average monthly web search interest | All common names |
|---|---|---|---|---|
| 54 | *Mugil hospes* | Hospe Mullet | 138.34 | Hospe Mullet, Mullet, Mulet Hospe, Lisa, Lisa Hospe |
| 55 | *Caranx caballus* | Green Jack | 137.57 | Green Jack, Jack, Carangue Verte, Cocinero, Bonito, Burique, Caballa, Caballa Jurel, Caballo, Cavalleta, Chumbo, Cocinero Dorado, Cojinoa, Cojinua, Cojinua Chata, Dorado, Jurel, Jurel Bonito, Jurel Dorado, Jurel Verde, Palometa Dorada |
| 56 | *Mugil setosus* | Liseta mullet | 134.73 | Liseta mullet, Mulet lesète, Lisa, Lisa liseta |
| 57 | *Heterodontus mexicanus* | Mexican Hornshark | 134.73 | Mexican Hornshark, Requin Dormeur Buffle, Dormilón Búfalo, Perro, Tiburón Comudo, Tiburón Gato |
| 58 | *Apeltes quadracus* | Fourspine Stickleback | 133.55 | Fourspine Stickleback, Spannistickle, Barnytickle, Branchy, Branstickle, Doctor, Four-spined Stickleback, Barnstickle, Prickley, Prickly, Prickly Back, Spanicle, Barnystickle, Spanny, Spannytickle, Spantickle, Sparnicle, Sparny, Sparnytickle, Spawn, Spawnykettle, Spawnytickle, Thornback, Thorny back, Épinoche à Quatre Epines |
| 59 | *Sphoeroides lobatus* | Longnose puffer | 131.74 | Longnose puffer, Longnose puffer (FB), Puffer, Compère à nez long, Bola, Botete narizon, Botete verrugoso, Pez sapo, Tamboreta, Tamboril narigon, Tambulero |
| 60 | *Carcharhinus leucas* | Bull Shark | 130.25 | Bull Shark |
| 61 | *Scomberomorus regalis* | Cero | 128.19 | Cero, Thazard Franc, Thazard Atlantique, Carite Rey, Carito, Sierra |
| 62 | *Somniosus microcephalus* | Greenland Shark | 125.87 | Greenland Shark, Gurry Shark, Large Sleeper Shark, Sleeper, Sleeper Shark, Laimargue Du Groenland, Tiburón Boreal, Tollo De Groenlandia |
| 63 | *Hippocampus guttulatus* | Long-snouted Seahorse | 119.99 | Long-snouted Seahorse, Seahorse, Cheval Marin, Hippocampe Moucheté, Caballito de Mar |
| 64 | *Carcharias taurus* | Sand Tiger | 119.83 | Sand Tiger, Spotted Ragged-tooth Shark, Grey Nurse Shark, Sand Tiger Shark, Grey Nurse Shark, Spotted Raggedtooth Shark, Requin Taureau, Toro Bacota |
| 65 | *Acanthurus coeruleus* | Blue Tang | 119.83 | Blue Tang, Blue Doctorfish, Blue Barber, Blue Tang Surgeonfish, Yellow Barber, Yellow Doctorfish, Blue Doctor, Chirurgien Bayolle, Chirurgien Bleu, Barbero, Barbero azul, Cirujano, Cirujano azul, Doctor, Médico, Navajero, Navajón, Navajón Azul, Sangrador Azul |
| 66 | *Scomberomorus sierra* | Pacific Sierra | 116.94 | Pacific Sierra, Thazard Sierra, Serrucho, Carite Sierra, Macarela, Sierra, Sierra Del Pacifico, Verle |
| 67 | *Alopias pelagicus* | Pelagic Thresher | 114.37 | Pelagic Thresher, Thresher Shark, Whiptail Shark |
| 68 | *Pterotolithus maculatus* | Blotched Tiger-toothed Croaker | 114.16 | Blotched Tiger-toothed Croaker, False Seatrout, Blotched Tigertoothed Croaker, Rainha, Spotted Croaker |
| 69 | *Acanthurus chirurgus* | Doctorfish | 112.25 | Doctorfish, Black Doctorfish, Chirurgien Docteur, Gallinazo, Barbero rayado, Cirujano, Cirujano rayado, Doctor, Navajero, Navajón, Navajón cirujano, Navajón rayado, Sangrador común, Sangrador rayado |
| 70 | *Trachinotus rhodopus* | Gafftopsail Pompano | 109.57 | Gafftopsail Pompano, Pompano, Pompanito, Pompaneau Fin, Pampanillo, Domingo, Palometa, Pampanito, Pámpano, Pámpano Fin, Pampano Fino, Pámpano Fino |
| 71 | *Negaprion brevirostris* | Lemon Shark | 107.35 | Lemon Shark |
| 72 | *Erpetoichthys calabaricus* | Reed | 101.48 | Reed, Reedfish, Sailfin, Snakefish, Snake Fish |
| 73 | *Scomber scombrus* | Atlantic Mackerel | 97.71 | Atlantic Mackerel, Mackerel, Split, Joey, Maquereau Commun, Maquereau, Caballa, Caballa del Atlántico, Maquereau Bleu, Verdel, Xarda |

**S4 Table continued**

| Rank | Species | Common names | Average monthly web search interest | All common names |
|---|---|---|---|---|
| 74 | *Kajikia albida* | White Marlin | 93.23 | White Marlin, Marlin, Skilligalee, Espadon, Makaire Blanc, Makaire Blanc de L'Atlantique, Aguja de Costa, Aguja Blanca, Aguja Blanca del Atlántico, Aguja de Paladar, Alfiler, Alton, Blanca, Cabezona, Cometa, Marlin Blanco, Pez Aguja |
| 75 | *Coris julis* | African rainbow wrasse | 92.25 | African rainbow wrasse, Mediterranean rainbowfish, Mediterranean rainbow wrasse, Rainbow wrasse, Wrasse, Girelle, Doncella, Julia, Xulia |
| 76 | *Anisotremus scapularis* | Chita | 92.14 | Chita, Corcovado, Roncador peruano, Sargo |
| 77 | *Lutjanus colorado* | Vivaneau amarante | 87.81 | Vivaneau amarante, Huachinango, Pargo colorado, Pargo jilguero, Pargo lunarejo, Pargueta, Rojo |
| 78 | *Brachyplatystoma rousseauxii* | Gilded catfish | 86.26 | Gilded catfish, Bagre dorado, Dorado, Plateado, Zúngaro dorado |
| 79 | *Hemigrammus pulcher* | Black wedge tetra | 84.36 | Black wedge tetra, Garnet tetra, Pretty, Pretty tetra, Mojarita, Sardinita, Tetra granate |
| 80 | *Distichodus engycephalus* | Grass-eater | 83.48 | Grass-eater, Grass-eaters, Perch, Perch (FB) |
| 81 | *Distichodus rostratus* | Grass-eater | 82.43 | Grass-eater, Grass-eater (FB), Perch |
| 82 | *Hermosilla azurea* | Perch | 82.14 | Perch, Zebra perch, Zebra- perch sea chub, Chopa azul, Chopa bonita |
| 83 | *Lophius vomerinus* | Cape Monk | 81.62 | Cape Monk, Monk, Devil Anglerfish, Baudroie Diable, Baudroie du Cap, Rape del Cabo, Rape Diablo |
| 84 | *Tautogolabrus adspersus* | Blue perch | 81.47 | Blue perch, Chogset, Conner, Cunner, Perch, Sea perch, Limbert achigan, Tanche-tautogue |
| 85 | *Perca fluviatilis* | Perch | 75.38 | Perch |
| 86 | *Stygnobrotula latebricola* | Black Brotula | 73.94 | Black Brotula, Black Widow, Brótula Oscura, Brótula negra, Latebrícola, Viúva negra |
| 87 | *Coryphaena equiselis* | Pompano Dolphinfish | 72.34 | Pompano Dolphinfish, Mahi Mahi, Coryphène Dauphin, Caméléon, Dorado, Dorado Chato, Dorado Enano |
| 88 | *Opisthonema libertate* | Deep-bodied Pacific thread herring | 69.36 | Deep-bodied Pacific thread herring, Pacific thread herring, Thread herring, Chavelo, Dinamarca, Machete de hebra, Machuelo hebra pinchagua, Pinchagua común, Sardina crinuda |
| 89 | *Heterodontus francisci* | Bullhead Shark | 68.54 | Bullhead Shark, California Horn Shark, Horned Shark, Horn Shark, Port Jackson Shark, Requin Dormeur Cornu, Dormilón Cornudo |
| 90 | *Scomberomorus brasiliensis* | Serra Spanish Mackerel | 66.62 | Serra Spanish Mackerel, Serra, Atlantic Sierra, Carite, Maquereau, Bonite, Thazard Franc, Thazard Serra, Thazard Tacheté Du Sud, Carite Pintado, Serra |
| 91 | *Lile stolifera* | Pacific Piquitinga | 66.34 | Pacific Piquitinga, Striped Herring, Herring, Sardine, Harengule Piquitinga, Harengule Piquitinge Pacifique, Pelada, Sardina, Sardina Rayada, Sardineta, Sardineta Piquitinga Pelada |
| 92 | *Lamna nasus* | Porbeagle | 65.07 | Porbeagle, Requin-taupe Commun, Marrajo Sardinero, Tiburón Sardinero, Tintorera |
| 93 | *Mycteroperca interstitialis* | Crossband Rockfish | 64.93 | Crossband Rockfish, Grey Mannock, Hamlet, Harlequin Rockfish, Princess Rockfish, Rockfish, Salmon Grouper, Salmon Rock Fish, Scamp, Yellowmouth Grouper, Badeche Gueule Jaune, Abadejo, Abadejo Salmon, Bacalao, Badejo, Badejo-amarelo, Blake, Cherna Boca Amarilla, Cuna Amarilla, Cuna Chulinga, Cuna Raba Rajao, Mero |
| 94 | *Acanthocybium solandri* | Wahoo | 63.47 | Wahoo, Mackerel, Queen-fish, Barracuda, Wahoo Fish, Kingfish, Thazard Noir, Poisson Bécune, Thazard-bâtard, Thazard Raité, Thon Banane, Guaho, Guatapaná, Peje Sierra, Peto, Sierra Canalera |

## S4 Table continued

| Rank | Species | Common names | Average monthly web search interest | All common names |
|---|---|---|---|---|
| 95 | *Marcusenius macrolepidotus* | Bulldog | 62.34 | Bulldog, Elephant Nose, Bulldog-fish, Tana-bulldog |
| 96 | *Odontaspis ferox* | Small-tooth Sand Tiger Shark | 61.1 | Small-tooth Sand Tiger Shark, Herbst's Nurse Shark, Sand Shark, Ragged-tooth Shark, Smalltooth Sand Tiger Shark, Requin Feroce, Solrayo |
| 97 | *Mycteroperca olfax* | Colorado Grouper | 57.54 | Colorado Grouper, Mangrove, Sailfin Grouper, Sea Bass, Yellow Grouper, Merou Voile, Bacalao, Cabrillejo, Cherna, Garropa Parda, Mero |
| 98 | *Conodon macrops* | Lemoneye grunt | 56.98 | Lemoneye grunt, Bravo, Limón, Ronco ojón |
| 99 | *Protopterus annectens* | African Lungfish | 56.46 | African Lungfish, Mudfish, Lungfish, Tana Lungfish, West African Lungfish |
| 100 | *Callorhinchus milii* | Elephant Fish | 56.36 | Elephant Fish, Elephant Shark, Ghost Shark, Reperepe, Whitefish |
