# Supplementary material for: Popular interest in vertebrates does not reflect extinction risk and is associated with bias in conservation investment
Source: PLoS One. 2018 Sep 26;13(9):e0203694. doi: 10.1371/journal.pone.0203694 (PMC6157853; doi:10.1371/journal.pone.0203694)
Supplement: S5 Table — Data corresponding to Fig 2e. (PDF) [file pone.0203694.s006.pdf]

S5 Table. The top 52 most Googled amphibians in the world. Data corresponding to Figure 2e. Note that Google Trends only returned data for 52 species that were not omitted from the analysis. The remaining species had insufficient search volume and average monthly web search interest was therefore scored as 0.

Rank	Species	Common names	Average monthly web search interest	All common names
1	*Litoria caerulea*	Green Tree Frog	110.81	Green Tree Frog
2	*Ambystoma tigrinum*	Tiger Salamander	86.37	Tiger Salamander,Salamandra
3	*Ambystoma velasci*	Plateau Tiger Salamander	56.05	Plateau Tiger Salamander,Salamandra
4	*Necturus maculosus*	Mudpuppy	54.97	Mudpuppy
5	*Rhinella marina*	Cane Toad	41.23	Cane Toad,Marine Toad,Giant Toad,Giant Marine Toad,Crapaud,Sapo Común,Sapo Grande
6	*Megophrys nasuta*	Borneon Horned Frog	37.61	Borneon Horned Frog,Horned Toad,Large Horned Frog,Malayan Horned Frog
7	*Conraua goliath*	Giant Slippery Frog	37.33	Giant Slippery Frog,Goliath Frog
8	*Litoria aurea*	Golden Bell Frog	31.19	Golden Bell Frog,Green and Golden Bell Frog,Green and Golden Swamp Frog,Green Frog
9	*Lithobates sylvaticus*	Wood Frog	30.04	Wood Frog
10	*Litoria chloris*	Red-eyed Green Treefrog	28.44	Red-eyed Green Treefrog,Red-eyed Tree Frog,Southern Orange-eyed Tree Frog
11	*Triturus cristatus*	Great Crested Newt	27.51	Great Crested Newt,Northern Crested Newt
12	*Andrias davidianus*	Chinese Giant Salamander	27.19	Chinese Giant Salamander
13	*Pseudacris crucifer*	Spring Peeper	26.93	Spring Peeper
14	*Dyscophus antongilii*	Tomato Frog	24.88	Tomato Frog
15	*Lithobates catesbeianus*	American Bullfrog	24.35	American Bullfrog,Bullfrog,Common Bullfrog,Grenouille-taureau,Rana Toro
16	*Pseudophryne corroboree*	Corroboree Frog	23.89	Corroboree Frog
17	*Hylarana daemeli*	Water Frog	23.51	Water Frog,Wood Frog
18	*Litoria moorei*	Bell Frog	23.22	Bell Frog,Moore's Frog,Motorbike Frog,Western Green And Golden Bell Frog
19	*Hylarana erythraea*	Common Green Frog	22.3	Common Green Frog,Green Paddy Frog,Leaf Frog,Red-eared Frog
20	*Hyla cinerea*	Green Treefrog	22.24	Green Treefrog
21	*Atelopus zeteki*	Golden Arrow Poison Frog	21.85	Golden Arrow Poison Frog,Golden Frog,Panamanian Golden Frog,Zetek's Golden Frog,Rana Dorada
22	*Lithobates clamitans*	Green Frog	21.11	Green Frog
23	*Litoria ewingii*	Brown Tree Frog	20.52	Brown Tree Frog,Ewing's Tree Frog,Southern Brown,Whistling Tree Frog
24	*Pseudotriton ruber*	Red Salamander	20.25	Red Salamander
25	*Lithobates pipiens*	Northern Leopard Frog	18.75	Northern Leopard Frog
26	*Phrynoidis aspera*	Asian Giant Toad	18.29	Asian Giant Toad,River Toad,Rough Toad
27	*Fejervarya limnocharis*	Asian Grass Frog	17.4	Asian Grass Frog,Common Pond Frog,Field Frog,Grass Frog,Indian Rice Frog

S5 Table continued

Rank	Species	Common names	Average monthly web search interest	All common names
28	*Proteus anguinus*	Olm	16.63	Olm,Proteus
29	*Incilius alvarius*	Colorado River Toad	16.44	Colorado River Toad
30	*Phyllobates terribilis*	Golden Poison Frog	15.65	Golden Poison Frog
31	*Salamandra salamandra*	Common Fire Salamander	15.57	Common Fire Salamander,Fire Salamander,Salamandra Común
32	*Physalaemus fischeri*	Oe	15.22	Oe
33	*Cryptobranchus alleganiensis*	Hellbender	15.07	Hellbender
34	*Trichobatrachus robustus*	Hairy Frog	14.65	Hairy Frog
35	*Limnonectes blythii*	Blyth's River Frog	13.92	Blyth's River Frog,Giant Asian River Frog,Giant Frog
36	*Hylarana nicobariensis*	Cricket Frog	13.83	Cricket Frog,Nicobar Cricket Frog,Nicobarese Frog,Nicobar Frog,Nicobar Island Frog
37	*Austrochaperina pluvialis*	Rain Frog	13.78	Rain Frog
38	*Epidalea calamita*	Natterjack Toad	13.71	Natterjack Toad,Sapo Corredor
39	*Agalychnis callidryas*	Red-eyed Treefrog	13.54	Red-eyed Treefrog,Rana Arborícola
40	*Megophrys montana*	Horned Frog	13.54	Horned Frog,Javan Horned Frog
41	*Litoria nasuta*	Rocket Frog	13.39	Rocket Frog
42	*Litoria australis*	Giant Frog	12.6	Giant Frog
43	*Pelophylax lateralis*	Kokarit Frog	12.23	Kokarit Frog,Yellow Frog
44	*Eurycea lucifuga*	Cave Salamander	12.04	Cave Salamander
45	*Aneides flavipunctatus*	Black Salamander	11.47	Black Salamander
46	*Pyxicephalus adspersus*	African Bullfrog	10.59	African Bullfrog
47	*Anaxyrus americanus*	American Toad	10.58	American Toad
48	*Limnodynastes peronii*	Brown Frog	10.13	Brown Frog,Perons Marsh Frog,Striped Marsh Frog
49	*Rhinella schneideri*	Cururu Toad	9.99	Cururu Toad,Cururú,Rococo
50	*Telmatobius niger*	UCO	9.74	UCO
51	*Atelopus varius*	Harlequin Frog	9.59	Harlequin Frog,Rana Arlequin
52	*Bufo bufo*	Common Toad	8.34	Common Toad,Crapaud Commun,Crapaud Vulgaire,Sapo Común
