# Supplementary material for: Popular interest in vertebrates does not reflect extinction risk and is associated with bias in conservation investment
Source: PLoS One. 2018 Sep 26;13(9):e0203694. doi: 10.1371/journal.pone.0203694 (PMC6157853; doi:10.1371/journal.pone.0203694)
Supplement: S6 Table — Data corresponding to Fig 2f. (PDF) [file pone.0203694.s007.pdf]

S6 Table. The top 100 most Googled birds in the world. Data corresponding to Figure 2f

Rank	Species	Common names	Average monthly web serach interest	All common names
1	*Haliaeetus leucocephalus*	Bald Eagle	1670.95	Bald Eagle
2	*Campephilus principalis*	Ivory-billed Woodpecker	1421.69	Ivory-billed Woodpecker
3	*Falco peregrinus*	Peregrine Falcon	1166.65	Peregrine Falcon,Peregrine,Faucon pèlerin
4	*Dacelo novaeguineae*	Laughing Kookaburra	802.86	Laughing Kookaburra,Kookaburra
5	*Coturnix coturnix*	Common Quail	798.47	Common Quail,Quail,Caille des blés
6	*Dromaius novaehollandiae*	Emu	704.38	Emu
7	*Erithacus rubecula*	European Robin	696.85	European Robin,Robin,Rougegorge
8	*Cuculus canorus*	Common Cuckoo	662.36	Common Cuckoo,European Cuckoo,Cuckoo,Coucou gris
9	*Tyto alba*	Barn Owl	606.53	Barn Owl,Chouette effraie
10	*Aptenodytes forsteri*	Emperor Penguin	585.83	Emperor Penguin
11	*Struthio camelus*	Ostrich	501.79	Ostrich,Common Ostrich,Autruche
12	*Pandion haliaetus*	Osprey	355.33	Osprey,Balbuzard pêcheur
13	*Fratercula arctica*	Atlantic Puffin	333.66	Atlantic Puffin,Puffin
14	*Aquila chrysaetos*	Golden Eagle	326.06	Golden Eagle,Aigle royal
15	*Anas platyrhynchos*	Mallard	268.05	Mallard,Northern Mallard,Common Mallard,Canard colvert
16	*Psittirostra psittacea*	Ou	264.78	Ou,'O'u
17	*Harpia harpyja*	Harpy Eagle	254.4	Harpy Eagle,American Harpy Eagle,Aguila Harpía,Aguila Arpía,Arpía,Arpía Mayor,Harpía
18	*Meleagris gallopavo*	Wild Turkey	221.77	Wild Turkey,Turkey
19	*Eudyptula minor*	Little Penguin	217.85	Little Penguin,Fairy Penguin,Blue Penguin
20	*Ardea herodias*	Great Blue Heron	203.08	Great Blue Heron
21	*Bubo virginianus*	Great Horned Owl	190.99	Great Horned Owl
22	*Falco tinnunculus*	Common Kestrel	186.66	Common Kestrel,Kestrel,Eurasian Kestrel,Faucon crécerelle
23	*Accipiter cooperii*	Cooper's Hawk	183.31	Cooper's Hawk
24	*Aptenodytes patagonicus*	King Penguin	178.05	King Penguin,Manchot royal
25	*Strix varia*	Barred Owl	175.16	Barred Owl
26	*Bubo scandiaca*	Snowy Owl	168.87	Snowy Owl
27	*Falco columbarius*	Merlin	165.1	Merlin,Faucon émerillon

S6 Table continued

Rank	Species	Common names	Average monthly web serach interest	All common names
28	*Apus apus*	Common Swift	161.32	Common Swift,Swift,European Swift,Martinet noir
29	*Athene cunicularia*	Burrowing Owl	146.64	Burrowing Owl
30	*Spheniscus demersus*	African Penguin	136.82	African Penguin,Jackass Penguin,Black-footed Penguin,Manchot du Cap,Pingüino del Cabo
31	*Strix occidentalis*	Spotted Owl	136.02	Spotted Owl,Búho Manchado,Cárabo Californiano
32	*Grus grus*	Common Crane	134.17	Common Crane,Crane,Grue cendrée
33	*Garrulus glandarius*	Eurasian Jay	132.62	Eurasian Jay,Jay
34	*Eudyptes chrysolophus*	Macaroni Penguin	122.93	Macaroni Penguin,Gorfou doré
35	*Gymnogyps californianus*	California Condor	115.24	California Condor,Cóndor Californiano,Cóndor de California
36	*Strix nebulosa*	Great Grey Owl	114.02	Great Grey Owl,Great Gray Owl
37	*Pygoscelis adeliae*	Adelie Penguin	110.96	Adelie Penguin
38	*Branta canadensis*	Canada Goose	109.26	Canada Goose
39	*Eudyptes chrysocome*	Southern Rockhopper Penguin	108.9	Southern Rockhopper Penguin,Rockhopper Penguin
40	*Chen caerulescens*	Snow Goose	105.81	Snow Goose
41	*Rhea americana*	Greater Rhea	105.5	Greater Rhea,Lesser Rhea,Common Rhea,Avestruz,Ñandú,Ñandú Común
42	*Accipiter gentilis*	Northern Goshawk	96.78	Northern Goshawk,Eurasian Goshawk,Goshawk,Autour des palombes
43	*Buteo jamaicensis*	Red-tailed Hawk	95.91	Red-tailed Hawk
44	*Dryocopus pileatus*	Pileated Woodpecker	83.33	Pileated Woodpecker
45	*Aix galericulata*	Mandarin Duck	83.07	Mandarin Duck,Mandarin
46	*Colinus virginianus*	Northern Bobwhite	77.91	Northern Bobwhite,Bobwhite Quail,Bobwhite
47	*Falco rusticolus*	Gyr Falcon	77.19	Gyr Falcon,Gyrfalcon
48	*Coracias garrulus*	European Roller	71.16	European Roller,Roller,Rollier d'Europe
49	*Aix sponsa*	Wood Duck	68.84	Wood Duck
50	*Circus cyaneus*	Northern Harrier	68.37	Northern Harrier,Hen Harrier,Busard Saint-Martin
51	*Pavo cristatus*	Indian Peafowl	66.88	Indian Peafowl,Common Peafowl,Peafowl
52	*Micrathene whitneyi*	Elf Owl	66.67	Elf Owl
53	*Pygoscelis papua*	Gentoo Penguin	66.5	Gentoo Penguin
54	*Corvus corax*	Common Raven	64.39	Common Raven,Northern Raven,Raven
55	*Strigops habroptila*	Kakapo	63.27	Kakapo,Owl Parrot,Cacapo

S6 Table continued

Rank	Species	Common names	Average monthly web serach interest	All common names
56	*Pica pica*	Black-billed Magpie	60.87	Black-billed Magpie,Eurasian Magpie,Common Magpie,Magpie
57	*Falco subbuteo*	Eurasian Hobby	59.08	Eurasian Hobby,Hobby,European Hobby,Faucon hobereau
58	*Milvus milvus*	Red Kite	56.62	Red Kite,Milan royal,Milano Real
59	*Turdus migratorius*	American Robin	56.36	American Robin
60	*Picoides pubescens*	Downy Woodpecker	53.67	Downy Woodpecker
61	*Anas crecca*	Common Teal	53.01	Common Teal,Green-winged Teal,Eurasian Teal,Teal,Sarcelle d'hiver
62	*Pygoscelis antarcticus*	Chinstrap Penguin	52.81	Chinstrap Penguin
63	*Phasianus colchicus*	Common Pheasant	52.65	Common Pheasant,Pheasant,Ring-necked Pheasant
64	*Cyanocitta cristata*	Blue Jay	50.6	Blue Jay
65	*Accipiter nisus*	Eurasian Sparrowhawk	49.67	Eurasian Sparrowhawk,Sparrowhawk,European Sparrowhawk,Epervier d'Europe
66	*Megascops asio*	Eastern Screech-owl	48.17	Eastern Screech-owl,Eastern Screech Owl,Eastern Screech-Owl
67	*Alcedo atthis*	Common Kingfisher	47.75	Common Kingfisher,Kingfisher,European Kingfisher,Martin-pêcheur d'Europe
68	*Falco sparverius*	American Kestrel	47.05	American Kestrel
69	*Picoides villosus*	Hairy Woodpecker	46.06	Hairy Woodpecker
70	*Nycticorax nycticorax*	Black-crowned Night-heron	44.97	Black-crowned Night-heron,Black-crowned Night-Heron.,Black-crowned Night-Heron,Black-crowned Night Heron,Night Heron,Héron bihoreau
71	*Glaucidium passerinum*	Eurasian Pygmy-owl	43.44	Eurasian Pygmy-owl,Eurasian Pygmy-Owl,Eurasian Pygmy Owl,Pygmy Owl
72	*Gymnomyza samoensis*	Mao	43.08	Mao
73	*Colaptes auratus*	Northern Flicker	42.92	Northern Flicker,Yellow-shafted Flicker
74	*Sterna paradisaea*	Arctic Tern	42	Arctic Tern,Sterne arctique
75	*Strix aluco*	Tawny Owl	41.61	Tawny Owl
76	*Cairina moschata*	Muscovy Duck	41.29	Muscovy Duck
77	*Spheniscus mendiculus*	Galapagos Penguin	41.29	Galapagos Penguin,Galápagos Penguin
78	*Surnia ulula*	Northern Hawk Owl	40.75	Northern Hawk Owl,Hawk Owl,Northern Hawk-Owl
79	*Cathartes aura*	Turkey Vulture	40.4	Turkey Vulture
80	*Casmerodius albus*	Great Egret	39.53	Great Egret,Great White Heron,Great White Egret,Grande aigrette
81	*Troglodytes troglodytes*	Winter Wren	39.41	Winter Wren,Wren
82	*Cygnus atratus*	Black Swan	38.96	Black Swan
83	*Anas rubripes*	American Black Duck	36.46	American Black Duck,Black Duck

S6 Table continued

Rank	Species	Common names	Average monthly web serach interest	All common names
84	*Bucephala albeola*	Bufflehead	36.46	Bufflehead
85	*Spheniscus humboldti*	Humboldt Penguin	36.3	Humboldt Penguin,Peruvian Penguin,Pingüino de Humboldt
86	*Falco cherrug*	Saker Falcon	33.17	Saker Falcon,Saker,Faucon sacre
87	*Hirundo rustica*	Barn Swallow	32.46	Barn Swallow,Swallow,European Swallow,Hirondelle de cheminée
88	*Ardea cinerea*	Grey Heron	31.41	Grey Heron,Gray Heron,Héron cendré
89	*Eudyptes schlegeli*	Royal Penguin	30.45	Royal Penguin
90	*Egretta thula*	Snowy Egret	30.29	Snowy Egret
91	*Uria aalge*	Common Guillemot	29.89	Common Guillemot,Guillemot,Common Murre,Guillemot de Troïl
92	*Turdus merula*	Eurasian Blackbird	29.87	Eurasian Blackbird,Common Blackbird,Blackbird
93	*Perdix perdix*	Grey Partridge	29.84	Grey Partridge,Gray Partridge,Partridge
94	*Calidris canutus*	Red Knot	29.74	Red Knot,Knot,Lesser Knot,Bécasseau maubèche
95	*Histrionicus histrionicus*	Harlequin Duck	29.43	Harlequin Duck,Harlequin
96	*Buteo regalis*	Ferruginous Hawk	28.82	Ferruginous Hawk
97	*Cardinalis cardinalis*	Northern Cardinal	28.43	Northern Cardinal
98	*Botaurus stellaris*	Great Bittern	28.34	Great Bittern,Eurasian Bittern,Common Bittern,Bittern,Butor étoilé
99	*Numenius arquata*	Eurasian Curlew	28.12	Eurasian Curlew,Curlew,Courlis cendré
100	*Falco mexicanus*	Prairie Falcon	27.95	Prairie Falcon
