# Supplementary material for: Popular interest in vertebrates does not reflect extinction risk and is associated with bias in conservation investment
Source: PLoS One. 2018 Sep 26;13(9):e0203694. doi: 10.1371/journal.pone.0203694 (PMC6157853; doi:10.1371/journal.pone.0203694)
Supplement: S7 Table — (PDF) [file pone.0203694.s008.pdf]

S7 Table. Species of endangered and critically endangered vertebrate that receive low web search interest. Species names are given only. Letters in parenthesis represent extinction risk followed by population trend as given by the IUCN red list of threatened species. Extinction risk: EN=Endangered, CR = Critically Endangered; Population Trend: D = Decreasing, S = Stable, I = Increasing, U = Unknown, blank spaces occur where population trend was not specified.

S7 Table continued

AMPHIBIA: Alsodes montanus (CR, D); Alsodes pehuenche (CR, D); Alsodes tumultuosus (CR, D); Alsodes vanzolinii (CR, D); Eupsophus contulmoensis (EN, U); Eupsophus insularis (CR, U); Eupsophus migueli (EN, D); Eupsophus nahuelbutensis (EN, S); Discoglossus nigriventer (CR, U); Allobates juanii (CR, D); Allobates kingsburyi (EN, D); Allobates mandelorum (EN, D); Allobates ranoides (EN, D); Aromobates alboguttatus (EN, D); Aromobates duranti (EN, D); Aromobates haydeeae (EN, D); Aromobates leopardalis (CR, D); Aromobates mayorgai (EN, U); Aromobates meridensis (CR, D); Aromobates molinarii (EN, U); Aromobates nocturnus (CR, D); Aromobates orostoma (EN, U); Aromobates saltuensis (EN, S); Aromobates serranus (EN, D); Mannophryne caquetio (CR, D); Mannophryne collaris (EN, D); Mannophryne cordilleriana (CR, S); Mannophryne lamarcai (CR, D); Mannophryne leonardoi (EN, D); Mannophryne neblina (CR, D); Mannophryne riveroi (EN, D); Mannophryne trujillensis (EN, D); Mannophryne yustizi (EN, D); Prostherapis dunni (CR, D); Arthroleptis crusculum (EN, D); Arthroleptis fichika (EN, U); Arthroleptis francei (EN, D); Arthroleptis kidogo (CR, U); Arthroleptis krokosua (EN, U); Arthroleptis kutogundua (CR, U); Arthroleptis nikeae (EN, D); Arthroleptis perreti (EN, U); Arthroleptis troglodytes (CR, D); Astylosternus fallax (EN, D); Astylosternus laurenti (EN, D); Astylosternus nganhanus (CR, D); Astylosternus perreti (EN, D); Astylosternus ranoides (EN, D); Astylosternus schioetzi (EN, D); Cardioglossa alsco (CR, D); Cardioglossa aureoli (EN, D); Cardioglossa melanogaster (EN, D); Cardioglossa oreas (EN, D); Cardioglossa pulchra (EN, D); Cardioglossa schioetzi (EN, D); Cardioglossa trifasciata (CR, D); Cardioglossa venusta (EN, D); Leptodactylodon axillaris (CR, U); Leptodactylodon erythrogaster (CR, D); Leptodactylodon mertensi (EN, U); Leptodactylodon ornatus (EN, D); Leptodactylodon perreti (EN, D); Leptodactylodon stevarti (EN, D); Leptodactylodon wildi (EN, D); Leptopelis karissimbensis (EN, D); Leptopelis parkeri (EN, U); Leptopelis susanae (EN, U); Leptopelis uluguruensis (EN, U); Leptopelis xenodactylus (EN, U); Atelognathus patagonicus (EN, D); Atelognathus praebasalticus (EN, D); Atelognathus reverberii (EN, D); Barbourula kalimantanensis (EN, D); Bombina pachypus (EN, D); Balebreviceps hillmani (CR, D); Breviceps sylvestris (EN, U); Callulina hanseni (CR, U); Callulina kanga (CR, U); Callulina kisiwamsitu (EN, D); Callulina laphami (CR, U); Callulina meteora (CR, U); Callulina shengena (CR, U); Callulina stanleyi (CR, U); Probreviceps durirostris (EN, U); Probreviceps loveridgei (EN, S); Probreviceps macrodactylus (EN, U); Probreviceps rhodesianus (EN, D); Probreviceps rungwensis (EN, U); Probreviceps uluguruensis (EN, U); Adenomus dasi (CR, D); Adenomus kandianus (CR, U); Adenomus kelaartii (EN, D); Altiphrynoides malcolmi (EN, D); Amietophrynus brauni (EN, D); Amietophrynus djohongensis (EN, D); Amietophrynus pantherinus (EN, U); Amietophrynus taiensis (CR, D); Amietophrynus villiersi (EN, D); Anaxyrus californicus (EN, D); Anaxyrus canorus (EN, D); Anaxyrus houstonensis (EN, D); Anaxyrus nelsoni (EN, D); Andinophryne colomai (CR, D); Ansonia guibei (EN, D); Ansonia latidisca (EN, D); Ansonia platysoma (EN, D); Atelopus andinus (CR, D); Atelopus angelito (CR, D); Atelopus ardila (CR, D); Atelopus arsyecue (CR, D); Atelopus arthuri (CR, D); Atelopus balios (CR, D); Atelopus bomolochos (CR, D); Atelopus boulengeri (CR, D); Atelopus carauta (CR, D); Atelopus carbonerensis (CR, D); Atelopus carrikeri (CR, U); Atelopus certus (EN, D); Atelopus chiriquiensis (CR, D); Atelopus chirripoensis (CR, U); Atelopus chocoensis (CR, D); Atelopus chrysocorallus (CR, D); Atelopus coynei (CR, D); Atelopus cruciger (CR, D); Atelopus dimorphus (EN, D); Atelopus ebenoides (CR, D); Atelopus elegans (CR, D); Atelopus epikeisthos (CR, D); Atelopus erythropus (CR, D); Atelopus eusebianus (CR, D); Atelopus eusebiodiazi (CR, D); Atelopus exiguus (CR, D); Atelopus famelicus (CR, D); Atelopus farci (CR, D); Atelopus galactogaster (CR, U); Atelopus gigas (CR, U); Atelopus glyphus (CR, D); Atelopus guanujo (CR, D); Atelopus guitarraensis (CR, D); Atelopus halihelos (CR, D); Atelopus laetissimus (CR, D); Atelopus limosus (EN, D); Atelopus longibrachius (EN, D); Atelopus lozanoi (CR, D); Atelopus lynchi (CR, D); Atelopus mandingues (CR, U); Atelopus mindoensis (CR, D); Atelopus minutulus (CR, D); Atelopus mittermeieri (EN, D); Atelopus monohernandezii (CR, D); Atelopus mucubajiensis (CR, D); Atelopus muisca (CR, D); Atelopus nahumae (CR, U); Atelopus nanay (CR, D); Atelopus nepiozomus (CR, U); Atelopus nicefori (CR, U); Atelopus onorei (CR, D); Atelopus orcesi (CR, U); Atelopus oxapampae (EN, U); Atelopus oxyrhynchus (CR, D); Atelopus pachydermus (CR, D); Atelopus pastuso (CR, U); Atelopus patazensis (CR, D); Atelopus pedimarmoratus (CR, U); Atelopus peruensis (CR, U); Atelopus petersi (CR, D); Atelopus petriruizi (CR, U); Atelopus pictiventris (CR, U); Atelopus pinangoi (CR, D); Atelopus planispina (CR, D); Atelopus podocarpus (CR, U); Atelopus pulcher (CR, D); Atelopus pyrodactylus (CR, U); Atelopus quimbaya (CR, U); Atelopus reticulatus (CR, D); Atelopus seminiferus (CR, D); Atelopus senex (CR, D); Atelopus sernai (CR, D); Atelopus simulatus (CR, D); Atelopus sonsonensis (CR, U); Atelopus sorianoi (CR, D); Atelopus subornatus (CR, D); Atelopus tamaense (CR, D); Atelopus walkeri (CR, D); Bufoides meghalayanus (EN, U); Churamiti maridadi (CR, D); Dendrophryniscus carvalhoi (EN, D); Didynamipus sjostedti (EN, D); Duttaphrynus beddomii (EN, D); Duttaphrynus kotagamai (EN, D); Duttaphrynus noellerti (EN, D); Duttaphrynus sumatranus (CR, D); Ghatophryne ornata (EN, D); Incilius cavifrons (EN, D); Incilius cristatus (CR, D); Incilius fastidiosus (CR, D); Incilius gemmifer (EN, D); Incilius holdridgei (CR, U); Incilius ibarrai (EN, D); Incilius leucomyos (EN, D); Incilius peripatetes (CR, D); Incilius perplexus (EN, D); Incilius spiculatus (EN, D); Incilius tacanensis (EN, D); Incilius tutelarius (EN, D); Ingerophrynus claviger (EN, D); Ingerophrynus kumquat (EN, D); Leptophryne cruentata (CR, D); Melanophryniscus admirabilis (CR, D); Melanophryniscus devincenzii (EN, D); Melanophryniscus langonei (CR, U); Melanophryniscus peritus (CR, U); Mertensophryne anotis (EN, D); Mertensophryne howelli (EN, D); Mertensophryne usambarae (EN, D); Nectophrynoides cryptus (EN, D); Nectophrynoides laticeps (EN, D); Nectophrynoides minutus (EN, D); Nectophrynoides paulae (CR, D); Nectophrynoides poyntoni (CR, D); Nectophrynoides pseudotornieri (EN, D); Nectophrynoides vestergaardi (EN, D); Nectophrynoides wendyae (CR, D); Nimbaphrynoides liberiensis (CR, D); Nimbaphrynoides occidentalis (CR, D); Osornophryne antisana (EN, D); Osornophryne guacamayo (EN, D); Osornophryne percrassa (EN, D); Osornophryne puruanta (EN, U); Osornophryne talipes (EN, D); Parapelophryne scalpta (EN, D); Pedostibes tuberculosus (EN, D); Pelophryne albotaeniata (EN, D); Pelophryne api (EN, D); Pelophryne linanitensis (CR, U); Pelophryne murudensis (CR, U); Peltophryne cataulaciceps (EN, D); Peltophryne florentinoi (CR, U); Peltophryne fluviatica (CR, D); Peltophryne fracta (EN, D); Peltophryne lemur (CR, D); Peltophryne longinasus (EN, D); Rhaebo caeruleostictus (EN, D); Rhinella amabilis (CR, D); Rhinella chavin (CR, D); Rhinella chrysophora (EN, D); Rhinella gallardoi (EN, D); Rhinella macrorhina (EN, D); Rhinella nesiotes (EN, D); Rhinella nicefori (EN, D); Rhinella rostrata (CR, D); Rhinella sclerocephala (EN, D); Vandijkophrynus amatolicus (CR, D); Vandijkophrynus inyangae (EN, D); Werneria bambutensis (EN, D); Werneria iboundji (CR, D); Werneria mertensiana (EN, D); Werneria preussi (EN, U); Werneria submontana (EN, D); Werneria tandyi (EN, D); Wolterstorffina chirioi (CR, D); Wolterstorffina mirei (EN, D); Xanthophryne koynayensis (EN, D); Xanthophryne tigerina (CR, D); Telmatobufo bullocki (CR, U); Telmatobufo venustus (EN, U); Centrolene audax (EN, D); Centrolene azulae (EN, D); Centrolene ballux (CR, D); Centrolene fernandoi (EN, D); Centrolene gemmatum (CR, D); Centrolene heloderma (CR, D); Centrolene lynchi (EN, D); Centrolene petrophilum (EN, D); Centrolene pipilatum (EN, D); Cochranella mache (EN, U); Hyalinobatrachium cardiacalyptum (EN, D); Hyalinobatrachium crybetes (CR, D); Hyalinobatrachium esmeralda (EN, D); Hyalinobatrachium guairarepanense (EN, D); Hyalinobatrachium pallidum (EN, D); Hyalinobatrachium pellucidum (EN, D); Nymphargus anomalus (CR, D); Nymphargus laurae (CR, U); Nymphargus luminosus (EN, D); Nymphargus mariae (EN, D); Nymphargus megacheirus (EN, D); Nymphargus puyoensis (EN, U); Rulyrana saxiscandens (EN, D); Platymantis cagayanensis (EN, D); Platymantis hazelae (EN, D); Platymantis insulatus (CR, D); Platymantis lawtoni (EN, D); Platymantis levigatus (EN, D); Platymantis negrosensis (EN, D); Platymantis panayensis (EN, D); Platymantis polillensis (EN, D); Platymantis spelaeus (EN, D); Platymantis subterrestris (EN, D); Platymantis taylori (EN, D); Platymantis vitianus (EN, D); Conraua derooi (CR, D); Atopophrynus syntomopus (CR, D); Bryophryne bustamantei (EN, U); Bryophryne cophites (EN, U); Craugastor anciano (CR, D); Craugastor andi (CR, D); Craugastor angelicus (CR, D); Craugastor aurilegulus (EN, D); Craugastor azueroensis (EN, D); Craugastor catalinae (CR, D); Craugastor charadra (EN, D); Craugastor coffeus (CR, D); Craugastor cruzi (CR, D); Craugastor daryi (EN, D); Craugastor emcelae (CR, D); Craugastor emleni (CR, D); Craugastor epochthidius (CR, D); Craugastor fecundus (CR, D); Craugastor fleischmanni (CR, U); Craugastor glaucus (CR, D); Craugastor greggi (CR, D); Craugastor guerreroensis (CR, D); Craugastor gulosus (EN, U); Craugastor hobartsmithi (EN, D); Craugastor inachus (EN, D); Craugastor laevissimus (EN, D); Craugastor lauraster (EN, D); Craugastor lineatus (CR, D); Craugastor megalotympanum (CR, D); Craugastor merendonensis (CR, D); Craugastor milesi (CR, U); Craugastor montanus (EN, D); Craugastor obesus (EN, D); Craugastor olanchano (CR, D); Craugastor omiltemanus (EN, D); Craugastor omoaensis (CR, D); Craugastor pechorum (EN, D);

S7 Table continued

Craugastor polymniae (CR, D); Craugastor pozo (CR, D); Craugastor punctariolus (EN, D); Craugastor ranoides (CR, D); Craugastor rhyacobatrachus (EN, D); Craugastor sabrinus (EN, D); Craugastor saltuarius (CR, D); Craugastor sandersoni (EN, D); Craugastor silvicola (EN, D); Craugastor spatulatus (EN, D); Craugastor stadelmani (CR, D); Craugastor stuarti (EN, D); Craugastor tabasarae (CR, D); Craugastor taurus (CR, D); Craugastor trachydermus (CR, D); Craugastor uno (EN, D); Craugastor vulcani (EN, D); Geobatrachus walkeri (EN, D); Holoaden bradei (CR, D); Hypodactylus brunneus (EN, D); Hypodactylus elassodiscus (EN, U); Hypodactylus latens (EN, D); Hypodactylus lucida (CR, D); Lynchius parkeri (EN, D); Niceforonia adenobrachia (CR, D); Oreobates pereger (CR, D); Oreobates zongoensis (CR, D); Phrynopus bracki (EN, D); Phrynopus dagmarae (CR, D); Phrynopus heimorum (CR, D); Phrynopus juninensis (CR, D); Phrynopus kauneorum (CR, D); Phrynopus montium (EN, D); Phrynopus tautzorum (CR, S); Pristimantis acerus (EN, D); Pristimantis actinolaimus (EN, D); Pristimantis acutirostris (EN, D); Pristimantis albericoi (CR, D); Pristimantis angustilineatus (EN, D); Pristimantis atratus (EN, D); Pristimantis bacchus (EN, D); Pristimantis balionotus (EN, D); Pristimantis baryecuus (EN, D); Pristimantis bellona (EN, D); Pristimantis bernali (CR, D); Pristimantis cabrerai (EN, D); Pristimantis cacao (EN, D); Pristimantis calcaratus (EN, D); Pristimantis capitonis (EN, D); Pristimantis chrysops (EN, D); Pristimantis colomai (EN, D); Pristimantis cosnipatae (EN, D); Pristimantis cremnobates (EN, D); Pristimantis crenunguis (EN, D); Pristimantis cryophilius (EN, D); Pristimantis cryptomelas (EN, D); Pristimantis degener (EN, D); Pristimantis deinops (EN, D); Pristimantis devillei (EN, D); Pristimantis dissimulatus (EN, D); Pristimantis dorsopictus (EN, D); Pristimantis eugeniae (EN, D); Pristimantis euphronides (EN, D); Pristimantis fallax (EN, D); Pristimantis fasciatus (EN, U); Pristimantis festae (EN, D); Pristimantis fetosus (EN, D); Pristimantis gentryi (EN, D); Pristimantis ginesi (EN, D); Pristimantis gladiator (EN, U); Pristimantis glandulosus (EN, D); Pristimantis hamiotae (CR, D); Pristimantis helvolus (EN, D); Pristimantis hernandezi (EN, D); Pristimantis ignicolor (EN, D); Pristimantis incanus (EN, D); Pristimantis insignitus (EN, D); Pristimantis johannesdei (EN, D); Pristimantis jorgevelosai (EN, D); Pristimantis katoptroides (EN, D); Pristimantis lancinii (EN, D); Pristimantis lemur (EN, D); Pristimantis lichenoides (CR, D); Pristimantis lividus (EN, D); Pristimantis loustes (EN, D); Pristimantis maculosus (EN, D); Pristimantis mars (EN, D); Pristimantis merostictus (EN, D); Pristimantis mnionaetes (EN, D); Pristimantis modipeplus (EN, D); Pristimantis museosus (EN, U); Pristimantis ocreatus (EN, D); Pristimantis orestes (EN, D); Pristimantis paramerus (EN, D); Pristimantis parectatus (EN, D); Pristimantis pastazensis (EN, D); Pristimantis percultus (EN, D); Pristimantis phragmipleuron (CR, D); Pristimantis polychrus (EN, D); Pristimantis prolatus (EN, D); Pristimantis proserpens (EN, D); Pristimantis pteridophilus (EN, D); Pristimantis pycnodermis (EN, D); Pristimantis pyrrhomerus (EN, D); Pristimantis renjiforum (EN, D); Pristimantis rhodoplichus (EN, D); Pristimantis rivasi (EN, U); Pristimantis rubicundus (EN, D); Pristimantis ruthveni (EN, D); Pristimantis scoloblepharus (EN, D); Pristimantis scolodiscus (EN, D); Pristimantis shrevei (EN, D); Pristimantis simonbolivari (EN, D); Pristimantis simonsii (CR, D); Pristimantis simoteriscus (EN, D); Pristimantis siopelus (EN, D); Pristimantis sobetes (EN, D); Pristimantis spilogaster (EN, D); Pristimantis suetus (EN, D); Pristimantis sulculus (EN, D); Pristimantis surdus (EN, D); Pristimantis tenebrionis (EN, D); Pristimantis thymalopsoides (EN, D); Pristimantis torrenticola (CR, D); Pristimantis tribulosus (CR, D); Pristimantis truebae (EN, D); Pristimantis turumiquirensis (EN, D); Pristimantis urichi (EN, D); Pristimantis veletis (CR, D); Pristimantis vidua (EN, D); Pristimantis viridicans (EN, D); Pristimantis zophus (EN, D); Psychrophrynella boettgeri (EN, U); Psychrophrynella guillei (CR, U); Psychrophrynella illimani (CR, U); Psychrophrynella kallawaya (CR, S); Psychrophrynella saltator (CR, S); Psychrophrynella usurpator (EN, D); Strabomantis helonotus (CR, D); Strabomantis ruizi (EN, D); Yunganastes bisignatus (EN, D); Cycloramphus faustoi (CR, U); Insuetophrynus acarpicus (CR, S); Rhinoderma rufum (CR, D); Thoropa lutzi (EN, D); Ameerega ingeri (CR, D); Ameerega planipaleae (CR, D); Colostethus jacobuspetersi (CR, D); Colostethus mertensi (EN, D); Colostethus ruthveni (EN, D); Epipedobates tricolor (EN, D); Excidobates mysteriosus (EN, D); Hyloxalus anthracinus (CR, D); Hyloxalus azureiventris (EN, D); Hyloxalus cevallosi (EN, D); Hyloxalus delatorreae (CR, D); Hyloxalus edwardsi (CR, D); Hyloxalus elachyhistus (EN, D); Hyloxalus ruizi (CR, D); Hyloxalus toachi (EN, D); Hyloxalus vertebralis (CR, D); Minyobates steyermarki (CR, D); Oophaga arborea (EN, U); Oophaga lehmanni (CR, D); Oophaga speciosa (EN, U); Phyllobates vittatus (EN, S); Ranitomeya abdita (CR, D); Ranitomeya bombetes (EN, D); Ranitomeya dorisswansonae (CR, U); Ranitomeya sirensis (EN, D); Ranitomeya summersi (EN, D); Ranitomeya tolimensis (EN, U); Ranitomeya virolinensis (EN, D); Fejervarya nicobariensis (EN, D); Ingerana charlesdarwini (CR, D); Limnonectes arathooni (EN, D); Limnonectes microtympanum (EN, D); Limnonectes namiyei (EN, D); Limnonectes nitidus (EN, D); Minervarya sahyadris (EN, D); Nannophrys marmorata (CR, D); Nannophrys naeyakai (EN, D); Nanorana maculosa (EN, D); Nanorana unculuanus (EN, D); Nanorana yunnanensis (EN, D); Quasipaa boulengeri (EN, D); Quasipaa robertingeri (EN, D); Zakerana greenii (EN, D); Zakerana murthii (CR, D); Zakerana nilagirica (EN, D); Adelophryne maranguapensis (EN, D); Eleutherodactylus acmonis (EN, D); Eleutherodactylus adelus (EN, D); Eleutherodactylus albipes (CR, D); Eleutherodactylus alcoae (EN, U); Eleutherodactylus amadeus (CR, D); Eleutherodactylus amplinympha (EN, S); Eleutherodactylus andrewsi (EN, D); Eleutherodactylus apostates (CR, U); Eleutherodactylus armstrongi (EN, D); Eleutherodactylus auriculatoides (EN, D); Eleutherodactylus bakeri (CR, D); Eleutherodactylus barlagnei (EN, S); Eleutherodactylus bartonsmithi (CR, D); Eleutherodactylus blairhedgesi (CR, D); Eleutherodactylus bresslerae (CR, D); Eleutherodactylus brevirostris (CR, D); Eleutherodactylus caribe (CR, U); Eleutherodactylus casparii (EN, D); Eleutherodactylus cavernicola (CR, U); Eleutherodactylus chlorophenax (CR, D); Eleutherodactylus corona (CR, D); Eleutherodactylus counouspeus (EN, D); Eleutherodactylus cubanus (CR, D); Eleutherodactylus darlingtoni (CR, U); Eleutherodactylus dennisi (EN, U); Eleutherodactylus dilatus (EN, D); Eleutherodactylus diplasius (EN, U); Eleutherodactylus dixoni (CR, D); Eleutherodactylus dolomedes (CR, D); Eleutherodactylus emiliae (EN, D); Eleutherodactylus eneidae (CR, D); Eleutherodactylus etheridgei (EN, S); Eleutherodactylus eunaster (CR, U); Eleutherodactylus fowleri (CR, D); Eleutherodactylus furcyensis (CR, U); Eleutherodactylus fuscus (CR, U); Eleutherodactylus glamyrus (EN, D); Eleutherodactylus glandulifer (CR, U); Eleutherodactylus glanduliferoides (CR, D); Eleutherodactylus glaphycompus (EN, D); Eleutherodactylus grabhami (EN, D); Eleutherodactylus grahami (EN, D); Eleutherodactylus grandis (CR, D); Eleutherodactylus greyi (EN, D); Eleutherodactylus griphus (CR, D); Eleutherodactylus gryllus (EN, D); Eleutherodactylus guanahacabibes (EN, D); Eleutherodactylus gundlachi (EN, S); Eleutherodactylus haitianus (EN, D); Eleutherodactylus hedricki (EN, D); Eleutherodactylus heminota (EN, D); Eleutherodactylus hypostenor (EN, D); Eleutherodactylus iberia (CR, D); Eleutherodactylus intermedius (EN, U); Eleutherodactylus ionthus (EN, S); Eleutherodactylus jamaicensis (EN, D); Eleutherodactylus jasperi (CR, D); Eleutherodactylus jaumei (CR, D); Eleutherodactylus juanariveroi (CR, U); Eleutherodactylus jugans (CR, D); Eleutherodactylus junori (CR, D); Eleutherodactylus karlschmidti (CR, D); Eleutherodactylus klinikowskii (EN, D); Eleutherodactylus lamprotes (CR, D); Eleutherodactylus leberi (EN, D); Eleutherodactylus lentus (EN, D); Eleutherodactylus leoncei (CR, U); Eleutherodactylus locustus (CR, D); Eleutherodactylus lucioi (CR, D); Eleutherodactylus luteolus (EN, D); Eleutherodactylus mariposa (CR, D); Eleutherodactylus melacara (EN, D); Eleutherodactylus michaelschmidi (EN, U); Eleutherodactylus minutus (EN, D); Eleutherodactylus montanus (EN, D); Eleutherodactylus nortoni (CR, D); Eleutherodactylus notidodes (EN, D); Eleutherodactylus nubicola (EN, D); Eleutherodactylus orcutti (CR, D); Eleutherodactylus orientalis (CR, D); Eleutherodactylus oxyrhyncus (CR, U); Eleutherodactylus parabates (CR, U); Eleutherodactylus parapelates (CR, U); Eleutherodactylus patriciae (EN, D); Eleutherodactylus paulsoni (CR, D); Eleutherodactylus pezopetrus (CR, D); Eleutherodactylus pinarensis (EN, D); Eleutherodactylus pinchoni (EN, D); Eleutherodactylus pituinus (EN, D); Eleutherodactylus poolei (CR, U); Eleutherodactylus portoricensis (EN, D); Eleutherodactylus principalis (EN, D); Eleutherodactylus probolaeus (EN, D); Eleutherodactylus rhodesi (CR, U); Eleutherodactylus richmondi (CR, D); Eleutherodactylus rivularis (CR, D); Eleutherodactylus rufescens (CR, D); Eleutherodactylus rufifemoralis (CR, U); Eleutherodactylus ruthae (EN, D); Eleutherodactylus saxatilis (EN, D); Eleutherodactylus schmidti (CR, D); Eleutherodactylus schwartzi (EN, D); Eleutherodactylus sciagraphus (CR, U); Eleutherodactylus semipalmatus (CR, D); Eleutherodactylus simulans (EN, D); Eleutherodactylus sisyphodemus (CR, D); Eleutherodactylus sommeri (EN, U); Eleutherodactylus symingtoni (CR, D); Eleutherodactylus syristes (EN, D); Eleutherodactylus tetajulia (CR, D); Eleutherodactylus thomasi (EN, D); Eleutherodactylus thorectes (CR, U); Eleutherodactylus toa (EN, D); Eleutherodactylus tonyi (CR, D); Eleutherodactylus turquinensis (CR, D); Eleutherodactylus ventrilineatus (CR, U); Eleutherodactylus warreni (CR, U); Eleutherodactylus wightmanae (EN, D); Eleutherodactylus zeus (EN, D); Eleutherodactylus zugi (EN, D); Heleophryne hewitti (EN, U); Heleophryne rosei (CR, S); Cryptobatrachus boulengeri (EN, D); Cryptobatrachus nicefori (CR, D); Flectonotus fitzgeraldi (EN, D); Gastrotheca bufona (EN, D); Gastrotheca christiani (EN, D); Gastrotheca cornuta (EN, D); Gastrotheca espeletia (EN, D); Gastrotheca lauzuricae (CR, D); Gastrotheca litonedis (EN, D); Gastrotheca orophylax (EN, D); Gastrotheca ovifera (EN, D); Gastrotheca pseustes (EN, D); Gastrotheca psychrophila (EN, D); Gastrotheca riobambae (EN, D); Gastrotheca ruizi (EN, D); Gastrotheca splendens (EN, D); Gastrotheca stictopleura (EN, D); Gastrotheca trachyceps (EN, D); Gastrotheca zeugocystis (CR, D); Hemiphractus johnsoni (EN, D); Agalychnis annae (EN, D); Agalychnis moreletii (CR, D); Argenteohyla siemersi (EN, D); Bokermannohyla izecksohni (CR, D); Bromeliohyla bromeliacia (EN, D); Bromeliohyla dendroscarta (CR, D); Charadrahyla altipotens (CR, D); Charadrahyla chaneque (EN, D); Charadrahyla trux (CR, D); Dendropsophus amicorum (CR, D); Dendropsophus gryllatus (EN, D); Dendropsophus meridensis (EN, D); Duellmanohyla

S7 Table continued

chamulae (EN, D); Duellmanohyla ignicolor (EN, D); Duellmanohyla lythrodes (EN, D); Duellmanohyla salvavida (CR, D); Duellmanohyla soralia (CR, D); Duellmanohyla uranochroa (EN, I); Ecnomiohyla echinata (CR, D); Ecnomiohyla fimbrimembra (EN, D); Ecnomiohyla minera (EN, D); Ecnomiohyla phantasmagoria (EN, D); Ecnomiohyla rabborum (CR, D); Ecnomiohyla salvaje (CR, D); Ecnomiohyla valancifer (CR, D); Exerodonta catracha (EN, D); Exerodonta chimalapa (EN, D); Exerodonta perkinsi (CR, D); Hyla bocourti (CR, D); Hyla heinzsteinitzi (CR, D); Hylomantis lemur (CR, D); Hyloscirtus charazani (EN, D); Hyloscirtus chlorosteus (CR, D); Hyloscirtus colymba (CR, D); Hyloscirtus denticulentus (EN, D); Hyloscirtus lynchi (EN, D); Hyloscirtus pantostictus (EN, D); Hyloscirtus piceigularis (EN, D); Hyloscirtus psarolaimus (EN, U); Hyloscirtus ptychodactylus (CR, D); Hyloscirtus simmonsi (EN, D); Hyloscirtus staufferorum (EN, D); Hypsiboas cymbalum (CR, D); Isthmohyla angustilineata (CR, D); Isthmohyla calypsa (CR, D); Isthmohyla debilis (CR, D); Isthmohyla graceae (CR, D); Isthmohyla insolita (CR, D); Isthmohyla pictipes (EN, D); Isthmohyla rivularis (CR, D); Isthmohyla tica (CR, U); Litoria booroolongensis (CR, D); Litoria brevipalmata (EN, D); Litoria castanea (CR, U); Litoria cooloolensis (EN, D); Litoria dayi (EN, D); Litoria lorica (CR, D); Litoria myola (CR, D); Litoria nannotis (EN, S); Litoria nyakalensis (CR, D); Litoria piperata (CR, U); Litoria raniformis (EN, D); Litoria rheocola (EN, D); Litoria spenceri (CR, D); Megastomatohyla mixe (CR, D); Megastomatohyla mixomaculata (EN, D); Megastomatohyla nubicola (EN, D); Megastomatohyla pellita (CR, D); Osteopilus crucialis (EN, D); Osteopilus marianae (EN, D); Osteopilus wilderi (EN, D); Phyllomedusa ayeaye (CR, U); Phyllomedusa baltea (EN, D); Phyllomedusa ecuatoriana (EN, D); Phytotriades auratus (CR, D); Plectrohyla acanthodes (CR, D); Plectrohyla arborescandens (EN, D); Plectrohyla avia (CR, D); Plectrohyla calthula (CR, D); Plectrohyla calvicollina (CR, D); Plectrohyla celata (CR, D); Plectrohyla cembra (CR, D); Plectrohyla charadricola (EN, D); Plectrohyla chryses (CR, D); Plectrohyla chrysopleura (CR, D); Plectrohyla crassa (CR, D); Plectrohyla cyanomma (CR, D); Plectrohyla cyclada (EN, D); Plectrohyla dasypus (CR, D); Plectrohyla ephemera (CR, D); Plectrohyla exquisita (CR, D); Plectrohyla glandulosa (EN, D); Plectrohyla guatemalensis (CR, D); Plectrohyla hartwegi (CR, D); Plectrohyla hazelae (CR, D); Plectrohyla ixil (CR, D); Plectrohyla lacertosa (EN, D); Plectrohyla mykter (EN, D); Plectrohyla pachyderma (CR, D); Plectrohyla pentheter (EN, D); Plectrohyla pokomchi (CR, D); Plectrohyla psarosema (CR, D); Plectrohyla psiloderma (EN, U); Plectrohyla pycnochila (CR, D); Plectrohyla quecchi (CR, D); Plectrohyla robertsorum (EN, D); Plectrohyla sabrina (CR, D); Plectrohyla sagorum (EN, U); Plectrohyla siopela (CR, D); Plectrohyla tecunumani (CR, D); Plectrohyla teuchestes (CR, D); Plectrohyla thorectes (CR, D); Ptychohyla dendrophasma (CR, D); Ptychohyla erythromma (EN, D); Ptychohyla hypomykter (CR, D); Ptychohyla legleri (EN, D); Ptychohyla leonhardschultzei (EN, D); Ptychohyla macrotympanum (CR, D); Ptychohyla panchoi (EN, D); Ptychohyla salvadorensis (EN, D); Ptychohyla sanctaecrucis (CR, D); Ptychohyla spinipollex (EN, D); Scinax alcatraz (CR, D); Scinax belloni (EN, U); Scinax faivovichi (CR, S); Scinax peixotoi (CR, U); Smilisca dentata (EN, D); Afrixalus clarkei (EN, U); Afrixalus knysnae (EN, U); Afrixalus lacteus (EN, D); Afrixalus sylvaticus (EN, D); Afrixalus uluguruensis (EN, D); Alexteroon jynx (CR, D); Arlequinus krebsi (EN, D); Hyperolius bobirensis (EN, D); Hyperolius dintelmanni (EN, D); Hyperolius kihangensis (EN, D); Hyperolius leleupi (EN, D); Hyperolius leucotaenius (EN, D); Hyperolius nienokouensis (EN, D); Hyperolius nimbae (EN, D); Hyperolius pickersgilli (CR, U); Hyperolius puncticulatus (EN, D); Hyperolius rubrovermiculatus (EN, D); Hyperolius tannerorum (EN, D); Hyperolius thomensis (EN, D); Hyperolius torrentis (EN, D); Hyperolius watsonae (CR, D); Kassina jozani (EN, D); Leiopelma archeyi (CR, D); Leiopelma hamiltoni (EN, S); Leptodactylus fallax (CR, D); Leptodactylus magistris (CR, D); Leptodactylus silvanimbus (CR, D); Physalaemus soaresi (EN, D); Pleurodema somuncurensis (CR, D); Philoria frosti (CR, D); Philoria kundagungan (EN, D); Philoria loveridgei (EN, D); Philoria pughi (EN, D); Philoria richmondensis (EN, D); Philoria sphagnicolus (EN, D); Aglyptodactylus laticeps (EN, D); Boehmantis microtympanum (EN, D); Boophis tampoka (EN, D); Boophis williamsi (CR, D); Gephyromantis azzurrae (EN, D); Gephyromantis corvus (EN, D); Gephyromantis horridus (EN, D); Gephyromantis runewsweeki (EN, D); Gephyromantis silvanus (EN, D); Gephyromantis webbi (EN, D); Mantella aurantiaca (CR, D); Mantella bernhardi (EN, D); Mantella cowanii (CR, D); Mantella crocea (EN, D); Mantella expectata (EN, D); Mantella milotympanum (CR, D); Mantella viridis (EN, D); Mantidactylus madecassus (EN, D); Mantidactylus pauliani (CR, D); Spinomantis brunae (EN, D); Spinomantis guibei (EN, D); Spinomantis microtis (EN, D); Leptobrachella palmata (CR, D); Leptobrachium boringii (EN, D); Leptobrachium echinatum (EN, D); Leptobrachium leishanense (EN, D); Leptolalax alpinus (EN, D); Megophrys ligayae (EN, D); Oreolalax chuanbeiensis (EN, D); Oreolalax liangbeiensis (CR, D); Oreolalax omeimontis (EN, D); Oreolalax pingii (EN, D); Oreolalax puxiongensis (EN, D); Scutiger chintingensis (EN, D); Scutiger maculatus (CR, D); Scutiger muliensis (EN, D); Scutiger ningshanensis (EN, D); Scutiger pingwuensis (EN, D); Xenophrys brachykolos (EN, D); Micrixalus gadgili (EN, D); Micrixalus kottigeharensis (CR, D); Albericus siegfriedi (CR, D); Anodonthyla hutchisoni (EN, D); Anodonthyla rouxae (EN, U); Anodonthyla vallani (CR, U); Callulops kopsteini (EN, D); Chiasmocleis carvalhoi (EN, D); Cophixalus concinnus (CR, S); Cophixalus mcdonaldi (EN, U); Cophixalus monticola (EN, U); Cophixalus neglectus (EN, D); Cophyla berara (CR, D); Hoplophryne rogersi (EN, U); Hoplophryne uluguruensis (EN, U); Kalophrynus palmatissimus (EN, D); Madecassophryne truebae (EN, D); Melanobatrachus indicus (EN, D); Melanophryne carpish (EN, U); Microhyla karunaratnei (CR, D); Microhyla sholigari (EN, D); Microhyla zeylanica (EN, D); Micryletta steinegeri (EN, D); Oreophryne monticola (EN, D); Parhoplophryne usambarica (CR, D); Platypelis alticola (EN, D); Platypelis mavomavo (EN, D); Platypelis milloti (EN, D); Platypelis tetra (EN, D); Plethodontohyla brevipes (EN, D); Plethodontohyla fonetana (EN, U); Ramanella mormorata (EN, D); Ramanella palmata (EN, D); Rhombophryne guentherpetersi (EN, D); Scaphiophryne boribory (EN, D); Scaphiophryne gottlebei (EN, D); Stumpffia helenae (CR, D); Geocrinia alba (CR, D); Mixophyes fleayi (EN, D); Mixophyes iteratus (EN, D); Pseudophryne covacevichae (EN, D); Pseudophryne pengilleyi (EN, D); Taudactylus acutirostris (CR, D); Taudactylus eungellensis (CR, D); Taudactylus pleione (CR, D); Taudactylus rheophilus (CR, U); Nasikabatrachus sahyadrensis (EN, D); Nyctibatrachus aliciae (EN, D); Nyctibatrachus beddomii (EN, D); Nyctibatrachus dattatreyaensis (CR, U); Nyctibatrachus karnatakaensis (EN, U); Nyctibatrachus minor (EN, D); Nyctibatrachus sanctipalustris (EN, D); Nyctibatrachus vasanthi (EN, D); Proceratophrys moratoi (CR, D); Pelobates varaldii (EN, D); Petropedetes dutoiti (CR, D); Petropedetes martiensseni (EN, U); Petropedetes palmipes (EN, D); Petropedetes perreti (EN, D); Petropedetes yakusini (EN, U); Phrynobatrachus annulatus (EN, D); Phrynobatrachus chukuchuku (CR, U); Phrynobatrachus ghanensis (EN, D); Phrynobatrachus intermedius (CR, U); Phrynobatrachus irangi (EN, D); Phrynobatrachus krefftii (EN, U); Phrynobatrachus pakenhami (EN, D); Phrynobatrachus ungujae (EN, D); Pipa myersi (EN, U); Xenopus gilli (EN, U); Xenopus itombwensis (CR, S); Xenopus longipes (CR, S); Ptychadena broadleyi (EN, D); Ptychadena nana (EN, S); Ptychadena newtoni (EN, D); Amietia inyangae (EN, D); Amietia johnstoni (EN, D); Anhydrophryne ngongoniensis (EN, S); Anhydrophryne rattrayi (EN, D); Arthroleptella rugosa (CR, D); Arthroleptella subvoce (EN, U); Ericabatrachus baleensis (CR, U); Microbatrachella capensis (CR, D); Natalobatrachus bonebergi (EN, D); Nothophryne broadleyi (EN, D); Amolops hainanensis (EN, D); Amolops hongkongensis (EN, D); Babina holsti (EN, D); Babina okinavana (EN, D); Babina subaspera (EN, D); Glandirana minima (CR, D); Hylarana asperrima (EN, D); Hylarana mangyanum (EN, D); Hylarana occidentalis (EN, D); Lithobates chichicuahutla (CR, D); Lithobates dunni (EN, D); Lithobates johni (EN, D); Lithobates omiltemanus (CR, D); Lithobates onca (EN, D); Lithobates pueblae (CR, D); Lithobates sevosus (CR, D); Lithobates subaquavocalis (CR, D); Lithobates tlaloci (CR, D); Odorrana amamiensis (EN, D); Odorrana ishikawae (EN, D); Odorrana kuangwuensis (EN, D); Odorrana narina (EN, D); Odorrana supranarina (EN, D); Odorrana utsunomiyaorum (EN, D); Odorrana wuchuanensis (CR, D); Pelophylax cerigensis (CR, D); Pelophylax cretensis (EN, D); Pelophylax shqipericus (EN, D); Pelophylax tenggerensis (EN, D); Rana chevronta (CR, D); Rana holtzi (CR, D); Rana muscosa (EN, D); Rana pyrenaica (EN, D); Rana sauteri (EN, D); Rana sierrae (EN, D); Rana tavasensis (EN, D); Indirana brachytarsus (EN, D); Indirana diplosticta (EN, D); Indirana gundia (CR, D); Indirana leptodactyla (EN, D); Indirana phrynoderma (CR, D); Ghatixalus variabilis (EN, S); Gracixalus quyeti (EN, U); Liuixalus ocellatus (EN, D); Liuixalus romeri (EN, D); Philautus aurantium (EN, D); Philautus disgregus (EN, D); Philautus jacobsoni (CR, D); Philautus kerangae (EN, D); Philautus neelanethrus (EN, D); Philautus sanctisilvaticus (CR, D); Philautus schmackeri (EN, D); Philautus similis (EN, D); Philautus surrufus (EN, D); Polypedates eques (EN, D); Polypedates fastigo (CR, D); Polypedates insularis (EN, U); Polypedates longinasus (EN, D); Pseudophilautus alto (EN, D); Pseudophilautus amboli (CR, D); Pseudophilautus asankai (EN, D); Pseudophilautus auratus (EN, D); Pseudophilautus caeruleus (EN, D); Pseudophilautus cavirostris (EN, D); Pseudophilautus cuspis (EN, D); Pseudophilautus decoris (EN, D); Pseudophilautus femoralis (EN, D); Pseudophilautus folicola (EN, D); Pseudophilautus frankenbergi (EN, D); Pseudophilautus fulvus (EN, D); Pseudophilautus hoffmanni (EN, D); Pseudophilautus limbus (CR, D); Pseudophilautus lunatus (CR, D); Pseudophilautus macropus (CR, D); Pseudophilautus microtympanum (EN, D); Pseudophilautus mittermeieri (EN, D); Pseudophilautus mooreorum (EN, D); Pseudophilautus nemus (CR, D); Pseudophilautus ocularis (EN, D); Pseudophilautus papillosus (CR, D); Pseudophilautus pleurotaenia (EN, D); Pseudophilautus poppiae (EN, D); Pseudophilautus procax (CR, D); Pseudophilautus reticulatus (EN, D); Pseudophilautus sarasinorum (EN, D); Pseudophilautus schmarda (EN, D); Pseudophilautus silus (EN, D); Pseudophilautus silvaticus (EN, D); Pseudophilautus simba (CR, D); Pseudophilautus singu (EN, U); Pseudophilautus steineri (EN, D); Pseudophilautus stuarti (EN, D); Pseudophilautus tanu (EN, U); Pseudophilautus wynaadensis (EN, D); Pseudophilautus zorro (EN, D); Raorchestes chalazodes (CR, D); Raorchestes charius (EN, D); Raorchestes chlorosomma (CR, U); Raorchestes griet (CR, D);

S7 Table continued

Raorchestes kaikatti (CR, D); Raorchestes marki (CR, D); Raorchestes munnarensis (CR, D); Raorchestes nerostagona (EN, D); Raorchestes ponmudi (CR, D); Raorchestes resplendens (CR, D); Raorchestes shillongensis (CR, D); Raorchestes signatus (EN, D); Raorchestes sushili (CR, U); Raorchestes tinniens (EN, D); Raorchestes viridis (EN, D); Rhacophorus angulirostris (EN, D); Rhacophorus arvalis (EN, D); Rhacophorus aurantiventris (EN, D); Rhacophorus calcadensis (EN, D); Rhacophorus lateralis (EN, D); Rhacophorus minimus (EN, D); Rhacophorus pseudomalabaricus (CR, D); Rhacophorus yaoshanensis (EN, D); Theloderma bicolor (EN, U); Sechellophryne gardineri (EN, D); Sechellophryne pipilodryas (CR, U); Sooglossus sechellensis (EN, D); Sooglossus thomasseti (CR, U); Telmatobius atacamensis (CR, D); Telmatobius brachydactylus (EN, D); Telmatobius brevipes (EN, D); Telmatobius brevirostris (EN, D); Telmatobius ceiorum (EN, D); Telmatobius cirrhacelis (CR, D); Telmatobius colanensis (EN, U); Telmatobius culeus (CR, D); Telmatobius degener (EN, U); Telmatobius edaphonastes (EN, D); Telmatobius espadai (CR, D); Telmatobius gigas (CR, D); Telmatobius hypselocephalus (EN, D); Telmatobius ignavus (EN, D); Telmatobius laticeps (EN, D); Telmatobius latirostris (EN, D); Telmatobius macrostomus (EN, D); Telmatobius mayoloi (EN, D); Telmatobius necopinus (EN, D); Telmatobius pefauri (CR, D); Telmatobius pisanoi (EN, D); Telmatobius platycephalus (EN, D); Telmatobius punctatus (CR, D); Telmatobius schreiteri (EN, D); Telmatobius scrocchii (EN, D); Telmatobius sibiricus (EN, D); Telmatobius stephani (EN, D); Telmatobius thompsoni (EN, U); Telmatobius truebae (EN, U); Telmatobius vellardi (CR, D); Telmatobius zapahuirensis (CR, D); Ambystoma altamirani (EN, D); Ambystoma amblycephalum (CR, D); Ambystoma andersoni (CR, D); Ambystoma dumerilii (CR, D); Ambystoma granulosum (CR, D); Ambystoma leorae (CR, D); Ambystoma lermaense (CR, D); Ambystoma ordinarium (EN, D); Ambystoma taylori (CR, U); Batrachuperus londongensis (EN, D); Hynobius abei (CR, D); Hynobius amjiensis (CR, D); Hynobius chinensis (EN, D); Hynobius dunni (EN, D); Hynobius formosanus (EN, D); Hynobius hidamontanus (EN, D); Hynobius okiensis (CR, D); Hynobius sonani (EN, D); Hynobius takedai (EN, D); Hynobius yangi (EN, D); Pachyhynobius yunanicus (EN, D); Paradactylodon gorganensis (CR, D); Paradactylodon mustersi (CR, D); Ranodon sibiricus (EN, D); Batrachoseps campi (EN, D); Bolitoglossa alvaradoi (EN, D); Bolitoglossa capitana (CR, D); Bolitoglossa carri (CR, D); Bolitoglossa celaque (EN, D); Bolitoglossa compacta (EN, D); Bolitoglossa conanti (EN, D); Bolitoglossa decora (CR, D); Bolitoglossa diaphora (CR, D); Bolitoglossa dunni (EN, D); Bolitoglossa engelhardti (EN, D); Bolitoglossa flavimembris (EN, D); Bolitoglossa flaviventris (EN, D); Bolitoglossa franklini (EN, D); Bolitoglossa heiroreias (EN, U); Bolitoglossa longissima (CR, D); Bolitoglossa magnifica (EN, D); Bolitoglossa marmorea (EN, D); Bolitoglossa meliana (EN, D); Bolitoglossa minutula (EN, D); Bolitoglossa nigrescens (EN, D); Bolitoglossa odonnelli (EN, D); Bolitoglossa oresbia (CR, D); Bolitoglossa pandi (EN, D); Bolitoglossa porrasorum (EN, D); Bolitoglossa riletti (EN, D); Bolitoglossa salvinii (EN, D); Bolitoglossa sooyorum (EN, D); Bolitoglossa spongai (EN, D); Bolitoglossa subpalmata (EN, D); Bolitoglossa synoria (CR, U); Bolitoglossa tica (EN, D); Bolitoglossa veracrucis (EN, D); Bradytriton silus (CR, D); Chiropterotriton arboreus (CR, D); Chiropterotriton chiropterus (CR, D); Chiropterotriton chondrostega (EN, D); Chiropterotriton cracens (EN, D); Chiropterotriton dimidiatus (EN, D); Chiropterotriton lavae (CR, U); Chiropterotriton magnipes (CR, D); Chiropterotriton multidentatus (EN, D); Chiropterotriton terrestris (CR, U); Cryptotriton adelos (EN, D); Cryptotriton alvarezdeltoroi (EN, D); Cryptotriton monzoni (CR, D); Cryptotriton nasalis (EN, S); Cryptotriton veraepacis (CR, D); Cryptotriton wakei (CR, D); Dendrotriton bromeliacius (CR, D); Dendrotriton chujorum (CR, S); Dendrotriton cuchumatanus (CR, D); Dendrotriton kekchiorum (EN, U); Dendrotriton rabbi (CR, S); Eurycea naufragia (EN, D); Eurycea tonkawae (EN, D); Gyrinophilus gulolineatus (EN, D); Gyrinophilus subterraneus (EN, U); Nototriton barbouri (EN, D); Nototriton brodiei (CR, D); Nototriton lignicola (CR, D); Nototriton limnospectator (EN, D); Nototriton major (CR, D); Nyctanolis pernix (EN, D); Oedipina altura (CR, D); Oedipina carablanca (EN, U); Oedipina gephyra (EN, D); Oedipina gracilis (EN, D); Oedipina grandis (EN, D); Oedipina maritima (CR, D); Oedipina paucidentata (CR, D); Oedipina poelzi (EN, D); Oedipina pseudouniformis (EN, D); Oedipina stenopodia (EN, D); Oedipina tomasi (CR, D); Parvimolge townsendi (CR, D); Phaeognathus hubrichti (EN, D); Plethodon stormi (EN, D); Plethodon welleri (EN, D); Pseudoeurycea ahuitzotl (CR, D); Pseudoeurycea altamontana (EN, D); Pseudoeurycea anitae (CR, U); Pseudoeurycea aquatica (CR, D); Pseudoeurycea brunnata (CR, D); Pseudoeurycea cochranae (EN, D); Pseudoeurycea conanti (EN, U); Pseudoeurycea exspectata (CR, D); Pseudoeurycea firscheini (EN, D); Pseudoeurycea gadovii (EN, D); Pseudoeurycea gigantea (CR, D); Pseudoeurycea goebeli (CR, D); Pseudoeurycea juarezi (CR, D); Pseudoeurycea lineola (EN, D); Pseudoeurycea longicauda (EN, D); Pseudoeurycea lynchi (CR, D); Pseudoeurycea melanomolga (EN, D); Pseudoeurycea mystax (EN, D); Pseudoeurycea naucampatepetl (CR, D); Pseudoeurycea nigra (CR, D); Pseudoeurycea nigromaculata (CR, D); Pseudoeurycea orchileucos (EN, D); Pseudoeurycea orchimelas (EN, D); Pseudoeurycea parva (CR, D); Pseudoeurycea praecellens (CR, D); Pseudoeurycea rex (CR, D); Pseudoeurycea robertsi (CR, S); Pseudoeurycea saltator (CR, D); Pseudoeurycea smithi (CR, D); Pseudoeurycea tenchalli (EN, D); Pseudoeurycea teotepec (EN, D); Pseudoeurycea tlahcuiloh (CR, D); Pseudoeurycea unguidentis (CR, D); Pseudoeurycea werleri (EN, D); Speleomantes supramontis (EN, D); Thorius arboreus (EN, D); Thorius aureus (CR, D); Thorius boreas (EN, D); Thorius dubitus (EN, D); Thorius grandis (EN, D); Thorius infernalis (CR, D); Thorius lunaris (EN, D); Thorius magnipes (CR, D); Thorius minutissimus (CR, D); Thorius minydemus (CR, U); Thorius munificus (CR, U); Thorius narismagnus (CR, D); Thorius narisovalis (CR, D); Thorius omiltemi (EN, D); Thorius papaloae (EN, D); Thorius pennatulus (CR, D); Thorius pulmonaris (EN, D); Thorius schmidti (EN, D); Thorius smithi (CR, U); Thorius spilogaster (CR, D); Thorius troglodytes (EN, D); Necturus alabamensis (EN, D); Calotriton arnoldi (CR, D); Cynops ensicauda (EN, D); Cynops orphicus (EN, D); Echinotriton andersoni (EN, D); Echinotriton chinhaiensis (CR, D); Euproctus platycephalus (EN, D); Lyciasalamandra antalyana (EN, D); Lyciasalamandra atifi (EN, S); Lyciasalamandra billae (CR, D); Lyciasalamandra fazilae (EN, S); Lyciasalamandra flavimembris (EN, D); Neurergus kaiseri (CR, D); Neurergus microspilotus (CR, D); Notophthalmus meridionalis (EN, D); Paramesotriton guanxiensis (EN, D); Pleurodeles poireti (EN, D); Tylototriton hainanensis (EN, D); Boulengerula changamwensis (EN, U); Boulengerula niedeni (EN, U); Boulengerula taitana (EN, D); Grandisonia brevis (EN, U); Praslinia cooperi (EN, U)

AVES: Anas bernieri (EN, D); Anas chlorotis (EN, I); Anas laysanensis (CR, I); Anas melleri (EN, D); Anas nesiotis (EN, I); Anas wyvilliana (EN, D); Aythya baeri (CR, D); Aythya innotata (CR, S); Branta ruficollis (EN, D); Cairina scutulata (EN, D); Melanitta fusca (EN, D); Mergus octosetaceus (CR, D); Mergus squamatus (EN, D); Oxyura leucocephala (EN, D); Rhodonessa caryophyllacea (CR, U); Tadorna cristata (CR, U); Collocalia bartschi (EN, D); Aglaeactis aliciae (EN, D); Aglaiocercus berlepschi (EN, D); Amazilia boucardi (EN, D); Amazilia castaneiventris (EN, D); Amazilia luciae (EN, D); Campylopterus phainopeplus (EN, D); Chaetocercus berlepschi (EN, D); Coeligena orina (CR, D); Eriocnemis godini (CR, U); Eriocnemis isabellae (CR, D); Eriocnemis mirabilis (CR, D); Eriocnemis nigrivestis (CR, D); Eulidia yarrellii (EN, D); Eupherusa cyanophrys (EN, D); Glaucis dohrnii (EN, D); Heliangelus regalis (EN, D); Hylonympha macrocerca (EN, D); Lepidopyga lilliae (CR, D); Loddigesia mirabilis (EN, D); Lophornis brachylophus (CR, D); Metallura baroni (EN, D); Metallura iracunda (EN, D); Ramphomicron dorsale (EN, D); Selasphorus ardens (EN, D); Sephanoides fernandensis (CR, D); Taphrolesbia griseiventris (EN, D); Aegotheles savesi (CR, D); Caprimulgus noctitherus (EN, D); Caprimulgus prigoginei (EN, D); Eleothreptus candicans (EN, D); Siphonorhis americana (CR, U); Brachyramphus brevirostris (CR, D); Charadrius obscurus (EN, I); Charadrius sanctaehelenae (CR, I); Thinornis novaeseelandiae (EN, I); Vanellus gregarius (CR, D); Vanellus macropterus (CR, U); Rhinoptilus bitorquatus (CR, D); Haematopus chathamensis (EN, I); Larus bulleri (EN, D); Sterna acuticauda (EN, D); Sterna albostriata (EN, D); Sterna bernsteini (CR, D); Sterna lorata (EN, D); Pedionomus torquatus (EN, D); Himantopus novaezelandiae (CR, I); Rostratula australis (EN, D); Eurynorhynchus pygmeus (CR, D); Numenius borealis (CR, U); Numenius tenuirostris (CR, D); Prosobonia cancellata (EN, D); Scolopax rochussenii (EN, D); Tringa guttifer (EN, D); Ardea humbloti (EN, D); Ardea insignis (CR, D); Ardeola idae (EN, D); Botaurus poiciloptilus (EN, D); Gorsachius goisagi (EN, D); Gorsachius magnificus (EN, D); Ciconia boyciana (EN, D); Ciconia stormi (EN, D); Leptoptilos dubius (EN, D); Mycteria cinerea (EN, D); Bostrychia bocagei (CR, D); Nipponia nippon (EN, I); Platalea minor (EN, S); Pseudibis davisoni (CR, D); Thaumatibis gigantea (CR, D); Threskiornis bernieri (EN, D); Claravis geoffroyi (CR, D); Columba argentina (CR, D); Columba thomensis (EN, D); Columbina cyanopis (CR, D); Didunculus strigirostris (EN, D); Ducula aurorae (EN, I); Ducula cineracea (EN, D); Ducula galeata (EN, I); Ducula mindorensis (EN, D); Gallicolumba erythroptera (CR, D); Gallicolumba hoedtii (EN, D); Gallicolumba keayi (CR, D); Gallicolumba menagei (CR, D); Gallicolumba platenae (CR, D); Gallicolumba sanctaecrucis (EN, D); Geotrygon carrikeri (EN, D); Leptotila conoveri (EN, D); Leptotila wellsi (CR, D); Nesoenas mayeri (EN, D); Phapitreron cinereiceps (EN, D); Ptilinopus arcanus (CR, D); Ptilinopus roseicapilla (EN, D); Starnoenas cyanocephala (EN, D); Treron psittaceus (EN, D); Todiramphus gambieri (CR, D); Todiramphus godeffroyi (CR, D); Aceros narcondami (EN, S); Aceros waldeni (CR, D); Anthracoceros montani (CR, D); Penelopides mindorensis (EN, D); Penelopides panini (EN, D); Carpococcyx viridis (CR, D); Centropus steerii (CR, D); Coccyzus rufigularis (EN, D); Neomorphus radiolosus (EN, D); Tauraco bannermani (EN, D); Accipiter gundlachi (EN, D); Buteo ridgwayi (CR, D); Chondrohierax wilsonii (CR, D); Circus maillardi (EN, D); Eutriorchis astur (EN, D); Gyps africanus (EN, D); Gyps bengalensis (CR, D); Gyps indicus (CR, D); Gyps rueppellii (EN, D); Gyps tenuirostris (CR, D); Haliaeetus vociferoides (CR, D); Leptodon forbesi (CR, D); Leucopternis occidentalis (EN, D); Necrosyrtes monachus (EN, D); Nisaetus bartelsi (EN, D);

S7 Table continued

Nisaetus floris (CR, D); Sarcogyps calvus (CR, D); Crax alberti (CR, D); Crax blumenbachii (EN, D); Crax globulosa (EN, D); Oreophasis derbianus (EN, D); Pauxi pauxi (EN, D); Pauxi unicornis (EN, D); Penelope albipennis (CR, D); Penelope ortoni (EN, D); Penelope perspicax (EN, D); Pipile jacutinga (EN, D); Pipile pipile (CR, D); Aepypodius bruijnii (EN, D); Megapodius laperouse (EN, D); Megapodius pritchardii (EN, D); Odontophorus strophium (EN, D); Arborophila rufipectus (EN, D); Centrocercus minimus (EN, D); Francolinus camerunensis (EN, D); Francolinus nahani (EN, D); Francolinus ochropectus (CR, D); Francolinus swierstrai (EN, D); Lophura edwardsi (CR, D); Lophura hatinhensis (EN, D); Ophrysia superciliosa (CR, U); Pavo muticus (EN, D); Perdicula manipurensis (EN, D); Polyplectron katsumatae (EN, D); Polyplectron schleiermacheri (EN, D); Xenoperdix udzungwensis (EN, S); Balearica regulorum (EN, D); Grus japonensis (EN, D); Heliopais personatus (EN, D); Houbaropsis bengalensis (CR, D); Neotis ludwigii (EN, D); Sypheotides indicus (EN, D); Psophia viridis (EN, D); Amaurornis olivieri (EN, D); Cyanolimnas cerverai (CR, D); Gallinula pacifica (CR, U); Gallinula silvestris (CR, U); Gallirallus lafresnayanus (CR, U); Gallirallus okinawae (EN, D); Gallirallus sylvestris (EN, S); Gymnocrex talaudensis (EN, D); Laterallus levraudi (EN, D); Laterallus tuerosi (EN, D); Rallus semiplumbeus (EN, D); Rallus wetmorei (EN, D); Sarothrura ayresi (CR, D); Sarothrura watersi (EN, D); Turnix olivii (EN, D); Mohoua ochrocephala (EN, D); Alauda razae (CR, S); Heteromirafra archeri (CR, D); Heteromirafra sidamoensis (CR, D); Mirafra ashi (EN, D); Spizocorys fringillaris (EN, D); Atrichornis clamosus (EN, D); Atrichornis rufescens (EN, D); Coracina newtoni (CR, D); Amaurospiza carrizalensis (CR, D); Habia atrimaxillaris (EN, D); Apalis flavigularis (EN, D); Apalis fuscigularis (CR, D); Cisticola aberdare (EN, D); Colluricincla sanghirensis (CR, D); Cissa thalassina (CR, D); Corvus florensis (EN, D); Corvus kubaryi (CR, D); Corvus unicolor (CR, D); Zavattariornis stresemanni (EN, D); Calyptura cristata (CR, D); Carpodectes antoniae (EN, D); Cephalopterus glabricollis (EN, D); Cotinga maculata (EN, D); Lipaugus weberi (EN, D); Pachyramphus spodiurus (EN, D); Phibalura boliviana (EN, D); Phytotoma raimondii (EN, D); Xipholena atropurpurea (EN, D); Dasyornis brachypterus (EN, D); Dasyornis longirostris (EN, D); Dicaeum quadricolor (CR, D); Dicrurus fuscipennis (EN, S); Dicrurus menagei (EN, D); Atlapetes blancae (CR, U); Atlapetes flaviceps (EN, D); Atlapetes melanopsis (EN, D); Atlapetes pallidiceps (EN, I); Camarhynchus heliobates (CR, D); Camarhynchus pauper (CR, D); Emberiza aureola (EN, D); Emberiza jankowskii (EN, D); Gubernatrix cristata (EN, D); Melanospiza richardsoni (EN, D); Nesospiza wilkinsi (EN, S); Poospiza alticola (EN, D); Poospiza rubecula (EN, D); Rowettia goughensis (CR, D); Spizella wortheni (EN, D); Sporophila melanops (CR, U); Sporophila palustris (EN, D); Torreornis inexpectata (EN, D); Xenospiza baileyi (EN, D); Grallaria chthonia (CR, D); Grallaria fenwickorum (CR, D); Grallaria kaestneri (EN, D); Grallaria ridgelyi (EN, D); Grallaricula ochraceifrons (EN, D); Carduelis johannis (EN, D); Hemignathus lucidus (CR, U); Hemignathus munroi (EN, D); Loxia megaplaga (EN, D); Loxioides bailleui (CR, D); Loxops caeruleirostris (CR, D); Loxops coccineus (EN, D); Melamprosops phaeosoma (CR, D); Neospiza concolor (CR, D); Oreomystis bairdi (CR, D); Oreomystis mana (EN, D); Palmeria dolei (CR, D); Paroreomyza maculata (CR, U); Paroreomyza montana (EN, D); Pseudonestor xanthophrys (CR, D); Pyrrhula murina (EN, S); Serinus flavigula (EN, D); Telespiza ultima (CR, S); Aphrastura masafuerae (CR, S); Asthenes perijana (EN, D); Cinclodes aricomae (CR, D); Cinclodes palliatus (CR, D); Cranioleuca henricae (EN, D); Cranioleuca muelleri (EN, D); Leptasthenura xenothorax (EN, D); Philydor novaesi (CR, D); Synallaxis infuscata (EN, D); Synallaxis kollari (CR, D); Synallaxis maranonica (CR, D); Synallaxis tithys (EN, D); Synallaxis zimmeri (EN, D); Eurochelidon sirintarae (CR, D); Progne modesta (EN, D); Tachycineta cyaneoviridis (EN, D); Agelaius tricolor (EN, D); Agelaius xanthomus (EN, D); Cacicus koepckeae (EN, D); Curaeus forbesi (EN, D); Icterus northropi (CR, D); Icterus oberi (CR, S); Macroagelaius subalaris (EN, D); Nesopsar nigerrimus (EN, D); Psarocolius cassini (EN, D); Lanius newtoni (CR, D); Laniarius amboimensis (EN, D); Laniarius brauni (EN, D); Malaconotus alius (CR, S); Prionops gabela (EN, D); Telophorus kupeensis (EN, D); Stipiturus mallee (EN, D); Gymnomyza aubryana (CR, D); Manorina melanotis (EN, D); Mimus graysoni (CR, S); Mimus melanotis (EN, D); Mimus trifasciatus (CR, S); Ramphocinclus brachyurus (EN, D); Toxostoma guttatum (CR, D); Chasiempis ibidis (EN, D); Clytorhynchus sanctaecrucis (EN, D); Eutrichomyias rowleyi (CR, D); Metabolus rugensis (EN, D); Monarcha boanensis (CR, D); Monarcha brehmii (EN, D); Monarcha everetti (EN, D); Monarcha sacerdotum (EN, D); Pomarea mendozae (EN, S); Pomarea mira (CR, U); Pomarea nigra (CR, I); Pomarea whitneyi (CR, D); Terpsiphone corvina (CR, I); Anthus sokokensis (EN, D); Macronyx sharpei (EN, D); Copsychus cebuensis (EN, D); Copsychus sechellarum (EN, I); Cyornis ruckii (CR, U); Cyornis sanfordi (EN, D); Ficedula bonthaina (EN, D); Humblotia flavirostris (EN, D); Luscinia ruficeps (EN, D); Monticola erythronotus (EN, D); Myiomela albiventris (EN, D); Myiomela major (EN, D); Rhinomyias albigularis (EN, D); Sheppardia aurantiithorax (EN, D); Sheppardia gabela (EN, D); Sheppardia montana (EN, D); Aethopyga duyvenbodei (EN, D); Anthreptes pallidigaster (EN, D); Nectarinia loveridgei (EN, D); Oriolus isabellae (CR, D); Oriolus mellianus (EN, D); Pardalotus quadragintus (EN, D); Basileuterus griseiceps (EN, D); Catharopeza bishopi (EN, D); Dendroica chrysoparia (EN, D); Geothlypis beldingi (EN, D); Geothlypis speciosa (EN, D); Leucopeza semperi (CR, U); Myioborus pariae (EN, D); Vermivora bachmanii (CR, U); Antilophia bokermanni (CR, D); Pitta gurneyi (EN, D); Pitta superba (EN, D); Platysteira laticincta (EN, D); Foudia rubra (EN, S); Malimbus ballmanni (EN, D); Malimbus ibadanensis (EN, D); Ploceus aureonucha (EN, D); Ploceus batesi (EN, D); Ploceus golandi (EN, D); Ploceus nicolli (EN, D); Polioptila clementsi (CR, D); Chlorocichla prigoginei (EN, D); Ixos siquijorensis (EN, D); Phyllastrephus leucolepis (CR, D); Eleoscytalopus psychopompus (CR, D); Merulaxis stresemanni (CR, D); Scytalopus canus (EN, D); Scytalopus iraiensis (EN, D); Scytalopus robbinsi (EN, D); Scytalopus rodriguezi (EN, D); Sitta ledanti (EN, D); Sitta magna (EN, D); Sitta victoriae (EN, D); Aplonis brunneicapillus (EN, D); Aplonis pelzelni (CR, D); Leucopsar rothschildi (CR, D); Sturnus melanopterus (CR, D); Acrocephalus aequinoctialis (EN, D); Acrocephalus brevipennis (EN, D); Acrocephalus caffer (EN, D); Acrocephalus familiaris (CR, S); Acrocephalus griseldis (EN, D); Acrocephalus luscinius (CR, D); Acrocephalus sorghophilus (EN, D); Acrocephalus vaughani (EN, D); Artisornis moreaui (CR, D); Bradypterus graueri (EN, D); Eremomela turneri (EN, D); Hyliota usambara (EN, D); Macrosphenus pulitzeri (EN, D); Trichocichla rufa (EN, S); Cercomacra carbonaria (CR, D); Clytoctantes alixii (EN, D); Formicivora erythronotos (EN, D); Formicivora littoralis (EN, D); Herpsilochmus parkeri (EN, D); Myrmeciza ruficauda (EN, D); Myrmotherula fluminensis (CR, D); Myrmotherula snowi (CR, D); Pyriglena atra (EN, D); Rhopornis ardesiacus (EN, D); Stymphalornis acutirostris (EN, D); Terenura sharpei (EN, D); Terenura sicki (EN, D); Bangsia aureocincta (EN, D); Buthraupis aureodorsalis (EN, D); Compsospiza garleppi (EN, D); Conothraupis mesoleuca (CR, D); Diglossa gloriosissima (EN, D); Diglossa venezuelensis (EN, D); Nemosia rourei (CR, D); Tangara cabanisi (EN, D); Crocias langbianis (EN, D); Dasycrotapha speciosa (EN, D); Garrulax courtoisi (CR, D); Garrulax rufifrons (EN, D); Garrulax yersini (EN, D); Kupeornis gilberti (EN, D); Rimator pasquieri (EN, D); Stachyris nigrorum (EN, D); Strophocincla cachinnans (EN, D); Cistothorus apolinari (EN, D); Ferminia cerverai (EN, D); Henicorhina negreti (CR, D); Thryothorus nicefori (CR, D); Troglodytes monticola (CR, D); Alethe choloensis (EN, D); Myadestes lanaiensis (CR, U); Myadestes palmeri (CR, I); Myophonus blighi (EN, D); Turdus helleri (CR, D); Turdus swalesi (EN, D); Turdus xanthorhynchus (CR, D); Zoothera guttata (EN, D); Anairetes alpinus (EN, D); Hemitriccus kaempferi (EN, D); Myiarchus semirufus (EN, D); Myiotheretes pernix (EN, D); Phyllomyias urichi (EN, D); Phylloscartes beckeri (EN, D); Phylloscartes ceciliae (EN, D); Phylloscartes lanyoni (EN, D); Phylloscartes roquettei (EN, D); Poecilotriccus luluae (EN, D); Tyrannus cubensis (EN, D); Xenopirostris damii (EN, D); Vireo masteri (EN, D); Cleptornis marchei (CR, D); Madanga ruficollis (EN, D); Rukia ruki (EN, D); Zosterops albogularis (CR, D); Zosterops chloronothus (CR, D); Zosterops conspicillatus (EN, D); Zosterops luteirostris (EN, D); Zosterops modestus (EN, I); Zosterops nehrkorni (CR, D); Zosterops rotensis (CR, D); Fregata andrewsi (CR, D); Phalacrocorax capensis (EN, D); Phalacrocorax featherstoni (EN, D); Phalacrocorax neglectus (EN, D); Phalacrocorax onslowi (CR, D); Papasula abbotti (EN, D); Celeus obrieni (EN, D); Dendrocopos noguchii (CR, D); Picumnus steindachneri (EN, D); Picumnus varzeae (EN, S); Aulacorhynchus huallagae (EN, D); Podiceps gallardoi (CR, D); Podiceps taczanowskii (CR, D); Rollandia microptera (EN, D); Diomedea amsterdamensis (CR, D); Diomedea dabbenena (CR, D); Diomedea sanfordi (EN, D); Phoebastria irrorata (CR, D); Phoebetria fusca (EN, D); Thalassarche carteri (EN, D); Thalassarche chlororhynchos (EN, D); Thalassarche chrysostoma (EN, D); Nesofregetta fuliginosa (EN, D); Oceanites maorianus (CR, U); Oceanodroma homochroa (EN, D); Oceanodroma macrodactyla (CR, U); Pelecanoides garnotii (EN, D); Pseudobulweria aterrima (CR, D); Pseudobulweria becki (CR, D); Pseudobulweria macgillivrayi (CR, D); Pterodroma alba (EN, D); Pterodroma atrata (EN, D); Pterodroma axillaris (EN, I); Pterodroma baraui (EN, D); Pterodroma cahow (EN, I); Pterodroma caribbaea (CR, U); Pterodroma hasitata (EN, D); Pterodroma incerta (EN, D); Pterodroma madeira (EN, S); Pterodroma magentae (CR, I); Pterodroma phaeopygia (CR, D); Puffinus auricularis (CR, D); Puffinus huttoni (EN, S); Puffinus mauretanicus (CR, D); Puffinus newelli (EN, D); Amazona imperialis (EN, I); Amazona oratrix (EN, D); Amazona rhodocorytha (EN, D); Amazona vinacea (EN, D); Amazona viridigenalis (EN, D); Amazona vittata (CR, I); Anodorhynchus glaucus (CR, U); Anodorhynchus leari (EN, U); Ara ambiguus (EN, D); Ara glaucogularis (CR, S); Ara rubrogenys (EN, D); Aratinga brevipes (EN, D); Aratinga solstitialis (EN, D); Brotogeris pyrrhoptera (EN, D); Cacatua haematuropygia (CR, D); Cacatua sulphurea (CR, D); Calyptorhynchus baudinii (EN, D); Calyptorhynchus latirostris (EN, D); Charmosyna amabilis (CR, D); Charmosyna diadema (CR, U); Charmosyna toxopei (CR, D); Cyanopsitta spixii (CR, U); Cyanoramphus cookii (CR, D); Cyanoramphus forbesi (EN, S); Cyanoramphus malherbi (CR, D); Eos histrio (EN, D); Eunymphicus uvaeensis (EN, I); Hapalopsittaca fuertesi (CR, D); Loriculus flosculus (EN, D); Lorius domicella (EN, D); Neophema chrysogaster (CR, D); Ognorhynchus icterotis (EN, I); Prioniturus verticalis (CR, D); Psephotus chrysopterygius (EN, D); Psittacula eques (EN, I); Pyrrhura

S7 Table continued

griseipectus (CR, D); Pyrrhura orcesi (EN, D); Pyrrhura pfrimeri (EN, D); Pyrrhura viridicata (EN, D); Rhynchopsitta pachyrhyncha (EN, D); Rhynchopsitta terrisi (EN, D); Touit melanonotus (EN, D); Vini kuhlii (EN, D); Vini ultramarina (EN, D); Eudyptes moseleyi (EN, D); Eudyptes sclateri (EN, D); Megadyptes antipodes (EN, D); Glaucidium mooreorum (CR, D); Heteroglaux blewitti (CR, D); Ketupa blakistoni (EN, D); Otus alfredi (EN, D); Otus beccarii (EN, D); Otus capnodes (CR, D); Otus insularis (EN, S); Otus ireneae (EN, D); Otus moheliensis (CR, D); Otus pauliani (CR, D); Otus siaoensis (CR, U); Otus thilohoffmanni (EN, D); Xenoglaux loweryi (EN, D); Phodilus prigoginei (EN, D); Tyto nigrobrunnea (EN, D); Apteryx mantelli (EN, D); Apalharpactes reinwardtii (EN, D)

FISH: Acipenser baerii (EN, D); Acipenser dabryanus (CR, D); Acipenser gueldenstaedtii (CR, D); Acipenser mikadoi (CR, D); Acipenser naccarii (CR, D); Acipenser nudiventris (CR, D); Acipenser persicus (CR, D); Acipenser schrenckii (CR, D); Acipenser sinensis (CR, D); Acipenser stellatus (CR, D); Acipenser sturio (CR, D); Huso dauricus (CR, D); Pseudoscaphirhynchus fedtschenkoi (CR, U); Pseudoscaphirhynchus hermanni (CR, D); Pseudoscaphirhynchus kaufmanni (CR, D); Scaphirhynchus albus (EN, D); Scaphirhynchus suttkusi (CR, D); Anguilla anguilla (CR, D); Craterocephalus fluviatilis (EN,); Poblana alchichica (CR,); Poblana letholepis (EN,); Poblana squamata (EN,); Teramulus waterloti (EN, D); Atherinella jiloaensis (CR, D); Chirostoma attenuatum (EN, D); Chirostoma promelas (EN, D); Colpichthys hubbsi (EN, U); Bedotia sp nov 'Manombo' (CR, D); Bedotia sp nov 'Sambava' (CR, D); Bedotia sp nov 'Vevembe' (CR, D); Bedotia tricolor (CR, U); Chilatherina sentaniensis (CR, U); Glossolepis wanamensis (CR, U); Melanotaenia boesemani (EN, U); Neostethus thessa (EN, U); Kiunga ballochi (CR, D); Pseudomugil mellis (EN,); Scaturiginichthys vermeilipinnis (CR,); Adrianichthys kruyti (CR,); Oryzias orthognathus (EN,); Xenopoecilus oophorus (EN,); Xenopoecilus poptae (CR,); Xenopoecilus sarasinorum (EN,); Nomorhamphus towoetii (EN, U); Carcharhinus borneensis (EN, U); Carcharhinus hemiodon (CR, U); Glyphis garricki (CR, D); Glyphis siamensis (CR, U); Isogomphodon oxyrhynchus (CR, D); Lamiopsis temmincki (EN, D); Haploblepharus kistnasamyi (CR, U); Holohalaelurus favus (EN, D); Holohalaelurus punctatus (EN, D); Hemitriakis leucoperiptera (EN, U); Mustelus fasciatus (CR, D); Mustelus schmitti (EN, D); Alestes bouboni (EN, U); Alestopetersius nigropterus (EN, U); Brycinus bartoni (EN, U); Brycinus jacksonii (EN, D); Ladigesia roloffi (EN, U); Rhabdalestes leleupi (CR, D); Gymnocharacinus bergii (EN,); Nannocharax altus (EN, U); Neolebias axelrodi (EN, U); Neolebias kerguennae (EN, U); Neolebias lozii (CR, U); Neolebias powelli (CR, U); Alosa killarnensis (CR, D); Alosa vistonica (CR, U); Alosa volgensis (EN, U); Clupeonella abrau (CR, D); Nannothrissa stewarti (EN, U); Barbatula eregliensis (CR, D); Barbatula samantica (EN, D); Barbatula tschaiyssuensis (EN, D); Homaloptera montana (EN, U); Homaloptera santhamparaiensis (EN, U); Longischistura striatus (EN, U); Mesonoemacheilus herrei (CR, D); Nemacheilus dori (CR, S); Nemacheilus jordanicus (EN, D); Nemacheilus pantheroides (EN, D); Nemacheilus petrubanarescui (EN, U); Nemacheilus pulchellus (EN, D); Nemacheilus sp nov (EN, U); Nemacheilus troglocataractus (CR, D); Nemachilichthys shimogensis (EN, U); Oxynoemacheilus seyhanensis (EN, D); Oxynoemacheilus simavica (CR, D); Schistura bairdi (EN, U); Schistura bolavenensis (EN, U); Schistura kangjupkhulensis (EN, D); Schistura leukensis (CR, U); Schistura minutus (EN, U); Schistura nagodiensis (EN, U); Schistura nasifilis (CR, D); Schistura nudidorsum (EN, U); Schistura papulifera (CR, U); Schistura pridii (EN, U); Schistura quasimodo (EN, D); Schistura reticulata (EN, U); Schistura sijuensis (EN, U); Schistura spiloptera (CR, D); Schistura tenura (CR, D); Schistura thanho (EN, D); Schistura tigrinum (EN, U); Sewellia albisuera (CR, D); Sewellia breviventralis (CR, D); Sewellia marmorata (EN, D); Sewellia patella (EN, D); Sewellia pterolineata (EN, D); Sphaerophysa dianchiensis (CR, U); Travancoria elongata (EN, U); Travancoria jonesi (EN, U); Yunnanilus discoloris (CR, D); Yunnanilus nigromaculatus (EN, D); Catostomus microps (EN,); Chasmistes brevirostris (EN, D); Chasmistes cujus (CR,); Chasmistes liorus (CR, D); Deltistes luxatus (EN, D); Moxostoma robustum (EN, U); Xyrauchen texanus (CR, D); Botia striata (EN, U); Cobitis arachthosensis (EN, U); Cobitis bilseli (CR, U); Cobitis calderoni (EN, D); Cobitis hellenica (EN, U); Cobitis illyrica (CR, U); Cobitis jadovaensis (CR, U); Cobitis puncticulata (CR, D); Cobitis stephanidisi (CR, U); Cobitis taurica (CR, U); Cobitis trichonica (EN, U); Cobitis turcica (EN, D); Cobitis vettonica (EN, D); Lepidocephalichthys arunachalensis (EN, U); Lepidocephalichthys jonklaasi (EN,); Paralepidocephalus yui (EN, U); Yasuhikotakia sidthimunki (EN, D); Aaptosyax grypus (CR, D); Acanthobrama centisquama (EN, D); Acheilognathus elongatus (CR, U); Achondrostoma occidentale (EN, U); Alburnus macedonicus (CR, D); Alburnus mandrensis (CR, U); Alburnus mentoides (EN, U); Alburnus orontis (EN, D); Alburnus sarmaticus (EN, U); Alburnus schischkovi (EN, U); Alburnus vistonicus (CR, U); Alburnus volviticus (EN, D); Anabarilius alburnops (EN, D); Anabarilius andersoni (CR, D); Anabarilius polylepis (EN, D); Anabarilius qiluensis (CR, D); Anabarilius yangzonensis (CR, D); Anaecypris hispanica (EN, D); Aulopyge huegelii (EN, D); Balantiocheilos ambusticauda (CR, D); Bangana decorus (CR, D); Barbodes bovanicus (CR, U); Barbodes wynaadensis (CR, D); Barbus acuticeps (EN, D); Barbus aliciae (EN, U); Barbus andrewi (EN, D); Barbus bawkuensis (EN, U); Barbus boboi (CR, U); Barbus bourdariei (EN, U); Barbus caninus (EN, D); Barbus carcharhinoides (CR, U); Barbus claudinae (EN, U); Barbus erubescens (CR, D); Barbus euboicus (CR, S); Barbus huguenyi (EN, U); Barbus lauzannei (EN, U); Barbus liberiensis (EN, U); Barbus melanotaenia (CR, U); Barbus nigroluteus (EN, U); Barbus quadralineatus (EN, D); Barbus ruasae (CR, D); Barbus serra (EN, D); Barbus sp nov 'Banhine' (CR, U); Barbus stauchi (EN, U); Barbus subinensis (EN, D); Barbus sylvaticus (EN, U); Barbus thysi (EN, U); Barbus traorei (EN, U); Barbus treurensis (EN, S); Barbus trevelyani (EN, U); Barilius canarensis (EN, D); Capoeta pestai (CR, D); Carasobarbus chantrei (EN, D); Catlocarpio siamensis (CR, D); Cephalakompsus pachycheilus (CR,); Chondrostoma beysehirense (EN, U); Chondrostoma fahirae (CR, D); Chondrostoma kinzelbachi (EN, U); Chondrostoma phoxinus (EN, U); Chondrostoma soetta (EN, D); Chrosomus saylori (EN, U); Crossocheilus periyarensis (EN, D); Cyprinella alvarezdelvillari (CR,); Cyprinella bocagrande (CR,); Cyprinella caerulea (EN, D); Cyprinella lepida (EN, D); Cyprinella panarcys (EN,); Cyprinella xanthicara (EN,); Cyprinus barbatus (CR, D); Cyprinus chilia (EN, D); Cyprinus fuxianensis (CR, U); Cyprinus ilishaestomus (CR, D); Cyprinus intha (EN, D); Cyprinus micristius (CR, U); Cyprinus qionghaiensis (CR, D); Cyprinus yunnanensis (CR, U); Danio erythromicron (EN, D); Delminichthys jadovensis (CR, U); Delminichthys krbavensis (CR, U); Devario auropurpureus (EN, D); Devario horai (EN, D); Devario neilgherriensis (EN, D); Devario pathirana (EN, D); Dionda diaboli (EN, U); Epalzeorhynchos bicolor (CR, U); Erimystax cahni (EN, D); Folifer yunnanensis (EN, D); Garra ghorensis (CR, D); Garra hughi (EN, U); Garra kalakadensis (EN, D); Garra surendranathanii (EN, U); Garra trewavasae (CR, U); Gila cypha (EN, D); Gila elegans (CR, D); Gila intermedia (EN, D); Gila modesta (CR,); Gila seminuda (EN, D); Gobio delyamurei (CR, U); Gobio skadarensis (EN, U); Gymnostomus horai (EN, U); Hampala lopezi (CR,); Hemigrammocapoeta kemali (CR, D); Horalabiosa arunachalami (CR, D); Horalabiosa joshuai (EN, D); Hybognathus amarus (EN, U); Hypselobarbus curmuca (EN, D); Hypselobarbus dubius (EN, D); Hypselobarbus micropogon (EN, D); Hypselobarbus mussullah (EN, D); Hypselobarbus periyarensis (EN, D); Hypselobarbus pulchellus (CR, U); Hypselobarbus thomassi (CR, U); Iberochondrostoma almacai (CR, D); Iberochondrostoma lusitanicus (CR, D); Iberochondrostoma olisiponensis (CR, D); Iberochondrostoma oretanum (CR, D); Iberocypris palaciosi (CR, U); Iotichthys phlegethontis (EN, U); Labeo alluaudi (EN, U); Labeobarbus ethiopicus (EN, D); Labeobarbus macrophtalmus (EN, U); Labeobarbus mbami (EN, U); Labeobarbus mungoensis (EN, U); Labeobarbus roylii (EN, U); Labeo curriei (CR, D); Labeo fisheri (EN, U); Labeo lankae (CR,); Labeo mesops (EN, D); Labeo potail (EN, D); Labeo seeberi (EN, D); Laubuca caeruleostigmata (EN, D); Lepidomeda albivallis (CR,); Lepidopygopsis typus (EN, U); Luciobarbus graecus (EN, U); Luciocyprinus striolatus (EN, D); Macrhybopsis tetranema (EN, D); Mandibularca resinus (CR,); Meda fulgida (EN, D); Microrasbora rubescens (EN, D); Moapa coriacea (CR,); Notropis albizonatus (EN, D); Notropis cahabae (EN, D); Notropis mekistocholas (EN, D); Notropis moralesi (CR,); Notropis simus (EN,); Onychostoma alticorpus (EN,); Opsaridium microlepis (EN, D); Ospatulus palaemophagus (EN,); Ospatulus truncatus (CR,); Osteobrama bhimensis (EN, U); Osteochilus longidorsalis (EN, U); Parachondrostoma arrigonis (CR, D); Parachondrostoma turiense (EN, D); Parapsilorhynchus elongatus (EN, U); Parapsilorhynchus prateri (CR, D); Pelasgus epiroticus (CR, U); Pelasgus laconicus (CR, D); Pelasgus prespensis (EN, D); Phoxinellus alepidotus (EN, U); Phoxinellus anatolicus (EN,); Phoxinellus dalmaticus (CR, U); Phoxinus strandjae (EN, U); Phoxinus strymonicus (EN, U); Plagopterus argentissimus (CR, D); Poropuntius bolovenensis (EN, D); Poropuntius chonglingchungi (CR, D); Poropuntius consternans (EN, D); Poropuntius deauratus (EN, D); Poropuntius lobocheiloides (EN, D); Poropuntius solitus (EN, D); Probarbus jullieni (EN, D); Probarbus labeamajor (EN, D); Pseudobarbus afer (EN, D); Pseudobarbus asper (EN, D); Pseudobarbus burchelli (CR, D); Pseudobarbus burgi (EN, D); Pseudobarbus phlegethon (EN, D); Pseudobarbus quathlambae (EN, D); Pseudophoxinus anatolicus (EN, D); Pseudophoxinus battalgili (EN, D); Pseudophoxinus crassus (EN, D); Pseudophoxinus drusensis (EN, D); Pseudophoxinus egridiri (CR, D); Pseudophoxinus handlirschi (CR, U); Pseudophoxinus kervillei (EN, U); Pseudophoxinus punicus (EN, D); Pseudophoxinus syriacus (CR, D); Pseudophoxinus zeregi (CR, U); Ptychidio jordani (CR, D); Puntius amarus (CR,); Puntius arulius (EN, D); Puntius asoka (EN,); Puntius bandula (CR,); Puntius baoulan (CR,); Puntius cauveriensis (EN, U); Puntius chalakkudiensis (EN, D); Puntius clemensi (CR,); Puntius compressiformis (CR, D); Puntius crescentus (EN, U); Puntius deccanensis (CR, U); Puntius denisonii (EN, D); Puntius disa (CR,); Puntius exclamatio (EN, U); Puntius flavifuscus (CR,); Puntius fraseri (EN, D); Puntius herrei (CR, D); Puntius katalo (CR,); Puntius lanaoensis (CR,); Puntius manalak (CR,); Puntius manipurensis (EN, D); Puntius martenstyni (EN,); Puntius ophicephalus (EN, U); Puntius pookodensis (CR, U); Puntius

S7 Table continued

sharmai (EN, U); Puntius tambraparniei (EN, D); Rasbora wilpita (EN,); Relictus solitarius (EN,); Romanogobio benacensis (EN, D); Rutilus meidingeri (EN, D); Rutilus ylikiensis (EN, D); Sawbwa resplendens (EN, D); Scaphognathops theunensis (CR, D); Scardinius graecus (CR, D); Scardinius racovitzai (CR, U); Scardinius scardafa (CR, U); Schismatorhynchos nukta (EN, D); Schizothorax grahami (CR, D); Schizothorax integrilabiatus (CR, U); Schizothorax lepidothorax (EN,); Schizothorax nepalensis (CR, D); Schizothorax raraensis (CR, U); Sinocyclocheilus grahami (CR, D); Sinocyclocheilus tingi (EN, D); Sinocyclocheilus yangzongensis (CR, D); Spratellicypris palata (CR,); Squalius anatolicus (EN, D); Squalius castellanus (EN, D); Squalius keadicus (EN, D); Squalius lucumonis (EN, D); Squalius malacitanus (EN, D); Squalius microlepis (EN, U); Squalius moreoticus (EN, U); Squalius sp nov 'Evia' (CR, U); Squalius tenellus (EN, U); Squalius torgalensis (EN, U); Tampichthys mandibularis (CR,); Telestes beoticus (EN, D); Telestes croaticus (EN, U); Telestes fontinalis (CR, D); Telestes polylepis (CR, U); Telestes sp nov (EN, U); Telestes turskyi (CR, U); Thynnichthys sandkhol (EN, D); Tor kulkarnii (EN, U); Tor malabaricus (EN, D); Tor putitora (EN, D); Trigonostigma somphongsi (CR, D); Varicorhinus platystoma (CR, D); Varicorhinus ruandae (CR, D); Xenocypris yunnanensis (CR, U); Ellopostoma mystax (EN, D); Psilorhynchus microphthalmus (EN, U); Psilorhynchus tenura (CR, D); Pachypanchax sakaramyi (CR, D); Pachypanchax sp nov 'Anjingo' (EN, D); Pachypanchax sp nov 'Varatraza' (EN, U); Aphanius almiriensis (CR, D); Aphanius baeticus (EN, D); Aphanius burduricus (EN,); Aphanius iberus (EN, D); Aphanius saourensis (CR, U); Aphanius sirhani (CR, D); Aphanius splendens (CR,); Aphanius stiassnyae (EN, U); Aphanius transgrediens (CR,); Cualac tessellatus (EN,); Cyprinodon beltrani (EN,); Cyprinodon elegans (EN, U); Cyprinodon fontinalis (EN,); Cyprinodon labiosus (CR, D); Cyprinodon macrolepis (EN,); Cyprinodon maya (EN,); Cyprinodon meeki (CR,); Cyprinodon pachycephalus (CR,); Cyprinodon radiosus (EN, S); Cyprinodon salinus (EN, U); Cyprinodon simus (EN,); Cyprinodon verecundus (CR,); Cyprinodon veronicae (CR,); Fundulus julisia (EN, U); Lucania interioris (CR,); Allotoca diazi (CR, D); Allotoca maculata (CR,); Ataeniobius toweri (EN,); Characodon lateralis (EN,); Girardinichthys viviparus (CR,); Hubbsina turneri (CR,); Ilyodon whitei (CR,); Xenoophorus captivus (EN,); Zoogoneticus tequila (CR, D); Aphyosemion alpha (EN, U); Aphyosemion amoenum (EN, U); Aphyosemion bamilekorum (EN, U); Aphyosemion bualanum (EN, U); Aphyosemion franzwerneri (EN, U); Aphyosemion fulgens (EN, U); Aphyosemion lugens (EN, U); Aphyosemion passaroi (EN, U); Aphyosemion poliaki (EN, U); Aphyosemion tirbaki (EN, U); Aphyosemion volcanum (EN, U); Archiaphyosemion jeanpoli (EN, U); Epiplatys biafranus (EN, U); Epiplatys coccinatus (CR, U); Epiplatys etzeli (EN, U); Epiplatys lokoensis (EN, U); Epiplatys njalaensis (EN, U); Epiplatys roloffi (EN, D); Epiplatys ruhkopfi (CR, U); Fundulopanchax amieti (EN, U); Fundulopanchax arnoldi (EN, U); Fundulopanchax cinnamomeus (EN, U); Fundulopanchax fallax (EN, U); Fundulopanchax marmoratus (EN, U); Fundulopanchax oeseri (EN, U); Fundulopanchax powelli (CR, U); Fundulopanchax rubrolabialis (EN, U); Fundulopanchax scheeli (EN, U); Nothobranchius polli (EN, U); Nothobranchius rosenstocki (EN, U); Nothobranchius symoensi (EN, U); Scriptaphyosemion bertholdi (EN, U); Scriptaphyosemion brueningi (EN, U); Scriptaphyosemion cauveti (CR, U); Scriptaphyosemion etzeli (CR, U); Aplocheilichthys sp nov 'Baringo' (CR, D); Gambusia dominicensis (EN, D); Gambusia eurystoma (CR,); Gambusia nobilis (EN, U); Pantanodon sp nov 'Manombo' (CR, D); Plataplochilus chalcopyrus (EN, U); Plataplochilus terveri (EN, U); Poecilia latipunctata (CR,); Poecilia sulphuraria (CR,); Poropanchax myersi (EN, U); Xiphophorus couchianus (CR,); Xiphophorus gordoni (EN,); Xiphophorus meyeri (EN,); Profundulus hildebrandi (EN, D); Austrolebias cinereus (CR, U); Valencia hispanica (CR, D); Valencia letourneuxi (CR, D); Physiculus helenaensis (CR,); Pungitius hellenicus (CR, D); Tomicodon abuelorum (EN, D); Kneria sp nov 'South Africa' (CR, D); Brachionichthys hirsutus (CR,); Liza luciae (EN,); Eptatretus octatrema (CR, U); Myxine paucidens (EN, U); Paramyxine taiwanae (EN, U); Galaxias fontanus (CR,); Galaxias fuscus (CR,); Galaxias johnstoni (CR,); Galaxias pedderensis (CR,); Hypomesus transpacificus (EN,); Marcusenius meronai (EN, U); Marcusenius victoriae (EN, D); Mormyrus subundulatus (EN, D); Stomatorhinus ivindoensis (EN, U); Scleropages formosus (EN, D); Sandelia bainsii (EN, D); Pterapogon kauderni (EN, D); Badis tuivaiei (EN, U); Salaria economidisi (CR, U); Callionymus sanctaehelenae (CR,); Archoplites interruptus (EN, D); Alcolapia alcalicus (EN, U); Amphilophus margaritifer (EN, D); Amphilophus zaliosus (CR, D); Astatotilapia sp nov 'dwarf bigeye scraper' (CR,); Astatotilapia sp nov 'shovelmouth' (EN,); Benitochromis conjunctus (EN, U); Benitochromis finleyi (EN, U); Benitochromis nigrodorsalis (EN, U); Benitochromis riomuniensis (EN, U); Benitochromis ufermanni (EN, U); Chetia brevis (EN, U); Chetia mola (EN, U); Chromidotilapia linkei (EN, U); Danakilia franchettii (EN, U); Etroplus canarensis (EN, S); Gobiocichla ethelwynnae (EN, U); Haplochromis aelocephalus (CR, D); Haplochromis annectidens (CR,); Haplochromis antleter (CR, D); Haplochromis apogonoides (CR, D); Haplochromis argenteus (CR, D); Haplochromis barbarae (CR, D); Haplochromis bareli (CR, D); Haplochromis beadlei (CR,); Haplochromis brownae (CR, D); Haplochromis cassius (CR, D); Haplochromis cinctus (CR, D); Haplochromis cnester (CR, D); Haplochromis coprologus (CR, D); Haplochromis crassilabris (CR, D); Haplochromis crocopeplus (CR, D); Haplochromis cyaneus (EN, D); Haplochromis dentex (CR, D); Haplochromis desfontainii (EN, U); Haplochromis dichrourus (CR, D); Haplochromis erythromaculatus (EN, D); Haplochromis flaviijosephi (EN, D); Haplochromis flavipinnis (CR, D); Haplochromis granti (CR, D); Haplochromis guiarti (CR, D); Haplochromis heusinkveldi (CR, D); Haplochromis hiatus (CR, D); Haplochromis iris (CR, D); Haplochromis ishmaeli (CR, D); Haplochromis katunzii (CR, D); Haplochromis latifasciatus (CR,); Haplochromis longirostris (CR, D); Haplochromis macrognathus (CR, D); Haplochromis martini (CR, D); Haplochromis michaeli (CR, D); Haplochromis microdon (CR, D); Haplochromis mylergates (CR, D); Haplochromis nanoserranus (CR, D); Haplochromis obesus (CR, D); Haplochromis pancitrinus (CR, D); Haplochromis parvidens (CR, D); Haplochromis percoides (CR, D); Haplochromis perrieri (CR, D); Haplochromis plutonius (CR, D); Haplochromis ptistes (CR, D); Haplochromis pyrrhopteryx (CR, D); Haplochromis simpsoni (EN,); Haplochromis sphex (CR, D); Haplochromis sp nov 'Amboseli' (CR, U); Haplochromis sp nov 'micro-obesus' (CR, D); Haplochromis sp nov 'ruby' (CR,); Haplochromis sulphureus (CR, D); Haplochromis teegelaari (CR, D); Haplochromis teunisrasi (CR, D); Haplochromis theliodon (CR, D); Haplochromis ushindi (CR, D); Haplochromis venator (EN,); Haplochromis victorianus (CR, D); Haplochromis vonlinnei (CR, D); Haplochromis xenostoma (CR, D); Hemichromis cerasogaster (EN, U); Herichthys labridens (EN,); Konia dikume (CR, U); Konia eisentrauti (CR, U); Lamprologus kungweensis (CR, U); Lamprologus tumbanus (EN, U); Lethrinops macracanthus (EN, D); Lethrinops micrentodon (EN, U); Lethrinops microdon (EN, U); Lethrinops stridae (EN, U); Limbochromis robertsi (EN, D); Lipochromis sp nov 'backflash cryptodon' (CR,); Lipochromis sp nov 'black cryptodon' (CR,); Lipochromis sp nov 'parvidens-like' (CR,); Lipochromis sp nov 'small obesoid' (CR,); Myaka myaka (CR, U); Nanochromis transvestitus (EN, U); Oreochromis alcalicus (EN, D); Oreochromis amphimelas (EN, D); Oreochromis chungruruensis (CR, D); Oreochromis esculentus (CR, D); Oreochromis hunteri (CR, D); Oreochromis jipe (CR, D); Oreochromis karomo (CR, D); Oreochromis karongae (EN, D); Oreochromis lepidurus (EN, U); Oreochromis lidole (EN, D); Oreochromis mortimeri (CR, D); Oreochromis pangani (CR, D); Oreochromis squamipinnis (EN, D); Oreochromis variabilis (CR, D); Orthochromis kasuluensis (EN, U); Orthochromis luongoensis (EN, U); Orthochromis mazimeroensis (EN, U); Orthochromis mosoensis (EN, U); Orthochromis rubrolabialis (EN, U); Orthochromis uvinzae (CR, U); Oxylapia polli (CR, U); Parananochromis axelrodi (EN, U); Parananochromis ornatus (EN, U); Paratilapia sp nov 'Vevembe' (CR, D); Paretroplus dambabe (EN, D); Paretroplus maculatus (CR, D); Paretroplus maromandia (EN, D); Paretroplus menarambo (CR, U); Paretroplus sp nov 'Sofia' (EN, D); Prognathochromis sp nov 'long snout' (EN,); Ptychochromis inornatus (EN, D); Ptychochromis sp nov 'Green Garaka' (EN, D); Ptychochromis sp nov 'Joba mena' (CR, D); Ptychochromoides betsileanus (CR, U); Ptychochromoides vondrozo (CR, D); Ptyochromis sp nov 'rainbow sheller' (CR,); Ptyochromis sp nov 'Rusinga oral sheller' (CR,); Pundamilia igneopinnis (EN, U); Pungu maclareni (CR, D); Sarotherodon caroli (CR, U); Sarotherodon lohbergeri (CR, U); Sarotherodon steinbachi (CR, U); Serranochromis meridianus (EN, U); Stomatepia mariae (CR, U); Stomatepia pindu (CR, U); Teleogramma brichardi (CR, U); Tilapia bakossiorum (CR, U); Tilapia bemini (CR, U); Tilapia bythobates (CR, U); Tilapia cessiana (CR, U); Tilapia coffea (CR, U); Tilapia deckerti (CR, U); Tilapia flava (CR, U); Tilapia guinasana (CR, U); Tilapia gutturosa (CR, U); Tilapia imbriferna (CR, U); Tilapia kottae (EN, U); Tilapia snyderae (CR, U); Tilapia spongotroktis (CR, U); Tilapia thysi (CR, U); Tristramella sacra (CR, U); Tylochromis microdon (EN, U); Xystichromis sp nov 'Kyoga flameback' (CR,); Clinus spatulatus (EN,); Datnioides pulcher (CR, D); Kribia leonensis (EN, U); Mogurnda furva (CR, D); Mogurnda variegata (CR, D); Typhleotris madagascariensis (EN, U); Typhleotris pauliani (EN, U); Epinephelus akaara (EN, D); Epinephelus drummondhayi (CR, U); Epinephelus striatus (EN, D); Hyporthodus nigritus (CR, U); Akihito futuna (CR, D); Chlamydogobius micropterus (CR,); Chlamydogobius squamigenus (CR,); Economidichthys trichonis (EN, U); Gobiosoma homochroma (EN, D); Gobulus birdsongi (CR, D); Knipowitschia cameliae (CR, U); Knipowitschia ephesi (CR, U); Knipowitschia mermere (CR, D); Knipowitschia milleri (CR, D); Knipowitschia mrakovcici (CR, D); Knipowitschia thessala (EN, U); Pandaka pygmaea (CR,); Pomatoschistus tortonesei (EN, U); Proterorhinus tataricus (CR, U); Rhinogobius lineatus (EN, D); Sicyopterus eudentatus (EN, D); Sicyopterus rapa (EN, U); Sicyopterus sarasini (EN, D); Silhouettea sibayi (EN, U); Smilosicyopus sasali (EN, D); Stiphodon discotorquatus (CR, D); Stiphodon julieni (EN, U); Stiphodon rubromaculatus (CR, U); Weberogobius amadi (CR,); Anisotremus moricandi (EN,); Halichoeres socialis (CR, U); Scarus trispinosus (EN, D); Paraclinus magdalenae (EN, S); Paraclinus walkeri (CR, U); Lates angustifrons (EN, D); Lates macrophthalmus (EN, D); Lates microlepis (EN, D); Terateleotris aspro (EN, U); Betta livida (EN,); Betta miniopinna (CR,); Betta persephone (CR,); Betta

S7 Table continued

simplex (CR, U); Betta spilotogena (CR,); Parosphromenus harveyi (EN,); Maccullochella ikei (EN,); Maccullochella macquariensis (EN,); Nannoperca oxleyana (EN,); Crystallaria cincotta (CR, D); Etheostoma akatulo (EN, D); Etheostoma boschungi (EN, D); Etheostoma chermocki (CR, D); Etheostoma chienense (EN, U); Etheostoma ditrema (EN, D); Etheostoma fonticola (EN, U); Etheostoma moorei (EN, D); Etheostoma nuchale (EN, U); Etheostoma okaloosae (EN,); Etheostoma phytophilum (EN, D); Etheostoma rubrum (EN, U); Etheostoma scotti (EN, D); Etheostoma susanae (EN, D); Etheostoma tecumsehi (EN, D); Gymnocephalus ambriaelacus (CR, D); Percina antesella (EN, U); Percina aurora (EN, U); Percina brevicauda (EN, U); Percina jenkinsi (CR, D); Percina kusha (EN, U); Romanichthys valsanicola (CR, U); Stereolepis gigas (CR, U); Chaetodontoplus vanderloosi (EN, D); Azurina eupalama (CR, U); Neopomacentrus aquadulcis (EN, D); Protogobius attiti (EN, U); Argyrosomus hololepidotus (EN, U); Bahaba taipingensis (CR, D); Sciaena callaensis (CR, U); Thunnus maccoyii (CR, D); Thunnus thynnus (EN, D); Paralabrax albomaculatus (EN, D); Enneapterygius namarrgon (EN, U); Speoplatyrhinus poulsoni (CR, S); Eudontomyzon hellenicus (CR, U); Lampetra spadicea (CR, D); Hippoglossus hippoglossus (EN,); Atlantoraja castelnaui (EN, D); Bathyraja griseocauda (EN, D); Dasyatis laosensis (EN, D); Dasyatis margarita (EN, D); Himantura kittipongi (EN, U); Himantura oxyrhyncha (EN, U); Himantura polylepis (EN, D); Himantura signifer (EN, U); Pastinachus solocirostris (EN, U); Urogymnus ukpam (EN, U); Aetobatus flagellum (EN, D); Aetomylaeus maculatus (EN, D); Aetomylaeus vespertilio (EN, D); Myliobatis hamlyni (EN, D); Narcine bancroftii (CR, U); Electrolux addisoni (CR, U); Anoxypristis cuspidata (EN, D); Pristis clavata (EN, D); Pristis pristis (CR, D); Pristis zijsron (CR, D); Dipturus laevis (EN, S); Leucoraja melitensis (CR, D); Leucoraja ocellata (EN, D); Malacoraja senta (EN, D); Okamejei pita (CR, U); Raja undulata (EN, D); Rostroraja alba (EN, D); Zearaja maugeana (EN, U); Glaucostegus cemiculus (EN, D); Rhinobatos horkelii (CR, D); Trygonorrhina melaleuca (EN, U); Rhinoptera brasiliensis (EN, D); Urolophus javanicus (CR, D); Urolophus orarius (EN, U); Coregonus bavaricus (CR, U); Coregonus hoferi (CR, U); Coregonus pennantii (CR, U); Coregonus pollan (EN, U); Coregonus reighardi (CR,); Coregonus stigmaticus (EN, U); Coregonus trybomi (CR, D); Coregonus vandesius (EN, U); Hucho bleekeri (CR, D); Hucho hucho (EN, U); Oncorhynchus apache (CR,); Oncorhynchus formosanus (CR,); Oncorhynchus gilae (EN,); Oncorhynchus ishikawai (EN,); Salmo carpio (CR, D); Salmo ezenami (CR, U); Salmo obtusirostris (EN, D); Salmo peristericus (EN, S); Salmo platycephalus (CR, U); Salvelinus grayi (CR, D); Salvelinus japonicus (EN,); Salvelinus lonsdalii (CR, U); Salvelinus obtusus (CR, U); Salvelinus tolmachoffi (EN,); Salvelinus willoughbii (EN, U); Cottus paulus (CR, S); Cottus rondeleti (CR, U); Sebastolobus alascanus (EN,); Sebastes fasciatus (EN,); Sebastes paucispinus (CR,); Amblyceps arunchalensis (EN, U); Liobagrus kingi (EN, U); Liobagrus nigricauda (EN, U); Amphilius caudosignatus (EN, U); Amphilius korupi (EN, U); Amphilius lamani (EN, U); Paramphilius firestonei (EN, U); Arius festinus (EN, D); Arius uncinatus (EN, D); Astroblepus ubidiai (CR, U); Austroglanis barnardi (EN, D); Batasio sharavatiensis (EN, U); Hemibagrus punctatus (CR, D); Horabagrus nigricollaris (EN, U); Pseudobagrus medianalis (CR, D); Lepthoplosternum tordilho (EN, D); Clariallabes mutsindoziensis (EN, U); Clarias cavernicola (CR, U); Clarias maclareni (CR, U); Clarias magur (EN, D); Encheloclarias curtisoma (CR,); Encheloclarias kelioides (CR,); Xenoclarias eupogon (CR, U); Chrysichthys teugelsi (EN, U); Chrysichthys walkeri (EN, D); Liauchenoglanis maculatus (EN, U); Notoglanidium thomasi (EN, U); Parauchenoglanis akiri (EN, U); Parauchenoglanis longiceps (EN, U); Ictalurus pricei (EN, D); Noturus crypticus (CR, U); Noturus fasciatus (EN, U); Noturus gilberti (EN, U); Noturus lachneri (EN, U); Noturus stanauli (EN, U); Noturus taylori (EN, D); Prietella phreatophila (EN,); Malapterurus murrayi (EN, U); Chiloglanis asymetricaudalis (EN, U); Chiloglanis bifurcus (EN, U); Chiloglanis polyodon (CR, U); Chiloglanis ruziziensis (CR, U); Synodontis dekimpei (CR, U); Synodontis dorsomaculatus (EN, U); Synodontis guttatus (EN, U); Synodontis pardalis (EN, U); Pangasianodon hypophthalmus (EN, D); Pangasius sanitwongsei (CR, D); Irvineia voltae (EN, D); Pseudeutropius mitchelli (EN, U); Silonia childreni (EN, D); Ceratoglanis pachynema (CR, D); Pterocryptis barakensis (EN, U); Pterocryptis inusitata (EN, D); Pterocryptis wynaadensis (EN, D); Silurus mento (CR, U); Glyptothorax anamalaiensis (EN, U); Glyptothorax davissinghi (EN, U); Glyptothorax housei (EN, U); Glyptothorax kashmirensis (CR, U); Glyptothorax kudremukhensis (CR, U); Glyptothorax madraspatanus (EN, D); Glyptothorax poonaensis (EN, U); Oreoglanis heteropogon (EN, D); Oreoglanis lepturus (CR, U); Oreoglanis siamensis (EN, D); Trichomycterus venulosus (CR, U); Centrophorus harrissoni (EN, D); Squatina formosa (EN, U); Squatina guggenheim (EN, D); Squatina punctata (EN, D); Pillaia indica (EN, U); Mastacembelus oatesii (EN, U); Monopterus fossorius (EN, U); Ophisternon infernale (EN,); Hippocampus capensis (EN, U); Syngnathus watermeyeri (CR, D)

S7 Table continued

MAMMALIA: Amblysomus marleyi (EN, U); Chrysospalax trevelyani (EN, U); Cryptochloris wintoni (CR, U); Cryptochloris zyli (EN, U); Neamblysomus gunningi (EN, U); Microgale jenkinsae (EN, D); Microgale jobihely (EN, U); Micropotamogale lamottei (EN, D); Galidictis grandidieri (EN, D); Prionailurus planiceps (EN, D); Lontra provocax (EN, D); Lutra sumatrana (EN, D); Mustela lutreola (CR, D); Arctocephalus galapagoensis (EN, D); Neophoca cinerea (EN, D); Zalophus wollebaeki (EN, D); Pusa caspica (EN, D); Procyon pygmaeus (CR, D); Cynogale bennettii (EN, U); Viverra civettina (CR, U); Eubalaena japonica (EN, U); Arabitragus jayakari (EN, D); Bubalus mindorensis (CR, D); Bubalus quarlesi (EN, D); Capra caucasica (EN, D); Cephalophus adersi (CR, D); Cephalophus jentinki (EN, D); Cephalophus spadix (EN, D); Gazella cuvieri (EN, U); Gazella leptoceros (EN, D); Gazella spekei (EN, D); Kobus megaceros (EN, D); Procapra przewalskii (EN, D); Pseudois schaeferi (EN, D); Tragelaphus buxtoni (EN, D); Axis calamianensis (EN, D); Axis kuhlii (CR, S); Dama mesopotamica (EN, I); Muntiacus vuquangensis (EN, D); Rucervus eldii (EN, D); Rusa alfredi (EN, D); Cephalorhynchus hectori (EN, D); Moschus anhuiensis (EN, D); Moschus berezovskii (EN, D); Moschus chrysogaster (EN, D); Moschus cupreus (EN, D); Moschus fuscus (EN, D); Moschus leucogaster (EN, D); Sus cebifrons (CR, D); Sus oliveri (EN, D); Sus verrucosus (EN, D); Catagonus wagneri (EN, D); Tragulus nigricans (EN, D); Balantiopteryx infusca (EN, D); Coleura seychellensis (CR, D); Emballonura semicaudata (EN, D); Amorphochilus schnablii (EN, D); Hipposideros durgadasi (EN, D); Hipposideros halophyllus (EN, D); Hipposideros hypophyllus (EN, D); Hipposideros lamottei (CR, D); Hipposideros orbiculus (EN, D); Eumops floridanus (CR, D); Tadarida bregullae (EN, D); Tadarida tomensis (EN, D); Pteronotus paraguanensis (CR, D); Mystacina robusta (CR, U); Natalus jamaicensis (CR, D); Natalus primus (CR, D); Artibeus incomitatus (CR, D); Leptonycteris nivalis (EN, D); Lonchorhina fernandezi (EN, D); Lonchorhina marinkellei (EN, D); Platyrrhinus chocoensis (EN, D); Sturnira nana (EN, U); Acerodon humilis (EN, D); Aproteles bulmerae (CR, D); Dobsonia chapmani (CR, D); Latidens salimalii (EN, D); Mirimiri acrodonta (CR, D); Myonycteris brachycephala (EN, D); Neopteryx frosti (EN, D); Nyctimene rabori (EN, D); Pteralopex anceps (EN, D); Pteralopex atrata (EN, D); Pteralopex flanneryi (CR, D); Pteralopex pulchra (CR, D); Pteralopex taki (EN, D); Pteropus aruensis (CR, U); Pteropus cognatus (EN, D); Pteropus fundatus (EN, D); Pteropus insularis (CR, U); Pteropus livingstonii (EN, D); Pteropus mariannus (EN, D); Pteropus melanopogon (EN, D); Pteropus nitendiensis (EN, D); Pteropus pohlei (EN, D); Pteropus pselaphon (CR, D); Pteropus rodricensis (CR, I); Pteropus tuberculatus (CR, U); Rhinolophus cognatus (EN, D); Rhinolophus hilli (CR, D); Rhinolophus maclaudi (EN, D); Rhinolophus madurensis (EN, D); Rhinolophus ziama (EN, D); Chalinolobus neocaledonicus (EN, D); Eptesicus japonensis (EN, D); Eptesicus malagasyensis (EN, U); Kerivoula africana (EN, D); Miniopterus fuscus (EN, D); Miniopterus robustior (EN, U); Murina ryukyuana (EN, D); Murina tenebrosa (CR, U); Myotis findleyi (EN, D); Myotis hajastanicus (CR, U); Myotis peninsularis (EN, D); Myotis planiceps (EN, D); Myotis pruinosus (EN, D); Myotis yanbarensis (CR, D); Nyctalus azoreum (EN, D); Nyctophilus howensis (CR, U); Nyctophilus nebulosus (CR, D); Pharotis imogene (CR, U); Pipistrellus endoi (EN, D); Pipistrellus maderensis (EN, D); Pipistrellus murrayi (CR, D); Plecotus teneriffae (EN, D); Rhogeessa genowaysi (EN, D); Pseudantechinus mimulus (EN, D); Sminthopsis aitkeni (CR, D); Sminthopsis psammophila (EN, D); Marmosops handleyi (CR, D); Monodelphis unistriata (CR, U); Dendrolagus goodfellowi (EN, D); Dendrolagus matschiei (EN, D); Dendrolagus mayri (CR, U); Dendrolagus mbaiso (EN, D); Dendrolagus notatus (EN, D); Dendrolagus pulcherrimus (CR, D); Dendrolagus scottae (CR, D); Dorcopsis atrata (CR, D); Lagostrophus fasciatus (EN, U); Petrogale persephone (EN, D); Thylogale calabyi (EN, D); Thylogale lanatus (EN, D); Dactylopsila tatei (EN, U); Gymnobelideus leadbeateri (EN, D); Petaurus abidi (CR, D); Petaurus gracilis (EN, D); Ailurops melanotis (CR, D); Phalanger alexandrae (EN, D); Phalanger lullulae (EN, S); Phalanger matanim (CR, U); Spilocuscus rufoniger (CR, D); Spilocuscus wilsoni (CR, D); Bettongia tropica (EN, D); Potorous gilbertii (CR, S); Potorous longipes (EN, U); Lasiorhinus krefftii (CR, S); Neohylomys hainanensis (EN, D); Podogymnura aureospinula (EN, D); Solenodon cubanus (EN, D); Solenodon paradoxus (EN, D); Chimarrogale phaeura (EN, D); Congosorex phillipsorum (CR, D); Crocidura andamanensis (CR, D); Crocidura ansellorum (EN, D); Crocidura baileyi (EN, D); Crocidura bottegoides (EN, D); Crocidura canariensis (EN, D); Crocidura desperata (EN, D); Crocidura harenna (CR, D); Crocidura hikmiya (EN, D); Crocidura jenkinsi (CR, D); Crocidura lanosa (EN, D); Crocidura miya (EN, D); Crocidura negrina (EN, D); Crocidura nicobarica (CR, D); Crocidura orii (EN, D); Crocidura phaeura (EN, U); Crocidura picea (EN, D); Crocidura stenocephala (EN, D); Crocidura tansaniana (EN, D); Crocidura tarella (EN, D); Crocidura telfordi (EN, D); Crocidura thomensis (EN, D); Crocidura trichura (CR, U); Crocidura usambarae (EN, D); Crocidura wimmeri (CR, U); Cryptotis endersi (EN, U); Cryptotis mera (EN, D); Cryptotis nelsoni (CR, U); Feroculus feroculus (EN, D); Myosorex blarina (EN, D); Myosorex eisentrauti (CR, D); Myosorex geata (EN, D); Myosorex kihaulei (EN, D); Myosorex okuensis (EN, D); Myosorex rumpii (EN, D); Solisorex

S7 Table continued

pearsoni (EN, D); Sorex pribilofensis (EN, U); Sorex sclateri (CR, D); Sorex stizodon (CR, D); Suncus aequatorius (CR, D); Suncus dayi (EN, D); Suncus fellowesgordoni (EN, D); Suncus mertensi (EN, D); Suncus zeylanicus (EN, D); Sylvisorex howelli (EN, D); Sylvisorex isabellae (EN, D); Sylvisorex morio (EN, D); Mogera etigo (EN, D); Bunolagus monticularis (CR, D); Caprolagus hispidus (EN, D); Lepus flavigularis (EN, D); Pentalagus furnessi (EN, D); Sylvilagus graysoni (EN, D); Sylvilagus insonus (EN, U); Sylvilagus mansuetus (CR, D); Sylvilagus robustus (EN, U); Ochotona argentata (CR, D); Ochotona hoffmanni (EN, U); Ochotona iliensis (EN, D); Ochotona koslowi (EN, D); Rhynchocyon chrysopygus (EN, D); Zaglossus attenboroughi (CR, D); Zaglossus bartoni (CR, D); Zaglossus bruijnii (CR, D); Echymipera davidi (EN, U); Peroryctes broadbenti (EN, D); Rhynchomeles prattorum (EN, U); Equus ferus (EN, I); Equus grevyi (EN, S); Manis javanica (EN, D); Manis pentadactyla (EN, D); Bradypus pygmaeus (CR, D); Alouatta pigra (EN, D); Alouatta ululata (EN, D); Ateles fusciceps (CR, D); Lagothrix cana (EN, D); Lagothrix lugens (CR, D); Oreonax flavicauda (CR, D); Callithrix flaviceps (EN, D); Leontopithecus caissara (CR, D); Leontopithecus chrysomelas (EN, D); Leontopithecus chrysopygus (EN, D); Saguinus bicolor (EN, D); Saguinus leucopus (EN, D); Saguinus oedipus (CR, D); Cebus flavius (CR, D); Cebus kaapori (CR, D); Cebus robustus (EN, D); Cebus xanthosternos (CR, D); Cercocebus galeritus (EN, D); Cercocebus sanjei (EN, D); Cercopithecus dryas (CR, U); Cercopithecus preussi (EN, D); Macaca maura (EN, D); Macaca munzala (EN, D); Macaca nigra (CR, D); Macaca pagensis (CR, D); Macaca sinica (EN, D); Presbytis chrysomelas (CR, D); Presbytis comata (EN, D); Presbytis melalophos (EN, D); Presbytis potenziani (EN, D); Procolobus gordonorum (EN, D); Procolobus kirkii (EN, D); Procolobus pennantii (CR, D); Procolobus preussi (CR, D); Pygathrix cinerea (CR, D); Pygathrix nemaeus (EN, D); Pygathrix nigripes (EN, D); Rhinopithecus avunculus (CR, D); Rhinopithecus bieti (EN, D); Rhinopithecus brelichi (EN, D); Rhinopithecus roxellana (EN, D); Rungwecebus kipunji (CR, D); Semnopithecus ajax (EN, D); Simias concolor (CR, D); Trachypithecus delacouri (CR, D); Trachypithecus francoisi (EN, D); Trachypithecus geei (EN, D); Trachypithecus germaini (EN, D); Trachypithecus hatinhensis (EN, D); Trachypithecus phayrei (EN, D); Trachypithecus poliocephalus (CR, D); Trachypithecus shortridgei (EN, D); Trachypithecus vetulus (EN, D); Microcebus berthae (EN, D); Microcebus ravelobensis (EN, D); Microcebus sambiranensis (EN, D); Microcebus tavaratra (EN, D); Galagoides rondoensis (CR, D); Hylobates agilis (EN, D); Hylobates albibarbis (EN, D); Hylobates klossii (EN, D); Hylobates muelleri (EN, D); Hylobates pileatus (EN, D); Nomascus concolor (CR, D); Nomascus gabriellae (EN, D); Nomascus hainanus (CR, S); Nomascus leucogenys (CR, D); Nomascus nasutus (CR, D); Nomascus siki (EN, D); Avahi cleesei (EN, D); Avahi occidentalis (EN, D); Propithecus candidus (CR, D); Propithecus coquereli (EN, D); Propithecus coronatus (EN, D); Propithecus diadema (EN, D); Propithecus edwardsi (EN, D); Propithecus perrieri (CR, D); Propithecus tattersalli (EN, U); Eulemur cinereiceps (EN, D); Eulemur flavifrons (CR, D); Eulemur sanfordi (EN, D); Hapalemur alaotrensis (CR, D); Prolemur simus (CR, D); Varecia rubra (EN, D); Lepilemur ankaranensis (EN, D); Lepilemur septentrionalis (CR, D); Nycticebus javanicus (CR, D); Callicebus barbarabrownae (CR, D); Callicebus caquetensis (CR, D); Callicebus coimbrai (EN, D); Callicebus oenanthe (CR, D); Callicebus olallae (EN, D); Chiropotes albinasus (EN, D); Chiropotes satanas (CR, D); Chiropotes utahickae (EN, D); Tarsius pelengensis (EN, D); Tarsius sangirensis (EN, D); Tarsius tumpara (CR, D); Abrocoma boliviensis (CR, D); Mesocapromys angelcabrerai (EN, U); Mesocapromys auritus (EN, S); Mesocapromys nanus (CR, U); Mesocapromys sanfelipensis (CR, U); Mysateles garridoi (CR, U); Mysateles gundlachi (EN, D); Mysateles meridionalis (CR, D); Plagiodontia aedium (EN, D); Cavia intermedia (CR, D); Chinchilla chinchilla (CR, D); Euryoryzomys lamia (EN, D); Habromys chinanteco (CR, D); Habromys delicatulus (CR, D); Habromys ixtlani (CR, D); Habromys lepturus (CR, D); Habromys schmidlyi (CR, D); Habromys simulatus (EN, D); Kunsia fronto (EN, D); Megadontomys cryophilus (EN, D); Megadontomys nelsoni (EN, D); Megadontomys thomasi (EN, D); Melanomys zunigae (CR, U); Microakodontomys transitorius (EN, D); Microtus bavaricus (CR, D); Microtus oaxacensis (EN, D); Microtus umbrosus (EN, D); Mindomys hammondi (EN, D); Nelsonia goldmani (EN, D); Neotoma angustapalata (EN, D); Neotoma bryanti (EN, D); Neotoma nelsoni (CR, D); Neusticomys mussoi (EN, D); Oryzomys gorgasi (EN, D); Oxymycterus hucucha (EN, D); Oxymycterus josei (EN, D); Peromyscus bullatus (CR, D); Peromyscus caniceps (CR, D); Peromyscus dickeyi (CR, D); Peromyscus guardia (CR, D); Peromyscus interparietalis (CR, D); Peromyscus madrensis (EN, D); Peromyscus mayensis (CR, D); Peromyscus mekisturus (CR, U); Peromyscus melanocarpus (EN, U); Peromyscus melanurus (EN, U); Peromyscus ochraventer (EN, D); Peromyscus pseudocrinitus (CR, D); Peromyscus sejugis (EN, D); Peromyscus slevini (CR, D); Peromyscus stephani (CR, D); Peromyscus winkelmanni (EN, D); Phyllotis definitus (EN, D); Reithrodontomys bakeri (EN, D); Reithrodontomys raviventris (EN, D); Reithrodontomys spectabilis (CR, D); Rheomys mexicanus (EN, U); Sigmodon planifrons (EN, D); Thomasomys hylophilus (EN, D); Thomasomys monochromos (EN, D); Tylomys bullaris (CR, D); Tylomys tumbalensis (CR, D); Wilfredomys oenax (EN, D); Xenomys nelsoni (EN, D); Ctenomys australis (EN, D); Ctenomys bonettoi (EN, D); Ctenomys flamarioni (EN, D); Ctenomys occultus (EN, D); Ctenomys osvaldoreigi (CR, D); Ctenomys pilarensis (EN, D); Ctenomys rionegrensis (EN, D); Ctenomys roigi (CR, D); Ctenomys sociabilis (CR, D); Dasyprocta mexicana (CR, D); Dasyprocta ruatanica (EN, D); Laonastes aenigmamus (EN, D); Sicista armenica (EN, D); Sicista kazbegica (EN, U); Callistomys pictus (EN, D); Phyllomys brasiliensis (EN, D); Phyllomys lundi (EN, D); Phyllomys mantiqueirensis (CR, D); Phyllomys thomasi (EN, D); Phyllomys unicolor (CR, D); Santamartamys rufodorsalis (CR, U); Trinomys eliasi (EN, D); Trinomys moojeni (EN, D); Trinomys yonenagae (EN, D); Geomys tropicalis (CR, D); Orthogeomys lanius (CR, D); Pappogeomys alcorni (CR, D); Zygogeomys trichopus (EN, D); Dipodomys gravipes (CR, U); Dipodomys ingens (EN, D); Dipodomys insularis (CR, D); Dipodomys margaritae (CR, U); Dipodomys stephensi (EN, D); Heteromys nelsoni (EN, D); Heteromys oasicus (EN, D); Liomys spectabilis (EN, D); Perognathus alticolus (EN, D); Apodemus gurkha (EN, D); Batomys russatus (EN, D); Bunomys coelestis (CR, D); Bunomys prolatus (EN, D); Chiropodomys karlkoopmani (EN, D); Crateromys australis (CR, U); Crateromys heaneyi (EN, D); Crateromys schadenbergi (EN, D); Cremnomys elvira (CR, D); Dasymys montanus (EN, D); Desmomys yaldeni (EN, U); Diplothrix legata (EN, D); Echiothrix leucura (EN, D); Gerbillus hesperinus (EN, D); Grammomys gigas (EN, D); Hadromys humei (EN, D); Hapalomys longicaudatus (EN, D); Hybomys badius (EN, D); Hybomys basilii (EN, U); Hylomyscus baeri (EN, D); Hylomyscus grandis (CR, D); Lamottemys okuensis (EN, D); Leopoldamys siporanus (EN, D); Leporillus apicalis (CR, U); Lophuromys dieterleni (EN, D); Lophuromys eisentrauti (EN, D); Lophuromys rahmi (EN, U); Mallomys gunung (EN, D); Maxomys pagensis (EN, D); Maxomys wattsi (EN, D); Melomys aerosus (EN, D); Melomys bannisteri (EN, D); Melomys caurinus (EN, D); Melomys fraterculus (CR, D); Melomys matambuai (EN, D); Melomys rubicola (CR, U); Melomys talaudium (EN, D); Meriones dahli (EN, D); Millardia kondana (CR, D); Mus famulus (EN, D); Mus fernandoni (EN, D); Nesokia bunnii (EN, D); Nesoromys ceramicus (EN, D); Nilopegamys plumbeus (CR, U); Notomys aquilo (EN, D); Otomys barbouri (EN, D); Otomys burtoni (EN, D); Otomys jacksoni (EN, U); Otomys uzungwensis (EN, U); Paraleptomys rufilatus (EN, U); Paramelomys gressitti (EN, U); Paulamys naso (EN, D); Pogonomys fergussoniensis (EN, D); Praomys hartwigi (EN, D); Praomys morio (EN, D); Praomys obscurus (EN, D); Pseudomys fumeus (EN, D); Rattus burrus (EN, D); Rattus hainaldi (EN, D); Rattus lugens (EN, D); Rattus montanus (EN, U); Rattus ranjiniae (EN, D); Rattus simalurensis (EN, D); Rattus vandeuseni (EN, D); Solomys ponceleti (CR, D); Solomys salebrosus (EN, D); Solomys sapientis (EN, D); Sundamys maxi (EN, D); Tokudaia muenninki (CR, D); Tokudaia osimensis (EN, D); Tokudaia tokunoshimensis (EN, D); Uromys boeadii (CR, D); Uromys emmae (CR, U); Uromys imperator (CR, U); Uromys porculus (CR, U); Uromys rex (EN, D); Vandeleuria nilagirica (EN, D); Vandeleuria nolthenii (EN, D); Zyzomys palatalis (CR, U); Zyzomys pedunculatus (CR, U); Brachytarsomys villosa (EN, U); Dendromus kahuziensis (CR, D); Eliurus penicillatus (EN, U); Hypogeomys antimena (EN, D); Macrotarsomys ingens (EN, D); Mystromys albicaudatus (EN, D); Nesomys lambertoni (EN, U); Voalavo antsahabensis (EN, U); Octodon pacificus (CR, D); Pipanacoctomys aureus (CR, D); Salinoctomys loschalchalerosorum (CR, D); Ammospermophilus nelsoni (EN, D); Biswamoyopterus biswasi (CR, D); Cynomys parvidens (EN, D); Eupetaurus cinereus (EN, U); Hylopetes sipora (EN, D); Iomys sipora (EN, D); Marmota sibirica (EN, D); Paraxerus vincenti (EN, D); Petinomys lugens (EN, D); Pteromyscus pulverulentus (EN, D); Spermophilus atricapillus (EN, D); Spermophilus brunneus (EN, D); Spermophilus perotensis (EN, D); Sundasciurus fraterculus (EN, D); Tamiasciurus mearnsi (EN, D); Tamias palmeri (EN, D); Spalax arenarius (EN, D); Tachyoryctes macrocephalus (EN, D); Tupaia chrysogaster (EN, D); Tupaia nicobarica (EN, D)

REPTILIA: Crocodylus mindorensis (CR,); Crocodylus siamensis (CR, D); Calotes liocephalus (EN,); Ceratophora tennentii (EN,); Cophotis dumbara (CR, U); Otocryptis beddomii (EN, S); Phrynocephalus golubewii (CR, D); Phrynocephalus horvathi (CR, D); Cynisca gansi (CR, U); Cynisca kigomensis (CR, U); Cynisca oligopholis (EN, U); Abronia aurita (EN, U); Abronia campbelli (CR, D); Abronia chiszari (EN, D); Abronia deppii (EN, D); Abronia frosti (CR, D); Abronia fuscolabialis (EN, D); Abronia graminea (EN, D); Abronia martindelcampoi (EN, D); Abronia matudai (EN, U); Abronia meledona (EN, U); Abronia montecristoi (EN, D); Abronia salvadorensis (EN, D); Barisia herrerae (EN, D); Barisia rudicollis (EN, S); Celestus anelpistus (CR, D); Celestus bivittatus (EN, D); Celestus warreni (CR, D); Diploglossus montisserrati (CR,); Gerrhonotus parvus (EN, D); Mesaspis juarezi (EN, D); Ophisaurus ceroni (EN, D); Anniella geronimensis (EN, D); Corallus cropanii (EN, U); Epicrates monensis (EN, U); Casarea dussumieri (EN,); Calamaria ingeri (CR, U); Calamaria prakkei (CR, D); Calamaria yunnanensis (EN, U); Pseudorabdion montanum (EN, D); Nephrurus deleani (EN,); Phyllurus gulbaru (CR, U); Archaius tigris (EN, D);

S7 Table continued

Bradypodion caffer (EN, U); Bradypodion taeniabronchum (EN, U); Brookesia bekolosy (EN, D); Brookesia bonsi (CR, D); Brookesia decaryi (EN, D); Brookesia dentata (EN, D); Brookesia exarmata (EN, D); Brookesia karchei (EN, D); Brookesia lineata (EN, U); Brookesia perarmata (EN, D); Brookesia ramanantsoai (EN, D); Brookesia valerieae (EN, U); Calumma andringitraense (EN, D); Calumma furcifer (EN, D); Calumma gallus (EN, D); Calumma glawi (EN, D); Calumma globifer (EN, D); Calumma hafahafa (CR, D); Calumma hilleniusi (EN, D); Calumma tarzan (CR, D); Calumma vencesi (EN, D); Furcifer balteatus (EN, D); Furcifer belalandaensis (CR, D); Furcifer minor (EN, D); Furcifer nicosiai (EN, D); Rhampholeon spinosus (EN, U); Ahaetulla perroteti (EN, U); Boiga bourreti (EN, U); Boiga saengsomi (EN, U); Chironius vincenti (CR,); Euprepiophis perlacea (EN, D); Ficimia hardyi (EN, D); Gongylosoma mukutense (CR,); Hierophis cypriensis (EN, D); Lampropeltis herrerae (CR, D); Lycodon chrysoprateros (CR, D); Masticophis anthonyi (CR, U); Oligodon booliati (CR, U); Oligodon meyerinkii (EN, D); Oxybelis wilsoni (EN, D); Pituophis ruthveni (EN, D); Tantilla flavilineata (EN, S); Tantilla lempira (EN, U); Tantilla oolitica (EN, D); Tantilla shawi (EN, U); Telescopus hoogstraali (EN, D); Cordylus aridus (EN, U); Cordylus meculae (EN, U); Crotaphytus antiquus (EN, D); Gambelia sila (EN, D); Anolis ahli (EN, U); Anolis amplisquamosus (EN, D); Anolis breedlovei (EN, D); Anolis cusuco (EN, S); Anolis guafe (EN, U); Anolis haetianus (EN, U); Anolis hobartsmithi (EN, D); Anolis juangundlachi (CR, U); Anolis koopmani (EN, U); Anolis loveridgei (EN, S); Anolis marron (EN, U); Anolis proboscis (EN, U); Anolis pygmaeus (EN, D); Anolis roosevelti (CR, U); Anolis ruizii (EN, U); Anolis strahmi (EN, U); Bavayia exsuccida (EN, D); Bavayia goroensis (EN, D); Bavayia ornata (EN, U); Eurydactylodes occidentalis (CR, D); Eurydactylodes symmetricus (EN, D); Rhacodactylus trachyrhynchus (EN, U); Adelphicos daryi (EN,); Adelphicos ibarrorum (EN, U); Alsophis antiguae (CR, I); Alsophis rijgersmaei (EN,); Alsophis rufiventris (EN,); Alsophis sanctonum (EN, U); Calamodontophis ronaldoi (EN, U); Chapinophis xanthocheilus (EN, U); Chersodromus rubriventris (EN, D); Enulius roatenensis (EN, D); Geophis damiani (CR, D); Geophis talamancae (EN, U); Liophis cursor (CR,); Liophis ornatus (EN,); Liophis perfuscus (EN,); Liophis williamsi (EN, U); Omoadiphas cannula (CR, D); Omoadiphas texiguatensis (CR, D); Rhadinaea marcellae (EN, D); Rhadinaea montana (EN, D); Rhadinaea stadelmani (EN, U); Rhadinella tolpanorum (CR, D); Trimetopon simile (EN, U); Trimetopon viquezi (CR, U); Aipysurus apraefrontalis (CR, D); Aipysurus foliosquama (CR, D); Aipysurus fuscus (EN, D); Elapsoidea chelazzii (EN, U); Elapsoidea nigra (EN, U); Micrurus ruatanus (CR, D); Ogmodon vitianus (EN, D); Goniurosaurus kuroiwae (EN, U); Cnemaspis anaikattiensis (CR, U); Cnemaspis goaensis (EN, U); Cnemaspis occidentalis (EN, U); Cnemaspis wynadensis (EN, U); Dierogekko inexpectatus (CR, D); Dierogekko kaalaensis (CR, U); Dierogekko koniambo (CR, D); Dierogekko nehoueensis (CR, D); Dierogekko poumensis (CR, S); Dierogekko thomaswhitei (CR, U); Dierogekko validiclavis (EN, U); Ebenavia maintimainty (EN, D); Geckoella jeyporensis (CR, U); Gehyra barea (EN, U); Hemidactylus bouvieri (CR, U); Hemidactylus dracaenacolus (CR, D); Hemidactylus kundaensis (CR, U); Lepidodactylus euaensis (CR, S); Luperosaurus joloensis (EN, D); Luperosaurus macgregori (EN, D); Lygodactylus intermedius (EN, D); Lygodactylus mirabilis (CR, U); Lygodactylus ornatus (EN, U); Lygodactylus roavolana (EN, D); Lygodactylus williamsi (CR, D); Mediodactylus amictopholis (EN, D); Oedodera marmorata (CR, U); Paragehyra gabriellae (EN, D); Paroedura lohatsara (CR, D); Paroedura masobe (EN, D); Paroedura sanctijohannis (EN, U); Paroedura tanjaka (EN, U); Perochirus ateles (EN, D); Phelsuma antanosy (CR, D); Phelsuma flavigularis (EN, D); Phelsuma guentheri (EN,); Phelsuma klemmeri (EN, U); Phelsuma masohoala (CR, U); Phelsuma pronki (CR, D); Phelsuma robertmertensi (EN, U); Phelsuma roesleri (EN, U); Phelsuma seippi (EN, U); Phelsuma serraticauda (EN, D); Phelsuma vanheygeni (EN, U); Uroplatus guentheri (EN, D); Uroplatus malahelo (EN, D); Uroplatus pietschmanni (EN, U); Zonosaurus subunicolor (EN, D); Calyptommatus confusionibus (EN, U); Gymnophthalmus pleii (EN, U); Macropholidus annectens (EN, U); Riama balneator (EN, U); Riama oculata (EN, U); Riama petrorum (EN, U); Cerberus microlepis (EN, D); Enhydris vorisi (EN, U); Brachylophus bulabula (EN, D); Brachylophus fasciatus (EN, D); Brachylophus vitiensis (CR, D); Conolophus marthae (CR, U); Ctenosaura bakeri (CR, D); Ctenosaura melanosterna (EN, D); Ctenosaura oaxacana (CR, D); Ctenosaura oedirhina (EN, D); Ctenosaura palearis (EN, D); Ctenosaura quinquecariniata (EN, D); Cyclura carinata (CR, D); Cyclura collei (CR, U); Cyclura lewisi (EN, I); Cyclura pinguis (CR,); Cyclura ricordii (CR, D); Cyclura rileyi (EN,); Cyclura stejnegeri (EN, D); Iguana delicatissima (EN, D); Acanthodactylus ahmaddisii (EN, D); Acanthodactylus beershebensis (CR, D); Acanthodactylus blanci (EN, D); Acanthodactylus harranensis (CR, D); Acanthodactylus mechriguensis (CR, D); Acanthodactylus schreiberi (EN, D); Acanthodactylus spinicauda (CR, D); Algyroides marchi (EN, D); Darevskia bendimahiensis (EN, U); Darevskia clarkorum (EN, D); Darevskia dryada (CR, D); Darevskia rostombekovi (EN, D); Darevskia uzzelli (EN, D); Eremias pleskei (CR, D); Gallotia auaritae (CR, U); Gallotia bravoana (CR, I); Gallotia intermedia (CR, I); Gallotia simonyi (CR, S); Iberolacerta aranica (EN, D); Iberolacerta aurelioi (EN, D); Iberolacerta cyreni (EN, D); Iberolacerta martinezricai (CR, D); Parvilacerta fraasii (EN, D); Philochortus zolii (EN, D); Phoenicolacerta kulzeri (EN, D); Podarcis carbonelli (EN, D); Podarcis cretensis (EN, D); Podarcis lilfordi (EN, D); Podarcis raffonei (CR, D); Psammodromus microdactylus (EN, D); Takydromus toyamai (EN, U); Lamprophis geometricus (EN, D); Amphiesma metusia (EN, D); Hologerrhum dermali (EN, D); Lycognathophis seychellensis (EN, D); Opisthotropis alcalai (EN, D); Opisthotropis kikuzatoi (CR,); Thamnophis melanogaster (EN, D); Thamnophis mendax (EN, D); Sceloporus chaneyi (EN, D); Sceloporus cyanostictus (EN, D); Sceloporus exsul (CR, D); Sceloporus goldmani (EN, D); Uma exsul (EN, D); Uma inornata (EN, D); Urosaurus auriculatus (EN, D); Tarentola gigas (EN, S); Prosymna ornatissima (CR, U); Alluaudina mocquardi (EN, U); Compsophis vinckei (CR, D); Heteroliodon fohy (EN, D); Liophidium mayottensis (EN, U); Lycodryas guentheri (EN, D); Lycodryas inopinae (EN, U); Phisalixella variabilis (EN, D); Pseudoxyrhopus ankafinaensis (CR, U); Pseudoxyrhopus kely (EN, D); Thamnosophis martae (EN, D); Aprasia aurita (CR, U); Afroablepharus annobonensis (CR, U); Amphiglossus decaryi (EN, U); Brachymeles cebuensis (CR, D); Brachymeles vermis (EN, D); Caledoniscincus auratus (EN, U); Caledoniscincus chazeaui (EN, U); Caledoniscincus orestes (EN, U); Caledoniscincus renevieri (EN, U); Capitellum parvicruzae (CR, D); Celatiscincus euryotis (EN, U); Celatiscincus similis (EN, U); Chalcides ebneri (CR, D); Chalcides mauritanicus (EN, D); Chalcides parallelus (EN, D); Chalcides simonyi (EN, D); Chioninia vaillantii (EN, D); Dasia subcaerulea (EN, U); Emoia adspersa (EN, D); Emoia aneityumensis (EN, D); Emoia boettgeri (EN, D); Emoia campbelli (EN, D); Emoia lawesi (EN, U); Emoia mokosariniveikau (EN, D); Emoia nativitatis (CR, D); Emoia ponapea (EN, D); Emoia samoensis (EN, D); Emoia slevini (CR, D); Emoia trossula (EN, D); Eulamprus leuraensis (EN,); Eurylepis poonaensis (EN, U); Eutropis clivicola (EN, U); Geoscincus haraldmeieri (CR, U); Janetaescincus braueri (EN, D); Janetaescincus veseyfitzgeraldi (EN, D); Kanakysaurus viviparus (EN, U); Kanakysaurus zebratus (EN, U); Lankascincus deignani (EN, U); Leiolopisma alazon (CR, D); Leptosiaphos pauliani (EN, U); Lerista allanae (CR,); Lerista vittata (EN,); Lioscincus maruia (EN, U); Lioscincus steindachneri (EN, U); Lioscincus vivae (CR, U); Madascincus arenicola (CR, U); Madascincus macrolepis (EN, D); Marisora roatanae (CR, D); Marmorosphax kaala (CR, U); Marmorosphax taom (CR, U); Mochlus mafianum (EN, U); Nannoscincus exos (CR, U); Nannoscincus garrulus (EN, U); Nannoscincus greeri (EN, D); Nannoscincus hanchisteus (CR, U); Nannoscincus humectus (EN, U); Nannoscincus manautei (CR, U); Nannoscincus slevini (EN, U); Oligosoma otagense (EN, D); Paracontias fasika (CR, D); Paracontias minimus (CR, D); Paracontias rothschildi (CR, U); Phoboscincus bocourti (EN, U); Plestiodon longirostris (CR,); Pseudoacontias angelorum (EN, U); Pseudoacontias menamainty (CR, U); Pygomeles petteri (EN, D); Scelotes inornatus (EN, U); Sirenoscincus yamagishi (EN, D); Sphenomorphus biparietalis (EN, D); Spondylurus culebrae (CR, D); Spondylurus magnacruzae (CR, D); Spondylurus monae (CR, U); Spondylurus monitae (CR, U); Spondylurus nitidus (EN, D); Spondylurus semitaeniatus (CR, D); Spondylurus sloanii (CR, D); Spondylurus spilonotus (CR, D); Tiliqua adelaidensis (EN,); Voeltzkowia mira (EN, D); Gonatodes daudini (CR, S); Sphaerodactylus armasi (EN, U); Sphaerodactylus micropithecus (EN,); Sphaerodactylus pimienta (EN, U); Sphaerodactylus storeyae (EN, U); Sphaerodactylus williamsi (CR, U); Ameiva polops (CR,); Ameiva vittata (CR, U); Tropidophis hendersoni (CR, U); Liolaemus arambarensis (EN, U); Stenocercus haenschi (CR, U); Afrotyphlops gierrai (EN, U); Ramphotyphlops suluensis (EN, D); Typhlops capitulatus (EN, U); Typhlops hectus (EN, U); Typhlops lazelli (CR, U); Typhlops monensis (EN,); Typhlops schmutzi (EN, U); Typhlops tasymicris (EN, U); Xenotyphlops grandidieri (CR, U); Platyplectrurus madurensis (EN, U); Rhinophis travancoricus (EN, U); Varanus mabitang (EN, D); Bothropoides alcatraz (CR, S); Bothrops lojanus (EN, U); Crotalus catalinensis (CR, D); Crotalus pusillus (EN, U); Crotalus unicolor (CR,); Cryptelytrops kanburiensis (EN, U); Macrovipera schweizeri (EN, S); Mixcoatlus barbouri (EN, D); Mixcoatlus melanurus (EN, D); Montivipera albizona (EN, D); Montivipera bornmuelleri (EN, D); Montivipera latifii (EN, D); Montivipera wagneri (CR, D); Popeia buniana (EN, U); Protobothrops mangshanensis (EN, D); Protobothrops sieversorum (EN, U); Protobothrops trungkhanhensis (EN, U); Vipera anatolica (CR, D); Vipera darevskii (CR, D); Vipera kaznakovi (EN, D); Vipera magnifica (EN, D); Vipera orlovi (CR, D); Vipera pontica (EN, U); Viridovipera truongsonensis (EN, U); Lepidophyma lipetzi (EN, D); Xenosaurus newmanorum (EN, D); Xenosaurus platyceps (EN, D); Chelodina mccordi (CR,); Chelodina pritchardi (EN,); Elseya bellii (EN,); Elusor macrurus (EN,); Mesoclemmys dahli (CR,); Mesoclemmys hogei (EN,); Dermatemys mawii (CR, D); Clemmys guttata (EN, D); Emydoidea blandingii (EN, D); Glyptemys muhlenbergii (CR, U); Graptemys caglei (EN, D); Graptemys gibbonsi (EN, D); Graptemys pearlensis (EN, D); Pseudemys alabamensis (EN,); Terrapene coahuila (EN, D); Trachemys adiutrix (EN,); Trachemys taylori (EN, D); Batagur baska (CR,); Batagur borneoensis (CR,); Batagur dhongoka (EN,); Batagur kachuga (CR,); Batagur trivittata (EN,); Cuora aurocapitata (CR,); Cuora flavomarginata (EN,); Cuora galbinifrons (CR,); Cuora mccordi (CR,); Cuora

S7 Table continued

mouhotii (EN,); Cuora pani (CR,); Cuora trifasciata (CR,); Cuora yunnanensis (CR, D); Cuora zhoui (CR,); Geoemyda japonica (EN,); Geoemyda spengleri (EN,); Heosemys annandalii (EN,); Heosemys depressa (CR,); Heosemys spinosa (EN,); Leucocephalon yuwonoi (CR,); Mauremys annamensis (CR,); Mauremys mutica (EN,); Mauremys nigricans (EN,); Mauremys reevesii (EN, U); Mauremys sinensis (EN,); Orlitia borneensis (EN,); Pangshura sylhetensis (EN,); Sacalia bealei (EN,); Sacalia quadriocellata (EN,); Siebenrockiella leytensis (CR,); Vijayachelys silvatica (EN, U); Sternotherus depressus (CR, D); Platysternon megacephalum (EN,); Erymnochelys madagascariensis (CR, D); Podocnemis lewyana (EN,); Astrochelys radiata (CR, D); Astrochelys yniphora (CR, D); Geochelone platynota (CR,); Indotestudo elongata (EN,); Indotestudo forstenii (EN,); Manouria emys (EN,); Psammobates geometricus (EN,); Pyxis arachnoides (CR, D); Pyxis planicauda (CR, D); Testudo kleinmanni (CR, D); Chitra chitra (CR,); Chitra indica (EN,); Nilssonia formosa (EN,); Palea steindachneri (EN,); Pelochelys cantorii (EN,); Rafetus euphraticus (EN,); Rafetus swinhoei (CR,)
