# Supplementary material for: Popular interest in vertebrates does not reflect extinction risk and is associated with bias in conservation investment
Source: PLoS One. 2018 Sep 26;13(9):e0203694. doi: 10.1371/journal.pone.0203694 (PMC6157853; doi:10.1371/journal.pone.0203694)
Supplement: S8 Table — (PDF) [file pone.0203694.s009.pdf]

S8 Table. Species that were omitted from the analysis due to containing one or more identical common names and having highly correlated monthly web search interest (Pearson's correlation >0.90). It was not possible to establish which of the two of more species that shared a name and were highly correlated were responsible for driving search interest. All pairwise correlations are presented for 141 unique taxa that were omitted from the analysis leading to Figures 2-4, Table 1 and supplementary Tables S1-S6.

1st Species	2nd Species	Pearson's correlation	Shared common names
Caranx vinctus	*Epinephelus adscensionis*	0.95	Jack
Caranx vinctus	*Selene peruviana*	0.96	Jack
Delphinapterus leucas	*Huso huso*	0.96	Beluga
Epinephelus adscensionis	*Caranx vinctus*	0.95	Jack
Epinephelus adscensionis	*Selene peruviana*	0.94	Jack
Lophiodes caulinaris	*Lophius gastrophysus*	0.98	Rape
Lophius gastrophysus	*Lophiodes caulinaris*	0.98	Rape
Selene peruviana	*Caranx vinctus*	0.96	Jack
Selene peruviana	*Epinephelus adscensionis*	0.94	Jack
Lynx lynx	*Lynx rufus*	0.93	Lince
Lynx rufus	*Lynx lynx*	0.93	Lince
Myocastor coypus	*Lutra lutra*	0.95	Nutria
Bunocephalus coracoideus	*Chrysichthys nigrodigitatus*	0.99	Catfish
Bunocephalus coracoideus	*Clarias agboyiensis*	0.99	Catfish
Bunocephalus coracoideus	*Clarias anguillaris*	0.99	Catfish
Bunocephalus coracoideus	*Glyptothorax indicus*	0.99	Catfish
Bunocephalus coracoideus	*Heterobranchus longifilis*	0.99	Catfish
Bunocephalus coracoideus	*Rineloricaria lima*	0.99	Catfish
Bunocephalus coracoideus	*Rineloricaria thrissoceps*	0.99	Catfish
Bunocephalus coracoideus	*Spatuloricaria evansii*	0.99	Catfish
Bunocephalus coracoideus	*Synodontis filamentosus*	0.99	Catfish
Bunocephalus coracoideus	*Synodontis membranaceus*	0.99	Catfish
Bunocephalus coracoideus	*Synodontis nigrita*	0.99	Catfish
Bunocephalus coracoideus	*Synodontis schall*	0.99	Catfish
Chrysichthys nigrodigitatus	*Bunocephalus coracoideus*	0.99	Catfish
Chrysichthys nigrodigitatus	*Clarias agboyiensis*	0.99	Catfish, Mudfish
Chrysichthys nigrodigitatus	*Clarias anguillaris*	0.99	Catfish, Mudfish
Chrysichthys nigrodigitatus	*Glyptothorax indicus*	0.99	Catfish
Chrysichthys nigrodigitatus	*Heterobranchus longifilis*	0.99	Catfish
Chrysichthys nigrodigitatus	*Rineloricaria lima*	0.99	Catfish
Chrysichthys nigrodigitatus	*Rineloricaria thrissoceps*	0.99	Catfish
Chrysichthys nigrodigitatus	*Spatuloricaria evansii*	0.99	Catfish
Chrysichthys nigrodigitatus	*Synodontis filamentosus*	0.99	Catfish
Chrysichthys nigrodigitatus	*Synodontis membranaceus*	0.99	Catfish
Chrysichthys nigrodigitatus	*Synodontis nigrita*	0.99	Catfish
Chrysichthys nigrodigitatus	*Synodontis schall*	0.99	Catfish
Clarias agboyiensis	*Bunocephalus coracoideus*	0.99	Catfish
Clarias agboyiensis	*Chrysichthys nigrodigitatus*	0.99	Catfish, Mudfish
Clarias agboyiensis	*Clarias anguillaris*	0.99	Catfish, Mudfish
Clarias agboyiensis	*Glyptothorax indicus*	0.99	Catfish
Clarias agboyiensis	*Heterobranchus longifilis*	0.99	Catfish
Clarias agboyiensis	*Rineloricaria lima*	0.99	Catfish
Clarias agboyiensis	*Rineloricaria thrissoceps*	0.99	Catfish
Clarias agboyiensis	*Spatuloricaria evansii*	0.99	Catfish
Clarias agboyiensis	*Synodontis filamentosus*	0.99	Catfish
Clarias agboyiensis	*Synodontis membranaceus*	0.99	Catfish
Clarias agboyiensis	*Synodontis nigrita*	0.99	Catfish
Clarias agboyiensis	*Synodontis schall*	0.99	Catfish

S8 Table continued

1st Species	2nd Species	Pearson's correlation	Shared common names
Clarias anguillaris	*Bunocephalus coracoideus*	0.99	Catfish
Clarias anguillaris	*Chrysichthys nigrodigitatus*	0.99	Catfish, Mudfish
Clarias anguillaris	*Clarias agboyiensis*	0.99	Catfish, Mudfish
Clarias anguillaris	*Glyptothorax indicus*	0.99	Catfish
Clarias anguillaris	*Heterobranchus longifilis*	0.99	Catfish
Clarias anguillaris	*Rineloricaria lima*	0.99	Catfish
Clarias anguillaris	*Rineloricaria thrissoceps*	0.99	Catfish
Clarias anguillaris	*Spatuloricaria evansii*	0.99	Catfish
Clarias anguillaris	*Synodontis filamentosus*	0.99	Catfish
Clarias anguillaris	*Synodontis membranaceus*	0.99	Catfish
Clarias anguillaris	*Synodontis nigrita*	0.99	Catfish
Clarias anguillaris	*Synodontis schall*	0.99	Catfish
Glyptothorax indicus	*Bunocephalus coracoideus*	0.99	Catfish
Glyptothorax indicus	*Chrysichthys nigrodigitatus*	0.99	Catfish
Glyptothorax indicus	*Clarias agboyiensis*	0.99	Catfish
Glyptothorax indicus	*Clarias anguillaris*	0.99	Catfish
Glyptothorax indicus	*Heterobranchus longifilis*	0.99	Catfish
Glyptothorax indicus	*Rineloricaria lima*	0.99	Catfish
Glyptothorax indicus	*Rineloricaria thrissoceps*	0.99	Catfish
Glyptothorax indicus	*Spatuloricaria evansii*	0.99	Catfish
Glyptothorax indicus	*Synodontis filamentosus*	0.99	Catfish
Glyptothorax indicus	*Synodontis membranaceus*	0.99	Catfish
Glyptothorax indicus	*Synodontis nigrita*	0.99	Catfish
Glyptothorax indicus	*Synodontis schall*	0.99	Catfish
Heterobranchus longifilis	*Bunocephalus coracoideus*	0.99	Catfish
Heterobranchus longifilis	*Chrysichthys nigrodigitatus*	0.99	Catfish
Heterobranchus longifilis	*Clarias agboyiensis*	0.99	Catfish
Heterobranchus longifilis	*Clarias anguillaris*	0.99	Catfish
Heterobranchus longifilis	*Glyptothorax indicus*	0.99	Catfish
Heterobranchus longifilis	*Rineloricaria lima*	0.99	Catfish
Heterobranchus longifilis	*Rineloricaria thrissoceps*	0.99	Catfish
Heterobranchus longifilis	*Spatuloricaria evansii*	0.99	Catfish
Heterobranchus longifilis	*Synodontis filamentosus*	0.99	Catfish
Heterobranchus longifilis	*Synodontis membranaceus*	0.99	Catfish
Heterobranchus longifilis	*Synodontis nigrita*	0.99	Catfish
Heterobranchus longifilis	*Synodontis schall*	0.99	Catfish
Rineloricaria lima	*Bunocephalus coracoideus*	0.99	Catfish
Rineloricaria lima	*Chrysichthys nigrodigitatus*	0.99	Catfish
Rineloricaria lima	*Clarias agboyiensis*	0.99	Catfish
Rineloricaria lima	*Clarias anguillaris*	0.99	Catfish
Rineloricaria lima	*Glyptothorax indicus*	0.99	Catfish
Rineloricaria lima	*Heterobranchus longifilis*	0.99	Catfish
Rineloricaria lima	*Rineloricaria thrissoceps*	0.99	Catfish, Vieja de agua
Rineloricaria lima	*Spatuloricaria evansii*	0.99	Catfish, Vieja de agua
Rineloricaria lima	*Synodontis filamentosus*	0.99	Catfish
Rineloricaria lima	*Synodontis membranaceus*	0.99	Catfish
Rineloricaria lima	*Synodontis nigrita*	0.99	Catfish

S8 Table continued

1st Species	2nd Species	Pearson's correlation	Shared common names
Rineloricaria lima	*Synodontis schall*	0.99	Catfish
Rineloricaria thrissoceps	*Bunocephalus coracoideus*	0.99	Catfish
Rineloricaria thrissoceps	*Chrysichthys nigrodigitatus*	0.99	Catfish
Rineloricaria thrissoceps	*Clarias agboyiensis*	0.99	Catfish
Rineloricaria thrissoceps	*Clarias anguillaris*	0.99	Catfish
Rineloricaria thrissoceps	*Glyptothorax indicus*	0.99	Catfish
Rineloricaria thrissoceps	*Heterobranchus longifilis*	0.99	Catfish
Rineloricaria thrissoceps	*Rineloricaria lima*	0.99	Catfish, Vieja de agua
Rineloricaria thrissoceps	*Spatuloricaria evansii*	0.99	Catfish, Vieja de agua
Rineloricaria thrissoceps	*Synodontis filamentosus*	0.99	Catfish
Rineloricaria thrissoceps	*Synodontis membranaceus*	0.99	Catfish
Rineloricaria thrissoceps	*Synodontis nigrita*	0.99	Catfish
Rineloricaria thrissoceps	*Synodontis schall*	0.99	Catfish
Spatuloricaria evansii	*Bunocephalus coracoideus*	0.99	Catfish
Spatuloricaria evansii	*Chrysichthys nigrodigitatus*	0.99	Catfish
Spatuloricaria evansii	*Clarias agboyiensis*	0.99	Catfish
Spatuloricaria evansii	*Clarias anguillaris*	0.99	Catfish
Spatuloricaria evansii	*Glyptothorax indicus*	0.99	Catfish
Spatuloricaria evansii	*Heterobranchus longifilis*	0.99	Catfish
Spatuloricaria evansii	*Rineloricaria lima*	0.99	Catfish, Vieja de agua
Spatuloricaria evansii	*Rineloricaria thrissoceps*	0.99	Catfish, Vieja de agua
Spatuloricaria evansii	*Synodontis filamentosus*	0.99	Catfish
Spatuloricaria evansii	*Synodontis membranaceus*	0.99	Catfish
Spatuloricaria evansii	*Synodontis nigrita*	0.99	Catfish
Spatuloricaria evansii	*Synodontis schall*	0.99	Catfish
Synodontis filamentosus	*Bunocephalus coracoideus*	0.99	Catfish
Synodontis filamentosus	*Chrysichthys nigrodigitatus*	0.99	Catfish
Synodontis filamentosus	*Clarias agboyiensis*	0.99	Catfish
Synodontis filamentosus	*Clarias anguillaris*	0.99	Catfish
Synodontis filamentosus	*Glyptothorax indicus*	0.99	Catfish
Synodontis filamentosus	*Heterobranchus longifilis*	0.99	Catfish
Synodontis filamentosus	*Rineloricaria lima*	0.99	Catfish
Synodontis filamentosus	*Rineloricaria thrissoceps*	0.99	Catfish
Synodontis filamentosus	*Spatuloricaria evansii*	0.99	Catfish
Synodontis filamentosus	*Synodontis membranaceus*	0.99	Catfish
Synodontis filamentosus	*Synodontis nigrita*	0.99	Catfish
Synodontis filamentosus	*Synodontis schall*	0.99	Catfish
Synodontis membranaceus	*Bunocephalus coracoideus*	0.99	Catfish
Synodontis membranaceus	*Chrysichthys nigrodigitatus*	0.99	Catfish
Synodontis membranaceus	*Clarias agboyiensis*	0.99	Catfish
Synodontis membranaceus	*Clarias anguillaris*	0.99	Catfish
Synodontis membranaceus	*Glyptothorax indicus*	0.99	Catfish
Synodontis membranaceus	*Heterobranchus longifilis*	0.99	Catfish
Synodontis membranaceus	*Rineloricaria lima*	0.99	Catfish
Synodontis membranaceus	*Rineloricaria thrissoceps*	0.99	Catfish
Synodontis membranaceus	*Spatuloricaria evansii*	0.99	Catfish
Synodontis membranaceus	*Synodontis filamentosus*	0.99	Catfish

S8 Table continued

1st Species	2nd Species	Pearson's correlation	Shared common names
Synodontis membranaceus	*Synodontis nigrita*	0.99	Catfish
Synodontis membranaceus	*Synodontis schall*	0.99	Catfish
Synodontis nigrita	*Bunocephalus coracoideus*	0.99	Catfish
Synodontis nigrita	*Chrysichthys nigrodigitatus*	0.99	Catfish
Synodontis nigrita	*Clarias agboyiensis*	0.99	Catfish
Synodontis nigrita	*Clarias anguillaris*	0.99	Catfish
Synodontis nigrita	*Glyptothorax indicus*	0.99	Catfish
Synodontis nigrita	*Heterobranchus longifilis*	0.99	Catfish
Synodontis nigrita	*Rineloricaria lima*	0.99	Catfish
Synodontis nigrita	*Rineloricaria thrissoceps*	0.99	Catfish
Synodontis nigrita	*Spatuloricaria evansii*	0.99	Catfish
Synodontis nigrita	*Synodontis filamentosus*	0.99	Catfish
Synodontis nigrita	*Synodontis membranaceus*	0.99	Catfish
Synodontis nigrita	*Synodontis schall*	0.99	Catfish
Synodontis schall	*Bunocephalus coracoideus*	0.99	Catfish
Synodontis schall	*Chrysichthys nigrodigitatus*	0.99	Catfish
Synodontis schall	*Clarias agboyiensis*	0.99	Catfish
Synodontis schall	*Clarias anguillaris*	0.99	Catfish
Synodontis schall	*Glyptothorax indicus*	0.99	Catfish
Synodontis schall	*Heterobranchus longifilis*	0.99	Catfish
Synodontis schall	*Rineloricaria lima*	0.99	Catfish
Synodontis schall	*Rineloricaria thrissoceps*	0.99	Catfish
Synodontis schall	*Spatuloricaria evansii*	0.99	Catfish
Synodontis schall	*Synodontis filamentosus*	0.99	Catfish
Synodontis schall	*Synodontis membranaceus*	0.99	Catfish
Synodontis schall	*Synodontis nigrita*	0.99	Catfish
Lutra lutra	*Myocastor coypus*	0.95	Nutria
Anchoa nasus	*Hyporthodus acanthistius*	0.91	Colorado
Huso huso	*Delphinapterus leucas*	0.96	Beluga
Anampses caeruleopunctatus	*Bodianus axillaris*	0.94	Tamarin
Anampses caeruleopunctatus	*Bodianus diana*	0.96	Tamarin
Anampses caeruleopunctatus	*Halichoeres hortulanus*	0.95	Tamarin
Anampses caeruleopunctatus	*Halichoeres scapularis*	0.93	Tamarin
Anampses caeruleopunctatus	*Hemigymnus fasciatus*	0.96	Tamarin
Anampses caeruleopunctatus	*Hologymnosus annulatus*	0.97	Tamarin
Anampses caeruleopunctatus	*Thalassoma hardwicke*	0.95	Tamarin
Anampses caeruleopunctatus	*Thalassoma purpureum*	0.95	Tamarin
Anampses caeruleopunctatus	*Coris formosa*	0.95	Tamarin
Anampses caeruleopunctatus	*Hemigymnus melapterus*	0.96	Tamarin
Bodianus axillaris	*Anampses caeruleopunctatus*	0.94	Tamarin
Bodianus axillaris	*Bodianus diana*	0.96	Tamarin
Bodianus axillaris	*Halichoeres hortulanus*	0.95	Tamarin
Bodianus axillaris	*Halichoeres scapularis*	0.93	Tamarin
Bodianus axillaris	*Hemigymnus fasciatus*	0.97	Tamarin
Bodianus axillaris	*Hologymnosus annulatus*	0.95	Tamarin
Bodianus axillaris	*Thalassoma hardwicke*	0.95	Tamarin
Bodianus axillaris	*Thalassoma purpureum*	0.93	Tamarin

S8 Table continued

1st Species	2nd Species	Pearson's correlation	Shared common names
Bodianus axillaris	*Coris formosa*	0.95	Tamarin
Bodianus axillaris	*Hemigymnus melapterus*	0.96	Tamarin
Bodianus diana	*Anampses caeruleopunctatus*	0.96	Tamarin
Bodianus diana	*Bodianus axillaris*	0.96	Tamarin
Bodianus diana	*Halichoeres hortulanus*	0.96	Tamarin
Bodianus diana	*Halichoeres scapularis*	0.93	Tamarin
Bodianus diana	*Hemigymnus fasciatus*	0.97	Tamarin
Bodianus diana	*Hologymnosus annulatus*	0.95	Tamarin
Bodianus diana	*Thalassoma hardwicke*	0.97	Tamarin
Bodianus diana	*Thalassoma purpureum*	0.95	Tamarin
Bodianus diana	*Coris formosa*	0.96	Tamarin
Bodianus diana	*Hemigymnus melapterus*	0.96	Tamarin
Halichoeres hortulanus	*Anampses caeruleopunctatus*	0.95	Tamarin
Halichoeres hortulanus	*Bodianus axillaris*	0.95	Tamarin
Halichoeres hortulanus	*Bodianus diana*	0.96	Tamarin
Halichoeres hortulanus	*Halichoeres scapularis*	0.93	Tamarin
Halichoeres hortulanus	*Hemigymnus fasciatus*	0.96	Tamarin
Halichoeres hortulanus	*Hologymnosus annulatus*	0.96	Tamarin
Halichoeres hortulanus	*Thalassoma hardwicke*	0.96	Tamarin
Halichoeres hortulanus	*Thalassoma purpureum*	0.95	Tamarin
Halichoeres hortulanus	*Coris formosa*	0.96	Tamarin
Halichoeres hortulanus	*Hemigymnus melapterus*	0.97	Tamarin
Halichoeres scapularis	*Anampses caeruleopunctatus*	0.93	Tamarin
Halichoeres scapularis	*Bodianus axillaris*	0.93	Tamarin
Halichoeres scapularis	*Bodianus diana*	0.93	Tamarin
Halichoeres scapularis	*Halichoeres hortulanus*	0.93	Tamarin
Halichoeres scapularis	*Hemigymnus fasciatus*	0.95	Tamarin
Halichoeres scapularis	*Hologymnosus annulatus*	0.93	Tamarin
Halichoeres scapularis	*Thalassoma hardwicke*	0.95	Tamarin
Halichoeres scapularis	*Thalassoma purpureum*	0.93	Tamarin
Halichoeres scapularis	*Coris formosa*	0.93	Tamarin
Halichoeres scapularis	*Hemigymnus melapterus*	0.95	Tamarin
Hemigymnus fasciatus	*Anampses caeruleopunctatus*	0.96	Tamarin
Hemigymnus fasciatus	*Bodianus axillaris*	0.97	Tamarin
Hemigymnus fasciatus	*Bodianus diana*	0.97	Tamarin
Hemigymnus fasciatus	*Halichoeres hortulanus*	0.96	Tamarin
Hemigymnus fasciatus	*Halichoeres scapularis*	0.95	Tamarin
Hemigymnus fasciatus	*Hologymnosus annulatus*	0.96	Tamarin
Hemigymnus fasciatus	*Thalassoma hardwicke*	0.96	Tamarin
Hemigymnus fasciatus	*Thalassoma purpureum*	0.96	Tamarin
Hemigymnus fasciatus	*Coris formosa*	0.96	Tamarin
Hemigymnus fasciatus	*Hemigymnus melapterus*	0.97	Mamselle Adèle, Tamarin
Hologymnosus annulatus	*Anampses caeruleopunctatus*	0.97	Tamarin
Hologymnosus annulatus	*Bodianus axillaris*	0.95	Tamarin
Hologymnosus annulatus	*Bodianus diana*	0.95	Tamarin
Hologymnosus annulatus	*Halichoeres hortulanus*	0.96	Tamarin
Hologymnosus annulatus	*Halichoeres scapularis*	0.93	Tamarin

S8 Table continued

1st Species	2nd Species	Pearson's correlation	Shared common names
Hologymnosus annulatus	*Hemigymnus fasciatus*	0.96	Tamarin
Hologymnosus annulatus	*Thalassoma hardwicke*	0.96	Tamarin
Hologymnosus annulatus	*Thalassoma purpureum*	0.95	Tamarin
Hologymnosus annulatus	*Coris formosa*	0.96	Tamarin
Hologymnosus annulatus	*Hemigymnus melapterus*	0.96	Tamarin
Pomacanthus arcuatus	*Holacanthus ciliaris*	0.94	Angelfish, Guinea
Thalassoma hardwicke	*Anampses caeruleopunctatus*	0.95	Tamarin
Thalassoma hardwicke	*Bodianus axillaris*	0.95	Tamarin
Thalassoma hardwicke	*Bodianus diana*	0.97	Tamarin
Thalassoma hardwicke	*Halichoeres hortulanus*	0.96	Tamarin
Thalassoma hardwicke	*Halichoeres scapularis*	0.95	Tamarin
Thalassoma hardwicke	*Hemigymnus fasciatus*	0.96	Tamarin
Thalassoma hardwicke	*Hologymnosus annulatus*	0.96	Tamarin
Thalassoma hardwicke	*Thalassoma purpureum*	0.94	Parrotfish, Tamarin
Thalassoma hardwicke	*Coris formosa*	0.96	Tamarin
Thalassoma hardwicke	*Hemigymnus melapterus*	0.97	Tamarin
Thalassoma purpureum	*Anampses caeruleopunctatus*	0.95	Tamarin
Thalassoma purpureum	*Bodianus axillaris*	0.93	Tamarin
Thalassoma purpureum	*Bodianus diana*	0.95	Tamarin
Thalassoma purpureum	*Halichoeres hortulanus*	0.95	Tamarin
Thalassoma purpureum	*Halichoeres scapularis*	0.93	Tamarin
Thalassoma purpureum	*Hemigymnus fasciatus*	0.96	Tamarin
Thalassoma purpureum	*Hologymnosus annulatus*	0.95	Tamarin
Thalassoma purpureum	*Thalassoma hardwicke*	0.94	Parrotfish, Tamarin
Thalassoma purpureum	*Coris formosa*	0.95	Tamarin
Thalassoma purpureum	*Hemigymnus melapterus*	0.95	Tamarin
Sciurus vulgaris	*Tamiasciurus hudsonicus*	0.95	Red Squirrel
Tamiasciurus hudsonicus	*Sciurus vulgaris*	0.95	Red Squirrel
Orectolobus hutchinsi	*Orectolobus maculatus*	1.00	Wobbegong
Orectolobus maculatus	*Orectolobus hutchinsi*	1.00	Wobbegong
Orectolobus maculatus	*Cephaloscyllium isabellum*	0.92	Carpet Shark
Orectolobus maculatus	*Orectolobus ornatus*	0.92	Carpet Shark
Orectolobus maculatus	*Orectolobus reticulatus*	0.92	Carpet Shark
Cephalopholis nigri	*Cephalopholis taeniops*	0.99	Merou
Cephalopholis nigri	*Epinephelus goreensis*	0.99	Merou
Cephalopholis nigri	*Epinephelus guttatus*	0.98	Merou
Cephalopholis nigri	*Hyporthodus mystacinus*	0.99	Merou
Cephalopholis taeniops	*Cephalopholis nigri*	0.99	Merou
Cephalopholis taeniops	*Epinephelus goreensis*	0.98	Merou
Cephalopholis taeniops	*Epinephelus guttatus*	0.98	Merou
Cephalopholis taeniops	*Hyporthodus mystacinus*	0.99	Merou
Cephalopholis taeniops	*Epinephelus analogus*	0.96	Spotted Grouper
Cephalopholis taeniops	*Epinephelus areolatus*	0.93	Spotted Grouper
Cephalopholis taeniops	*Hyporthodus niveatus*	0.91	Spotted Grouper
Epinephelus goreensis	*Cephalopholis nigri*	0.99	Merou
Epinephelus goreensis	*Cephalopholis taeniops*	0.98	Merou
Epinephelus goreensis	*Epinephelus guttatus*	0.97	Merou, Mero

S8 Table continued

1st Species	2nd Species	Pearson's correlation	Shared common names
Epinephelus goreensis	*Epinephelus itajara*	0.98	Mero
Epinephelus goreensis	*Hyporthodus mystacinus*	0.99	Merou, Mero
Epinephelus goreensis	*Acanthistius brasilianus*	0.93	Mero
Epinephelus goreensis	*Alphestes afer*	0.91	Mero
Epinephelus goreensis	*Epinephelus analogus*	0.97	Grouper, Mero
Epinephelus goreensis	*Hyporthodus flavolimbatus*	0.97	Grouper, Mero
Epinephelus goreensis	*Dermatolepis inermis*	0.93	Mero
Epinephelus goreensis	*Epinephelus areolatus*	0.94	Grouper
Epinephelus goreensis	*Epinephelus labriformis*	0.90	Grouper
Epinephelus goreensis	*Epinephelus marginatus*	0.92	Mero
Epinephelus goreensis	*Epinephelus morrhua*	0.93	Grouper
Epinephelus goreensis	*Epinephelus tukula*	0.94	Grouper
Epinephelus goreensis	*Hyporthodus niveatus*	0.93	Mero
Epinephelus goreensis	*Mycteroperca rubra*	0.92	Mero
Epinephelus guttatus	*Cephalopholis nigri*	0.98	Merou
Epinephelus guttatus	*Cephalopholis taeniops*	0.98	Merou
Epinephelus guttatus	*Epinephelus goreensis*	0.97	Merou, Mero
Epinephelus guttatus	*Epinephelus itajara*	0.97	Mero
Epinephelus guttatus	*Hyporthodus mystacinus*	0.98	Merou, Mero
Epinephelus guttatus	*Acanthistius brasilianus*	0.91	Mero
Epinephelus guttatus	*Epinephelus analogus*	0.95	Mero
Epinephelus guttatus	*Hyporthodus flavolimbatus*	0.96	Mero
Epinephelus guttatus	*Dermatolepis inermis*	0.91	Rockhind, Mero
Epinephelus guttatus	*Hyporthodus niveatus*	0.91	Mero
Epinephelus itajara	*Epinephelus goreensis*	0.98	Mero
Epinephelus itajara	*Epinephelus guttatus*	0.97	Mero
Epinephelus itajara	*Hyporthodus mystacinus*	0.99	Guasa, Cherna, Mero
Epinephelus itajara	*Acanthistius brasilianus*	0.92	Mero
Epinephelus itajara	*Alphestes afer*	0.90	Cherna, Cherne, Mero
Epinephelus itajara	*Epinephelus analogus*	0.95	Guato, Mero, Mero Pintado
Epinephelus itajara	*Hyporthodus flavolimbatus*	0.96	Mero
Epinephelus itajara	*Dermatolepis inermis*	0.91	Mero
Epinephelus itajara	*Epinephelus marginatus*	0.90	Mero
Epinephelus itajara	*Hyporthodus niveatus*	0.92	Mero
Epinephelus itajara	*Mycteroperca rubra*	0.91	Mero
Hyporthodus mystacinus	*Cephalopholis nigri*	0.99	Merou
Hyporthodus mystacinus	*Cephalopholis taeniops*	0.99	Merou
Hyporthodus mystacinus	*Epinephelus goreensis*	0.99	Merou, Mero
Hyporthodus mystacinus	*Epinephelus guttatus*	0.98	Merou, Mero
Hyporthodus mystacinus	*Epinephelus itajara*	0.99	Cherna, Guasa, Mero
Hyporthodus mystacinus	*Acanthistius brasilianus*	0.91	Mero
Hyporthodus mystacinus	*Epinephelus analogus*	0.96	Mero
Hyporthodus mystacinus	*Hyporthodus flavolimbatus*	0.96	Mero
Hyporthodus mystacinus	*Dermatolepis inermis*	0.91	Mero
Hyporthodus mystacinus	*Epinephelus marginatus*	0.90	Mero
Hyporthodus mystacinus	*Hyporthodus niveatus*	0.92	Cherna Pintada, Mero
Python molurus	*Python bivittatus*	0.96	Burmese Python

S8 Table continued

1st Species	2nd Species	Pearson's correlation	Shared common names
Python bivittatus	*Python molurus*	0.96	Burmese Python
Pangasianodon gigas	*Pardiglanis tarabinii*	0.98	Giant Catfish
Pardiglanis tarabinii	*Pangasianodon gigas*	0.98	Giant Catfish
Coris formosa	*Anampses caeruleopunctatus*	0.95	Tamarin
Coris formosa	*Bodianus axillaris*	0.95	Tamarin
Coris formosa	*Bodianus diana*	0.96	Tamarin
Coris formosa	*Halichoeres hortulanus*	0.96	Tamarin
Coris formosa	*Halichoeres scapularis*	0.93	Tamarin
Coris formosa	*Hemigymnus fasciatus*	0.96	Tamarin
Coris formosa	*Hologymnosus annulatus*	0.96	Tamarin
Coris formosa	*Thalassoma hardwicke*	0.96	Tamarin
Coris formosa	*Thalassoma purpureum*	0.95	Tamarin
Coris formosa	*Hemigymnus melapterus*	0.95	Tamarin
Hemigymnus melapterus	*Anampses caeruleopunctatus*	0.96	Tamarin
Hemigymnus melapterus	*Bodianus axillaris*	0.96	Tamarin
Hemigymnus melapterus	*Bodianus diana*	0.96	Tamarin
Hemigymnus melapterus	*Halichoeres hortulanus*	0.97	Tamarin
Hemigymnus melapterus	*Halichoeres scapularis*	0.95	Tamarin
Hemigymnus melapterus	*Hemigymnus fasciatus*	0.97	Mamselle Adèle, Tamarin
Hemigymnus melapterus	*Hologymnosus annulatus*	0.96	Tamarin
Hemigymnus melapterus	*Thalassoma hardwicke*	0.97	Tamarin
Hemigymnus melapterus	*Thalassoma purpureum*	0.95	Tamarin
Hemigymnus melapterus	*Coris formosa*	0.95	Tamarin
Holacanthus ciliaris	*Pomacanthus arcuatus*	0.94	Angelfish, Guinea
Acanthistius brasilianus	*Epinephelus goreensis*	0.93	Mero
Acanthistius brasilianus	*Epinephelus guttatus*	0.91	Mero
Acanthistius brasilianus	*Epinephelus itajara*	0.92	Mero
Acanthistius brasilianus	*Hyporthodus mystacinus*	0.91	Mero
Acanthistius brasilianus	*Alphestes afer*	0.94	Mero
Acanthistius brasilianus	*Epinephelus analogus*	0.96	Mero
Acanthistius brasilianus	*Hyporthodus flavolimbatus*	0.96	Mero
Acanthistius brasilianus	*Dermatolepis inermis*	0.94	Mero
Acanthistius brasilianus	*Epinephelus marginatus*	0.95	Mero
Acanthistius brasilianus	*Hyporthodus niveatus*	0.96	Mero
Acanthistius brasilianus	*Mycteroperca rubra*	0.96	Mero
Alphestes afer	*Epinephelus goreensis*	0.91	Mero
Alphestes afer	*Epinephelus itajara*	0.90	Cherna, Cherne, Mero
Alphestes afer	*Acanthistius brasilianus*	0.94	Mero
Alphestes afer	*Epinephelus analogus*	0.95	Mero
Alphestes afer	*Hyporthodus flavolimbatus*	0.95	Mero
Alphestes afer	*Dermatolepis inermis*	0.93	Mutton Hamlet, Mero
Alphestes afer	*Epinephelus marginatus*	0.93	Mero
Alphestes afer	*Hyporthodus niveatus*	0.95	Mero
Alphestes afer	*Mycteroperca rubra*	0.93	Mero
Argentina aliceae	*Argentina sialis*	0.91	Argentina
Argentina sialis	*Argentina aliceae*	0.91	Argentina
Bodianus rufus	*Scyliorhinus capensis*	0.95	Dogfish

S8 Table continued

1st Species	2nd Species	Pearson's correlation	Shared common names
Chaetodon humeralis	*Chaetodon ocellatus*	0.94	Mariposa
Chaetodon humeralis	*Chaetodon striatus*	0.91	Mariposa
Chaetodon humeralis	*Johnrandallia nigrirostris*	0.92	Mariposa
Chaetodon humeralis	*Prognathodes aculeatus*	0.94	Mariposa
Chaetodon ocellatus	*Chaetodon humeralis*	0.94	Mariposa
Chaetodon ocellatus	*Chaetodon striatus*	0.93	Butterbun, School Mistress, Mariposa
Chaetodon ocellatus	*Johnrandallia nigrirostris*	0.94	Mariposa
Chaetodon ocellatus	*Prognathodes aculeatus*	0.93	Butterbun, Mariposa
Chaetodon striatus	*Chaetodon humeralis*	0.91	Mariposa
Chaetodon striatus	*Chaetodon ocellatus*	0.93	Butterbun, School Mistress, Mariposa
Chaetodon striatus	*Johnrandallia nigrirostris*	0.94	Mariposa
Chaetodon striatus	*Prognathodes aculeatus*	0.92	Butterbun, Mariposa
Chimaera cubana	*Prognathodes aculeatus*	0.90	Mariposa
Chimaera monstrosa	*Heterodontus quoyi*	0.96	Gato
Epinephelus analogus	*Cephalopholis taeniops*	0.96	Spotted Grouper
Epinephelus analogus	*Epinephelus goreensis*	0.97	Grouper, Mero
Epinephelus analogus	*Epinephelus guttatus*	0.95	Mero
Epinephelus analogus	*Epinephelus itajara*	0.95	Guato, Mero, Mero Pintado
Epinephelus analogus	*Hyporthodus mystacinus*	0.96	Mero
Epinephelus analogus	*Acanthistius brasilianus*	0.96	Mero
Epinephelus analogus	*Alphestes afer*	0.95	Mero
Epinephelus analogus	*Hyporthodus flavolimbatus*	0.99	Grouper, Mero
Epinephelus analogus	*Dermatolepis inermis*	0.95	Merou Marbre, Mero
Epinephelus analogus	*Epinephelus areolatus*	0.97	Spotted Grouper, Grouper
Epinephelus analogus	*Epinephelus labriformis*	0.94	Grouper, Rock Bass, Cabrilla, Cabrilla Pinta
Epinephelus analogus	*Epinephelus marginatus*	0.94	Mero
Epinephelus analogus	*Epinephelus morrhua*	0.96	Grouper
Epinephelus analogus	*Epinephelus tukula*	0.97	Grouper
Epinephelus analogus	*Hyporthodus niveatus*	0.97	Spotted Grouper, Mero
Epinephelus analogus	*Mycteroperca rubra*	0.95	Mero
Eptatretus polytrema	*Myxine affinis*	0.99	Anguila Babosa, Babosa
Heterodontus quoyi	*Chimaera monstrosa*	0.96	Gato
Hyporthodus flavolimbatus	*Epinephelus goreensis*	0.97	Grouper, Mero
Hyporthodus flavolimbatus	*Epinephelus guttatus*	0.96	Mero
Hyporthodus flavolimbatus	*Epinephelus itajara*	0.96	Mero
Hyporthodus flavolimbatus	*Hyporthodus mystacinus*	0.96	Mero
Hyporthodus flavolimbatus	*Acanthistius brasilianus*	0.96	Mero
Hyporthodus flavolimbatus	*Alphestes afer*	0.95	Mero
Hyporthodus flavolimbatus	*Epinephelus analogus*	0.99	Grouper, Mero
Hyporthodus flavolimbatus	*Dermatolepis inermis*	0.96	Mero
Hyporthodus flavolimbatus	*Epinephelus areolatus*	0.97	Grouper
Hyporthodus flavolimbatus	*Epinephelus labriformis*	0.92	Grouper
Hyporthodus flavolimbatus	*Epinephelus marginatus*	0.95	Mero
Hyporthodus flavolimbatus	*Epinephelus morrhua*	0.95	Grouper
Hyporthodus flavolimbatus	*Epinephelus tukula*	0.97	Grouper
Hyporthodus flavolimbatus	*Hyporthodus niveatus*	0.97	Mero
Hyporthodus flavolimbatus	*Mycteroperca rubra*	0.95	Mero

S8 Table continued

1st Species	2nd Species	Pearson's correlation	Shared common names
Johnrandallia nigrirostris	*Chaetodon humeralis*	0.92	Mariposa
Johnrandallia nigrirostris	*Chaetodon ocellatus*	0.94	Mariposa
Johnrandallia nigrirostris	*Chaetodon striatus*	0.94	Mariposa
Johnrandallia nigrirostris	*Prognathodes aculeatus*	0.94	Mariposa
Mobula mobular	*Mobula thurstoni*	0.94	Manta
Mobula mobular	*Myliobatis chilensis*	0.96	Manta
Mobula mobular	*Myliobatis peruvianus*	0.97	Manta
Mobula thurstoni	*Mobula mobular*	0.94	Manta
Mobula thurstoni	*Myliobatis chilensis*	0.93	Manta
Mobula thurstoni	*Myliobatis peruvianus*	0.93	Manta
Myliobatis chilensis	*Mobula mobular*	0.96	Manta
Myliobatis chilensis	*Mobula thurstoni*	0.93	Manta
Myliobatis chilensis	*Myliobatis peruvianus*	0.96	Manta
Myliobatis peruvianus	*Mobula mobular*	0.97	Manta
Myliobatis peruvianus	*Mobula thurstoni*	0.93	Manta
Myliobatis peruvianus	*Myliobatis chilensis*	0.96	Manta
Myxine affinis	*Eptatretus polytrema*	0.99	Anguila Babosa, Babosa
Paralonchurus peruanus	*Paralonchurus rathbuni*	0.91	Coco
Paralonchurus rathbuni	*Paralonchurus peruanus*	0.91	Coco
Prognathodes aculeatus	*Chaetodon humeralis*	0.94	Mariposa
Prognathodes aculeatus	*Chaetodon ocellatus*	0.93	Butterbun, Mariposa
Prognathodes aculeatus	*Chaetodon striatus*	0.92	Butterbun, Mariposa
Prognathodes aculeatus	*Chimaera cubana*	0.90	Mariposa
Prognathodes aculeatus	*Johnrandallia nigrirostris*	0.94	Mariposa
Scyliorhinus capensis	*Bodianus rufus*	0.95	Dogfish
Stegostoma fasciatum	*Triakis semifasciata*	0.91	Leopard Shark
Triakis semifasciata	*Stegostoma fasciatum*	0.91	Leopard Shark
Ateles geoffroyi	*Cebus macrocephalus*	0.93	Mico
Cebus macrocephalus	*Ateles geoffroyi*	0.93	Mico
Bedotia geayi	*Bedotia longianalis*	0.96	Zona
Bedotia geayi	*Bedotia madagascariensis*	0.97	Zona
Bedotia geayi	*Rheocles lateralis*	0.97	Zona
Bedotia geayi	*Rheocles pellegrini*	0.98	Zona
Bedotia geayi	*Rheocles sikorae*	0.97	Zona
Bedotia geayi	*Rheocles wrightae*	0.96	Zona
Bedotia longianalis	*Bedotia geayi*	0.96	Zona
Bedotia longianalis	*Bedotia madagascariensis*	0.97	Zona
Bedotia longianalis	*Rheocles lateralis*	0.97	Zona
Bedotia longianalis	*Rheocles pellegrini*	0.96	Zona
Bedotia longianalis	*Rheocles sikorae*	0.96	Zona
Bedotia longianalis	*Rheocles wrightae*	0.96	Zona
Bedotia madagascariensis	*Bedotia geayi*	0.97	Zona
Bedotia madagascariensis	*Bedotia longianalis*	0.97	Zona
Bedotia madagascariensis	*Rheocles lateralis*	0.97	Zona
Bedotia madagascariensis	*Rheocles pellegrini*	0.98	Zona
Bedotia madagascariensis	*Rheocles sikorae*	0.97	Zona
Bedotia madagascariensis	*Rheocles wrightae*	0.97	Zona

S8 Table continued

1st Species	2nd Species	Pearson's correlation	Shared common names
Rheocles lateralis	*Bedotia geayi*	0.97	Zona
Rheocles lateralis	*Bedotia longianalis*	0.97	Zona
Rheocles lateralis	*Bedotia madagascariensis*	0.97	Zona
Rheocles lateralis	*Rheocles pellegrini*	0.97	Zona
Rheocles lateralis	*Rheocles sikorae*	0.97	Zona
Rheocles lateralis	*Rheocles wrightae*	0.97	Zona
Rheocles pellegrini	*Bedotia geayi*	0.98	Zona
Rheocles pellegrini	*Bedotia longianalis*	0.96	Zona
Rheocles pellegrini	*Bedotia madagascariensis*	0.98	Zona
Rheocles pellegrini	*Rheocles lateralis*	0.97	Zona
Rheocles pellegrini	*Rheocles sikorae*	0.97	Zona
Rheocles pellegrini	*Rheocles wrightae*	0.97	Zona
Rheocles sikorae	*Bedotia geayi*	0.97	Zona
Rheocles sikorae	*Bedotia longianalis*	0.96	Zona
Rheocles sikorae	*Bedotia madagascariensis*	0.97	Zona
Rheocles sikorae	*Rheocles lateralis*	0.97	Zona
Rheocles sikorae	*Rheocles pellegrini*	0.97	Zona
Rheocles sikorae	*Rheocles wrightae*	0.97	Zona
Rheocles wrightae	*Bedotia geayi*	0.96	Zona
Rheocles wrightae	*Bedotia longianalis*	0.96	Zona
Rheocles wrightae	*Bedotia madagascariensis*	0.97	Zona
Rheocles wrightae	*Rheocles lateralis*	0.97	Zona
Rheocles wrightae	*Rheocles pellegrini*	0.97	Zona
Rheocles wrightae	*Rheocles sikorae*	0.97	Zona
Ambystoma bombypellum	*Ambystoma mexicanum*	0.93	Axolotl
Hyporthodus acanthistius	*Anchoa nasus*	0.91	Colorado
Cephaloscyllium isabellum	*Orectolobus maculatus*	0.92	Carpet Shark
Cephaloscyllium isabellum	*Orectolobus ornatus*	0.96	Carpet Shark
Cephaloscyllium isabellum	*Orectolobus reticulatus*	0.98	Carpet Shark
Dermatolepis inermis	*Epinephelus goreensis*	0.93	Mero
Dermatolepis inermis	*Epinephelus guttatus*	0.91	Rockhind, Mero
Dermatolepis inermis	*Epinephelus itajara*	0.91	Mero
Dermatolepis inermis	*Hyporthodus mystacinus*	0.91	Mero
Dermatolepis inermis	*Acanthistius brasilianus*	0.94	Mero
Dermatolepis inermis	*Alphestes afer*	0.93	Mutton Hamlet, Mero
Dermatolepis inermis	*Epinephelus analogus*	0.95	Merou Marbre, Mero
Dermatolepis inermis	*Hyporthodus flavolimbatus*	0.96	Mero
Dermatolepis inermis	*Epinephelus marginatus*	0.96	Mero
Dermatolepis inermis	*Hyporthodus niveatus*	0.97	Mero
Dermatolepis inermis	*Mycteroperca rubra*	0.95	Mero
Epinephelus areolatus	*Cephalopholis taeniops*	0.93	Spotted Grouper
Epinephelus areolatus	*Epinephelus goreensis*	0.94	Grouper
Epinephelus areolatus	*Epinephelus analogus*	0.97	Grouper, Spotted Grouper
Epinephelus areolatus	*Hyporthodus flavolimbatus*	0.97	Grouper
Epinephelus areolatus	*Epinephelus labriformis*	0.94	Grouper
Epinephelus areolatus	*Epinephelus morrhua*	0.97	Grouper
Epinephelus areolatus	*Epinephelus tukula*	0.98	Grouper

S8 Table continued

1st Species	2nd Species	Pearson's correlation	Shared common names
Epinephelus areolatus	*Hyporthodus niveatus*	0.94	Spotted Grouper
Epinephelus labriformis	*Epinephelus goreensis*	0.90	Grouper
Epinephelus labriformis	*Epinephelus analogus*	0.94	Rock Bass, Grouper, Cabrilla, Cabrilla Pinta
Epinephelus labriformis	*Hyporthodus flavolimbatus*	0.92	Grouper
Epinephelus labriformis	*Epinephelus areolatus*	0.94	Grouper
Epinephelus labriformis	*Epinephelus morrhua*	0.93	Grouper
Epinephelus labriformis	*Epinephelus tukula*	0.96	Grouper
Epinephelus marginatus	*Epinephelus goreensis*	0.92	Mero
Epinephelus marginatus	*Epinephelus itajara*	0.90	Mero
Epinephelus marginatus	*Hyporthodus mystacinus*	0.90	Mero
Epinephelus marginatus	*Acanthistius brasilianus*	0.95	Mero
Epinephelus marginatus	*Alphestes afer*	0.93	Mero
Epinephelus marginatus	*Epinephelus analogus*	0.94	Mero
Epinephelus marginatus	*Hyporthodus flavolimbatus*	0.95	Mero
Epinephelus marginatus	*Dermatolepis inermis*	0.96	Mero
Epinephelus marginatus	*Hyporthodus niveatus*	0.96	Mero
Epinephelus marginatus	*Mycteroperca rubra*	0.94	Mero
Epinephelus morrhua	*Epinephelus goreensis*	0.93	Grouper
Epinephelus morrhua	*Epinephelus analogus*	0.96	Grouper
Epinephelus morrhua	*Hyporthodus flavolimbatus*	0.95	Grouper
Epinephelus morrhua	*Epinephelus areolatus*	0.97	Grouper
Epinephelus morrhua	*Epinephelus labriformis*	0.93	Grouper
Epinephelus morrhua	*Epinephelus tukula*	0.97	Grouper
Epinephelus tukula	*Epinephelus goreensis*	0.94	Grouper
Epinephelus tukula	*Epinephelus analogus*	0.97	Grouper
Epinephelus tukula	*Hyporthodus flavolimbatus*	0.97	Grouper
Epinephelus tukula	*Epinephelus areolatus*	0.98	Grouper
Epinephelus tukula	*Epinephelus labriformis*	0.96	Grouper
Epinephelus tukula	*Epinephelus morrhua*	0.97	Grouper
Hyporthodus niveatus	*Cephalopholis taeniops*	0.91	Spotted Grouper
Hyporthodus niveatus	*Epinephelus goreensis*	0.93	Mero
Hyporthodus niveatus	*Epinephelus guttatus*	0.91	Mero
Hyporthodus niveatus	*Epinephelus itajara*	0.92	Mero
Hyporthodus niveatus	*Hyporthodus mystacinus*	0.92	Cherna Pintada, Mero
Hyporthodus niveatus	*Acanthistius brasilianus*	0.96	Mero
Hyporthodus niveatus	*Alphestes afer*	0.95	Mero
Hyporthodus niveatus	*Epinephelus analogus*	0.97	Spotted Grouper, Mero
Hyporthodus niveatus	*Hyporthodus flavolimbatus*	0.97	Mero
Hyporthodus niveatus	*Dermatolepis inermis*	0.97	Mero
Hyporthodus niveatus	*Epinephelus areolatus*	0.94	Spotted Grouper
Hyporthodus niveatus	*Epinephelus marginatus*	0.96	Mero
Hyporthodus niveatus	*Mycteroperca rubra*	0.96	Mero
Mycteroperca rubra	*Epinephelus goreensis*	0.92	Mero
Mycteroperca rubra	*Epinephelus itajara*	0.91	Mero
Mycteroperca rubra	*Acanthistius brasilianus*	0.96	Mero
Mycteroperca rubra	*Alphestes afer*	0.93	Mero
Mycteroperca rubra	*Epinephelus analogus*	0.95	Mero

S8 Table continued

1st Species	2nd Species	Pearson's correlation	Shared common names
Mycteroperca rubra	*Hyporthodus flavolimbatus*	0.95	Mero
Mycteroperca rubra	*Dermatolepis inermis*	0.95	Mero
Mycteroperca rubra	*Epinephelus marginatus*	0.94	Mero
Mycteroperca rubra	*Hyporthodus niveatus*	0.96	Mero
Orectolobus ornatus	*Orectolobus maculatus*	0.92	Carpet Shark
Orectolobus ornatus	*Cephaloscyllium isabellum*	0.96	Carpet Shark
Orectolobus ornatus	*Orectolobus reticulatus*	0.97	Carpet Shark
Orectolobus reticulatus	*Orectolobus maculatus*	0.92	Carpet Shark
Orectolobus reticulatus	*Cephaloscyllium isabellum*	0.98	Carpet Shark
Orectolobus reticulatus	*Orectolobus ornatus*	0.97	Carpet Shark
Ateles belzebuth	*Ateles chamek*	0.95	Macaco Aranha, Maquisapa
Ateles belzebuth	*Ateles marginatus*	0.91	Macaco Aranha
Ateles chamek	*Ateles belzebuth*	0.95	Macaco Aranha, Maquisapa
Ateles chamek	*Ateles marginatus*	0.94	Macaco Aranha
Ateles marginatus	*Ateles belzebuth*	0.91	Macaco Aranha
Ateles marginatus	*Ateles chamek*	0.94	Macaco Aranha
Callicebus caligatus	*Callicebus cinerascens*	0.98	Titi Monkey
Callicebus caligatus	*Callicebus discolor*	0.98	Titi Monkey
Callicebus caligatus	*Callicebus donacophilus*	0.98	Titi Monkey
Callicebus caligatus	*Callicebus modestus*	0.98	Titi Monkey
Callicebus caligatus	*Callicebus moloch*	0.98	Titi Monkey
Callicebus caligatus	*Callicebus pallescens*	0.96	Titi Monkey
Callicebus cinerascens	*Callicebus caligatus*	0.98	Titi Monkey
Callicebus cinerascens	*Callicebus discolor*	0.98	Titi Monkey
Callicebus cinerascens	*Callicebus donacophilus*	0.98	Titi Monkey
Callicebus cinerascens	*Callicebus modestus*	0.97	Titi Monkey
Callicebus cinerascens	*Callicebus moloch*	0.98	Titi Monkey
Callicebus cinerascens	*Callicebus pallescens*	0.97	Titi Monkey, Zogue-zogue
Callicebus discolor	*Callicebus caligatus*	0.98	Titi Monkey
Callicebus discolor	*Callicebus cinerascens*	0.98	Titi Monkey
Callicebus discolor	*Callicebus donacophilus*	0.97	Titi Monkey
Callicebus discolor	*Callicebus modestus*	0.99	Titi Monkey
Callicebus discolor	*Callicebus moloch*	0.98	Titi Monkey
Callicebus discolor	*Callicebus pallescens*	0.98	Titi Monkey
Callicebus donacophilus	*Callicebus caligatus*	0.98	Titi Monkey
Callicebus donacophilus	*Callicebus cinerascens*	0.98	Titi Monkey
Callicebus donacophilus	*Callicebus discolor*	0.97	Titi Monkey
Callicebus donacophilus	*Callicebus modestus*	0.98	Titi Monkey, Bolivian Titi
Callicebus donacophilus	*Callicebus moloch*	0.98	Titi Monkey
Callicebus donacophilus	*Callicebus pallescens*	0.98	Titi Monkey
Callicebus modestus	*Callicebus caligatus*	0.98	Titi Monkey
Callicebus modestus	*Callicebus cinerascens*	0.97	Titi Monkey
Callicebus modestus	*Callicebus discolor*	0.99	Titi Monkey
Callicebus modestus	*Callicebus donacophilus*	0.98	Bolivian Titi, Titi Monkey
Callicebus modestus	*Callicebus moloch*	0.98	Titi Monkey
Callicebus modestus	*Callicebus pallescens*	0.98	Titi Monkey, Mono Titi
Callicebus moloch	*Callicebus caligatus*	0.98	Titi Monkey

S8 Table continued

1st Species	2nd Species	Pearson's correlation	Shared common names
Callicebus moloch	*Callicebus cinerascens*	0.98	Titi Monkey
Callicebus moloch	*Callicebus discolor*	0.98	Titi Monkey
Callicebus moloch	*Callicebus donacophilus*	0.98	Titi Monkey
Callicebus moloch	*Callicebus modestus*	0.98	Titi Monkey
Callicebus moloch	*Callicebus pallescens*	0.98	Titi Monkey
Callicebus pallescens	*Callicebus caligatus*	0.96	Titi Monkey
Callicebus pallescens	*Callicebus cinerascens*	0.97	Titi Monkey, Zogue-zogue
Callicebus pallescens	*Callicebus discolor*	0.98	Titi Monkey
Callicebus pallescens	*Callicebus donacophilus*	0.98	Titi Monkey
Callicebus pallescens	*Callicebus modestus*	0.98	Titi Monkey, Mono Titi
Callicebus pallescens	*Callicebus moloch*	0.98	Titi Monkey
Ambystoma mexicanum	*Ambystoma bombypellum*	0.93	Axolotl, Ajolote
Genicanthus semicinctus	*Pomacanthus paru*	0.91	Angelfish
Rhinobatos rhinobatos	*Rhynchobatus luebberti*	0.92	Guitarra
Rhynchobatus luebberti	*Rhinobatos rhinobatos*	0.92	Guitarra
Saguinus fuscicollis	*Saguinus nigricollis*	0.97	Leoncito
Saguinus nigricollis	*Saguinus fuscicollis*	0.97	Leoncito
Distocyclus conirostris	*Steatogenys elegans*	0.96	Anguila
Ambystoma maculatum	*Hynobius naevius*	0.91	Spotted Salamander
Hynobius naevius	*Ambystoma maculatum*	0.91	Spotted Salamander
Limnodynastes dorsalis	*Limnodynastes dumerilii*	0.92	Banjo Frog, Pobblebonk
Bodianus insularis	*Thalassoma amblycephalum*	0.97	Parrotfish
Bodianus insularis	*Thalassoma pavo*	0.95	Parrotfish
Centropomus armatus	*Orthopristis chalceus*	0.92	Corcovado
Opisthonema medirastre	*Hemibrycon carrilloi*	0.95	Sardina
Opisthonema medirastre	*Leporinus granti*	0.96	Sardina
Opisthonema medirastre	*Lile piquitinga*	0.91	Sardina
Opisthonema medirastre	*Atherinella argentea*	0.95	Sardina
Orthopristis chalceus	*Centropomus armatus*	0.92	Corcovado
Paralabrax callaensis	*Paralabrax humeralis*	0.94	Sea bass, Cabrilla, Perela
Paralabrax clathratus	*Paralabrax maculatofasciatus*	0.91	Sea bass
Paralabrax humeralis	*Paralabrax callaensis*	0.94	Sea bass, Cabrilla, Perela
Paralabrax maculatofasciatus	*Paralabrax clathratus*	0.91	Sea bass
Pomacanthus paru	*Genicanthus semicinctus*	0.91	Angelfish
Thalassoma amblycephalum	*Bodianus insularis*	0.97	Parrotfish
Thalassoma amblycephalum	*Thalassoma pavo*	0.96	Parrotfish
Thalassoma lutescens	*Cyphomyrus discorhynchus*	0.94	Parrotfish
Thalassoma pavo	*Bodianus insularis*	0.95	Parrotfish
Thalassoma pavo	*Thalassoma amblycephalum*	0.96	Parrotfish
Aquila verreauxii	*Ictinaetus malayensis*	0.90	Black Eagle
Ictinaetus malayensis	*Aquila verreauxii*	0.90	Black Eagle
Cyphomyrus discorhynchus	*Thalassoma lutescens*	0.94	Parrotfish
Hemibrycon carrilloi	*Opisthonema medirastre*	0.95	Sardina
Hemibrycon carrilloi	*Leporinus granti*	0.93	Sardina
Leporinus granti	*Opisthonema medirastre*	0.96	Sardina
Leporinus granti	*Hemibrycon carrilloi*	0.93	Sardina
Leporinus granti	*Atherinella argentea*	0.93	Sardina
Steatogenys elegans	*Distocyclus conirostris*	0.96	Anguila
Lile piquitinga	*Opisthonema medirastre*	0.91	Sardina
Limnodynastes dumerilii	*Limnodynastes dorsalis*	0.92	Banjo Frog, Pobblebonk
Atherinella argentea	*Opisthonema medirastre*	0.95	Sardina
Atherinella argentea	*Leporinus granti*	0.93	Sardina
