# Supplementary material for: Popular interest in vertebrates does not reflect extinction risk and is associated with bias in conservation investment
Source: PLoS One. 2018 Sep 26;13(9):e0203694. doi: 10.1371/journal.pone.0203694 (PMC6157853; doi:10.1371/journal.pone.0203694)
Supplement: S9 Table — (PDF) [file pone.0203694.s010.pdf]

S9 Table. Raw data used in the international aid data analysis (Fig 4). The amount of financial capital given in international aid to the conservation of critically endangered and endangered vertebrate species with high internet search interest. Data were downloaded from the Aid data database (www.aiddata.org) on 08/09/2016. See methods for details of data collection.

Species	Common name	Avereage monthly web serach interest	Aid data (USD)
Acerodon jubatus	Golden-capped Fruit Bat	32.72	0
Achondrostoma salmantinum	Sarda	15.15	0
Addax nasomaculatus	Addax	50.92	16044
Ailuropoda melanoleuca	Giant Panda	8681.54	2400000
Alligator sinensis	Chinese Alligator	21.85	0
Andrias davidianus	Chinese Giant Salamander	27.19	16044
Anodorhynchus hyacinthinus	Hyacinth Macaw	8.99	0
Ardeotis nigriceps	Great Indian Bustard	22.85	5879
Aspidites ramsayi	Ramsay's Python	11.65	0
Ateles hybridus	Variegated Spider Monkey	28.63	86516
Atelopus varius	Harlequin Frog	9.59	46802
Atelopus zeteki	Golden Arrow Poison Frog	21.85	0
Axis porcinus	Hog Deer	26.38	0
Babyrousa togeanensis	Togian Islands Babirusa	21.17	0
Balaenoptera borealis	Sei Whale	43.49	0
Balaenoptera musculus	Blue Whale	1526.06	15393
Balaenoptera physalus	Fin Whale	145.10	0
Balantiocheilos melanopterus	Silver Shark	15.20	0
Beatragus hunteri	Hirola	14.91	56096
Bettongia penicillata	Woylie	20.45	0
Bos javanicus	Banteng	24.56	36569
Bos sauveli	Kouprey	14.30	307321
Bothropoides insularis	Golden Lancehead	16.53	0
Brachyramphus marmoratus	Marbled Murrelet	12.92	0
Brachyteles arachnoides	Muriqui	23.51	0
Brachyteles hypoxanthus	Northern Muriqui	24.59	21393
Bubalus arnee	Asian Buffalo	397.11	2380
Bubalus depressicornis	Anoa	25.57	25771
Burramys parvus	Mountain Pygmy Possum	30.17	0
Cacatua alba	White Cockatoo	10.84	0
Callaeas cinereus	Kokako	15.48	0
Camelus ferus	Bactrian Camel	70.74	10696
Campephilus imperialis	Imperial Woodpecker	14.54	0
Campephilus principalis	Ivory-billed Woodpecker	1421.69	0
Canis rufus	Red Wolf	423.38	0
Canis simensis	Ethiopian Wolf	37.93	0
Capra falconeri	Markhor	22.56	35142
Capra walie	Walia Ibex	11.53	11044
Carduelis cucullata	Red Siskin	8.90	0
Caretta caretta	Loggerhead	155.36	835462
Cheilinus undulatus	Giant Wrasse	38.47	1040
Chelonia mydas	Green Turtle	147.73	308898
Chinchilla lanigera	Chinchilla	38.00	0
Choeropsis liberiensis	Pygmy Hippopotamus	30.33	73892
Conraua goliath	Giant Slippery Frog	37.33	0
Crocodylus intermedius	Orinoco Crocodile	10.10	0
Crocodylus rhombifer	Cuban Crocodile	15.41	0
Ctenosaura flavidorsalis	Yellow-backed Spiny-tailed Iguana	11.51	0
Cuon alpinus	Dhole	69.02	5131

S9 Table continued

Species	Common name	Avereage monthly web serach interest	Aid data (USD)
Cynomys mexicanus	Mexican Prairie Dog	17.88	0
Dasyurus hallucatus	Northern Quoll	13.46	0
Dicerorhinus sumatrensis	Sumatran Rhinoceros	21.21	625104
Diceros bicornis	Black Rhinoceros	99.38	1200000
Dipturus batis	Blue Skate	19.29	0
Elephas maximus	Asian Elephant	417.48	12300000
Enhydra lutris	Sea Otter	395.91	0
Equus africanus	African Wild Ass	248.02	4800000
Equus hemionus	Asiatic Wild Ass	16.63	0
Eretmochelys imbricata	Hawksbill Turtle	32.78	1400000
Eubalaena glacialis	North Atlantic Right Whale	516.21	0
Falco cherrug	Saker Falcon	33.17	0
Galaxias neocaledonicus	Galaxias	10.37	0
Gavialis gangeticus	Gharial	48.01	35730
Geronticus eremita	Northern Bald Ibis	11.50	45481
Glyphis gangeticus	Ganges Shark	19.97	0
Glyphis glyphis	Speartooth Shark	14.83	0
Glyptemys insculpta	Wood Turtle	24.11	0
Gorilla beringei	Eastern Gorilla	181.76	877349
Gorilla gorilla	Western Gorilla	1165.40	218266
Grus americana	Whooping Crane	17.25	0
Gymnogyps californianus	California Condor	115.24	115677
Gymnomyza samoensis	Mao	43.08	5020
Hapalemur aureus	Golden Bamboo Lemur	10.46	0
Harpyhaliaetus coronatus	Crowned Eagle	22.07	0
Hippocamelus bisulcus	Patagonian Huemul	13.76	0
Hoolock hoolock	Western Hoolock Gibbon	14.14	291978
Hucho perryi	Sakhalin Taimen	10.98	0
Hylobates lar	Lar Gibbon	16.57	0
Hylobates moloch	Silvery Javan Gibbon	14.89	3078
Hymenolaimus malacorhynchos	Blue Duck	15.81	0
Hypsirhynchus ater	Black Racer	82.62	0
Indri indri	Indri	18.60	5131
Lathamus discolor	Swift Parrot	11.40	0
Latimeria chalumnae	Coelacanth	15.79	0
Leontopithecus rosalia	Golden Lion Tamarin	86.99	37438
Leopardus jacobita	Andean Cat	22.70	39261
Lepidochelys kempii	Kemp's Ridley	21.93	18655
Leucogeranus leucogeranus	Siberian Crane	10.05	55838
Lipotes vexillifer	Yangtze River Dolphin	75.81	0
Lontra felina	Marine Otter	27.14	3078
Loris tardigradus	Red Slender Loris	40.37	0
Lycaon pictus	African Wild Dog	142.26	179104
Lynx pardinus	Iberian Lynx	45.64	0
Macaca silenus	Lion-tailed Macaque	12.23	0
Macaca sylvanus	Barbary Macaque	14.76	10653
Maccullochella peelii	Murray Cod	17.81	0
Macrocephalon maleo	Maleo	8.38	52731
Mandrillus leucophaeus	Drill	31.03	0
Marmota vancouverensis	Vancouver Island Marmot	25.05	0
Monachus monachus	Mediterranean Monk Seal	23.46	0
Monachus schauinslandi	Hawaiian Monk Seal	10.15	0
Mustela nigripes	Black-footed Ferret	30.51	0

S9 Table continued

Species	Common name	Avereage monthly web serach interest	Aid data (USD)
Mycteroperca fusca	Comb Grouper	28.52	0
Mycteroperca jordani	Gulf Grouper	9.84	0
Myotis sodalis	Indiana Bat	43.21	0
Myrmecobius fasciatus	Numbat	170.00	0
Nanger dama	Dama Gazelle	18.71	25102
Nasalis larvatus	Proboscis Monkey	71.93	0
Neophron percnopterus	Egyptian Vulture	8.17	4800000
Nestor meridionalis	Kaka	23.73	0
Nilgiritragus hylocrius	Nilgiri Tahr	12.34	0
Notarius bonillai	Cazon Sea Catfish	11.08	0
Okapia johnstoni	Okapi	152.27	810106
Onychogalea fraenata	Bridled Nailtail Wallaby	16.83	0
Pagrus pagrus	Common Seabream	7.95	0
Pan paniscus	Bonobo	2105.62	2000000
Panthera tigris	Tiger	29765.26	44900000
Panthera uncia	Snow Leopard	541.30	358263
Pantholops hodgsonii	Chiru	21.49	31530
Pan troglodytes	Chimpanzee	2027.21	2100000
Parantechinus apicalis	Dibbler	17.79	0
Pardofelis badia	Borneo Bay Cat	4.32	0
Perameles bougainville	Western Barred Bandicoot	31.31	0
Petroica traversi	Black Robin	13.52	0
Pezoporus occidentalis	Night Parrot	14.45	0
Phocoena sinus	Vaquita	22.09	0
Phyllobates terribilis	Golden Poison Frog	15.65	9453
Pithecophaga jefferyi	Philippine Eagle	22.20	36595
Platanista gangetica	Ganges River Dolphin	30.58	5131
Pongo abelii	Sumatran Orangutan	25.94	222268
Pongo pygmaeus	Bornean Orangutan	26.04	31480
Porcula salvania	Pygmy Hog	10.91	41628
Porphyrio hochstetteri	Takahe	19.97	0
Prionailurus viverrinus	Fishing Cat	17.88	0
Pristis pectinata	Smalltooth Sawfish	12.71	10040
Procolobus badius	West African Red Colobus	20.01	30381
Psephurus gladius	Chinese Paddlefish	17.11	0
Pseudalopex fulvipes	Darwin's Fox	8.24	0
Pseudemydura umbrina	Western Swamp Tortoise	22.49	0
Pseudophryne corroboree	Corroboree Frog	23.89	0
Pseudoryx nghetinhensis	Saola	19.70	5131
Psittirostra psittacea	Ou	264.78	0
Pteronura brasiliensis	Giant Otter	48.31	4278
Puntius tras	Tras	14.91	0
Rhinoceros sondaicus	Javan Rhinoceros	27.06	444712
Rhinopithecus strykeri	Myanmar Snub-nosed Monkey	5.23	0
Rhynochetos jubatus	Kagu	6.86	0
Romerolagus diazi	Volcano Rabbit	14.05	0
Saiga tatarica	Mongolian Saiga	30.39	128694
Sarcophilus harrisii	Tasmanian Devil	837.98	0
Sarotherodon linnellii	Blackbelly Tilapia	6.91	0
Spheniscus demersus	African Penguin	136.82	10040
Spheniscus mendiculus	Galapagos Penguin	41.29	0
Sphyrna lewini	Scalloped Hammerhead	27.75	25434
Sphyrna mokarran	Great Hammerhead	2981.86	31873

S9 Table continued

Species	Common name	Avereage monthly web serach interest	Aid data (USD)
Squatina aculeata	Sawback Angelshark	7.16	0
Squatina argentina	Argentine Angel Shark	9.72	0
Squatina oculata	Smoothback Angel Shark	15.41	0
Squatina squatina	Angel Shark	400.17	0
Stomatepia mongo	Mongo	20.25	0
Strigops habroptila	Kakapo	63.27	0
Symphalangus syndactylus	Siamang	30.32	143920
Tapirus bairdii	Baird's Tapir	26.84	26396
Tapirus indicus	Asian Tapir	26.01	10262
Tapirus pinchaque	Mountain Tapir	11.61	57192
Telmatobius niger	UCO	9.74	0
Tomistoma schlegelii	False Gharial	6.59	0
Tor khudree	Black Mahseer	11.94	0
Totoaba macdonaldi	Totoaba	35.60	0
Triakis acutipinna	Sharpfin Houndshark	10.61	0
Varecia variegata	Black-and-white Ruffed Lemur	21.58	0
Xanthomyza phrygia	Regent Honeyeater	10.46	0
Zingel asper	Apron	13.24	0
